# The landscape of adult obesity in Malaysia: a mapping of evidence and contemporary narrative review

**DOI:** 10.1016/j.lanwpc.2026.101963

**Published:** 2026-08-27

**Authors:** Quan-Hziung Lim, Jun-Kit Khoo, Ying-Guat Ooi, Anantharaju Ramachandaram, Jeyakantha Ratnasingam

**Affiliations:** a Endocrine Unit, Department of Medicine, Faculty of Medicine, Universiti Malaya, Kuala Lumpur, Malaysia; b Serian Hospital, Sarawak, Malaysia

**Keywords:** Malaysia, Obesity, Overweight, Nutrition, Non-Communicable disease

## Abstract

Malaysia has one of the highest adult overweight/obesity rates (54.4%) regionally, with prevalence projected to keep rising. We mapped and synthesized updated Malaysian adult overweight/obesity research to guide practice and policy. We searched Web of Science, PubMed, and Google Scholar (English, 2016–2024) for studies on adult overweight/obesity in Malaysia. We organized eligible studies into the scopes defined by the national Nutrition Research Priority and conducted thematic narrative synthesis. The evidence base is heavily skewed toward descriptive epidemiology (≈70%), while longitudinal cohorts, experimental designs and health systems research remain scarce. Community-based studies dominate (≈68%), with limited data from clinical or population-based cohorts. Metabolic risk emerges even below BMI 23 kg/m^2^, and waist-to-height ratio >0.5 is a consistent unhealthy threshold. Research on disease burden, economic impact, and long-term outcomes remain limited, constraining policy prioritization. Lifestyle interventions deliver modest effects and are largely confined to micro- and meso-level programmes. Uptake of obesity management medications remains low, bariatric surgery services are expanding but still developing, while policy implementation gaps persist despite existing frameworks. Addressing obesity in Malaysia will require a coordinated, whole-of-society response. Future studies should adopt disease-oriented strategies, harnessing longitudinal national cohorts, pragmatic trials, scalable meso/macro interventions, and a national research collaborative leveraging available resources.

## Background

Obesity is a major global health challenge, affecting 38% of adults in 2020 and projected to exceed 50% by 2035.^1^ Malaysia is an upper-middle income country in the Association of South East Asian Nations (ASEAN) and World Health Organization Western Pacific Region (WHO WPR) with a multiethnic population of 34.1 million.^2^ The latest National Health and Morbidity Survey (NHMS) 2023 reported that Malaysia charted an adult overweight/obesity prevalence of 54.4%, making it 2nd highest in ASEAN and 20th out of 35 in the WPR (Supplementary Table S1).^1^^,^^3^^,^^4^

The Malaysian Ministry of Health has provided a framework for tracking local overweight/obesity research patterns, as a key area of nutrition research priority (NRP), part of the National Plan of Action Nutrition of Malaysia III (NPANM) 2016–2025.^5^ The NRP framework expands into three key purposes and 15 scopes, with over 90 suggested research topics. Two earlier reviews laid important ground work: Lim's narrative review provided broad synthesis of earlier data (2000–2015),^6^ while Mohamad Nor et al. provided a scoping review (2008–2017) aligned with NRP research scopes.^7^ Building upon these previous efforts, the objective of this current review is to collate an updated body of evidence on overweight/obesity research in Malaysia, mapping them against the NRP framework, and provide an updated narrative review of the literature. We aim to provide a platform for future researchers and policymakers alike to access relevant and up-to-date literature on this topic.

## Methods

### Search strategy and selection criteria

We conducted a structured literature search in: Web of Science, PubMed and Google Scholar for English-language publications from January 1, 2015 to December 31, 2024. The search strategy combined Medical Subject Headings (MeSH) and free-text keywords, including: “obesity”, “obesity, abdominal”, and “overweight”, used singly and in combination with the term “Malaysia”.

Titles and abstracts were first screened for relevance, followed by full-text review of potentially eligible articles. Reference lists of included articles were also hand-searched to capture additional relevant publications. Study selection was conducted by two reviewers (JKK and AR) using Rayyan^8^ with disagreements resolved by a third reviewer (QHL).

**Fig. 1:**
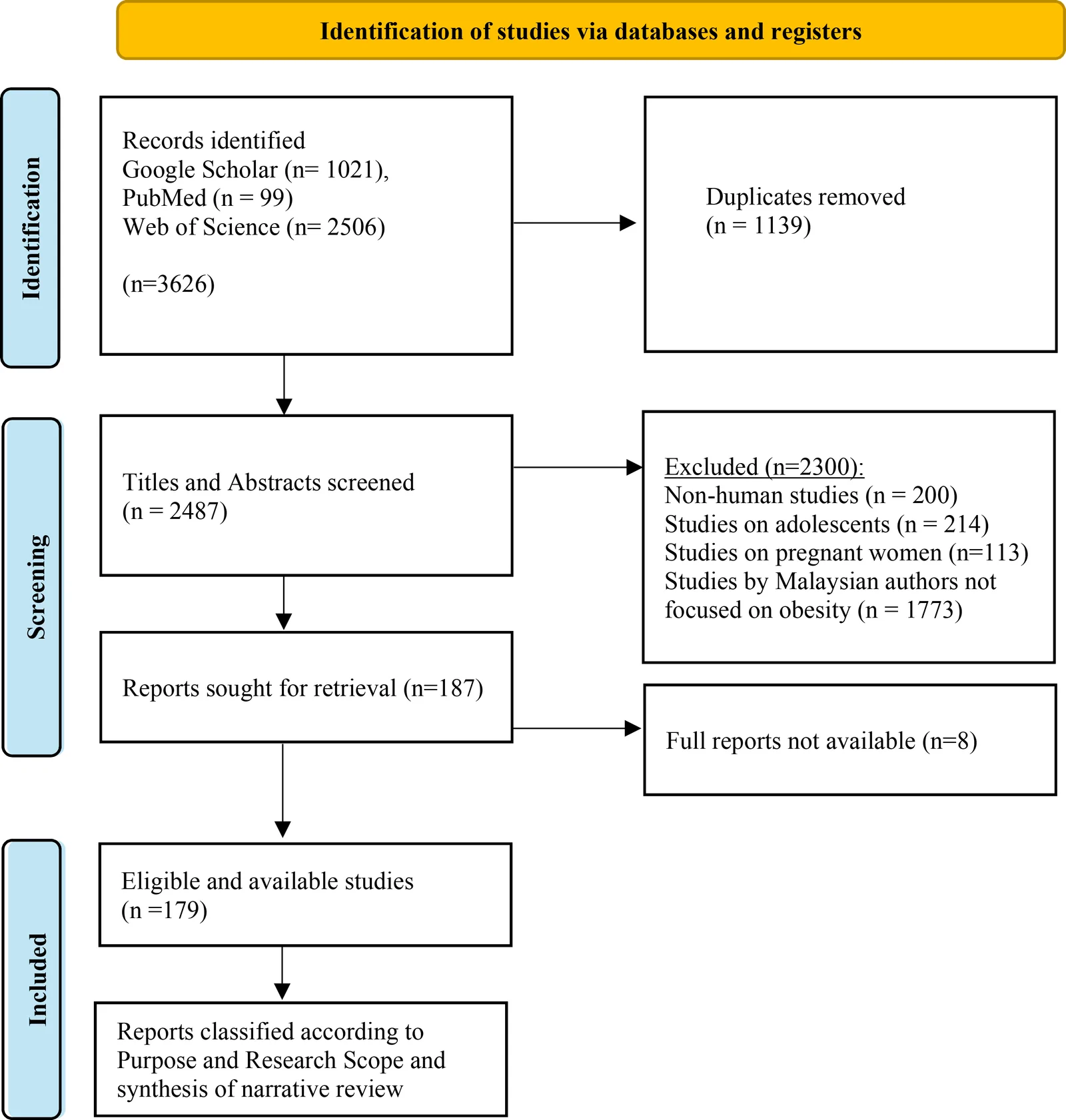
Flow diagram of study selection process on Malaysian studies on adult obesity from 2016 to 2024.

A summary of the search strategy is outlined in the PRISMA flow diagram in Fig. 1. Inclusion criteria comprised primary or secondary studies reporting obesity-focused data among non-pregnant adult Malaysians, or if they addressed related nutrition and health policy issues. Exclusion criteria were i) full text unavailable ii) non-human studies, iii) findings judged to be within outside the scope of the review, iv) studies focused on childhood, adolescent or pregnancy-related obesity issues. In addition to studies identified through the systematic search and selection process, selected government reports, media articles, and key publications outside the predefined search strategy were used to provide contextual background and support the discussion of our initial findings. These sources were not part of the systematic review process and are therefore not included in the study flow diagram.

#### Sorting strategy

The article abstracts and titles were scrutinized independently by two reviewers (JKK and YGO) for relevance to the specific NRP research scopes classified according to the most relevant scope(s). The three main research purposes cited in the NRP comprised: A) to improve the understanding of obesity epidemiology (10 scopes), B) to improve effectiveness of intervention and management of obesity (3 scopes) and C) to develop new modalities (2 scopes). We omitted the scope on ‘impact of early nutrition on development of adult obesity’ (NRP Scope A3), as that mainly covers research on childhood/adolescent obesity. Table 1 compiles the complete list of three research purposes and fourteen scopes.

**Table 1:** Characteristics of studies on obesity in Malaysia from 2016 to 2024, stratified by research purpose and scope (N = 179).

Purpose	Scope	N	Data source	n (%)	Study type	n (%)	Study design	n (%)
A. To improve understanding on the epidemiology of obesity	A1. Relationship between WC, WHR, WHtR and BMI on NCDs.	7	Population	1 (14.3)	Cross Sectional	7 (100)	Observational	7 (100)
							Cohort	0 (0)
			Community	6 (85.7)	Prospective	0 (0)	Single-Arm Interventional	0 (0)
							RCT	0 (0)
			Clinical	0 (0)	Retrospective	0 (0)	Quasi-Experimental	0 (0)
							Mixed/Qualitative	0 (0)
			Others	0 (0)	Others	0 (0)	Review	0 (0)
							Modelling Studies	0 (0)
	A2. Relationship between adiposity and NCDs risk factors	21	Population	6 (28.6)	Cross-Sectional	17 (80.9)	Observational	20 (95.2)
							Cohort	1 (4.8)
			Community	12 (57.1)	Prospective	3 (14.3)	Single-Arm Interventional	0 (0)
							RCT	0 (0)
			Clinical	3 (14.3)	Retrospective	0 (0)	Quasi-Experimental	0 (0)
							Mixed/Qualitative	0 (0)
			Others	0 (0)	Other	1 (4.8)	Review	0 (0)
							Modelling Studies	0 (0)
	A3. The impact of obesity on social and economic cost	10	Population	3 (30.0)	Cross Sectional	4 (40.0)	Observational	4 (40.0)
							Cohort	2 (20)
			Community	6 (60.0)	Prospective	0 (0)	Single-Arm Interventional	1 (10.0)
							RCT	0 (0)
			Clinical	1 (10.0)	Retrospective	2 (20.0)	Quasi-Experimental	0 (0)
							Mixed/Qualitative	0 (0)
			Others	0 (0)	Others	4 (0)	Review	0 (0)
							Modelling Studies	3 (30)
	A4. Association of dietary intake, appetite control, eating behaviour and inflammatory status with obesity	31	Population	4 (12.9)	Cross-Sectional	26 (83.9)	Observational	26 (83.9)
							Cohort	2 (6.5)
			Community	26 (83.9)	Prospective	1 (3.2)	Single-Arm Interventional	0 (0)
							RCT	1 (3.2)
			Clinical	1 (3.2)	Retrospective	2 (6.5)	Quasi-Experimental	0 (0)
							Mixed/Qualitative	0 (0)
			Others	0 (0)	Other	1 (3.2)	Review	1 (3.2)
							Modelling Studies	1 (3.2)
	A5. Determination of Socio-Cultural Factors Influencing Obesity	19	Population	4 (21.0)	Cross Sectional	16 (84.2)	Observational	17 (89.5)
							Cohort	0 (0)
			Community	15 (78.9)	Prospective	0 (0)	Single-Arm Interventional	0 (0)
							RCT	0 (0)
			Clinical	0 (0)	Retrospective	2 (10.5)	Quasi-Experimental	0 (0)
							Mixed/Qualitative	1 (5.3)
			Others	0 (0)	Others	1 (5.3)	Review	0 (0)
							Modelling Studies	1 (5.3)
	A6. Effect of Metabolic Predisposition to Onset of Obesity.	8	Population	4 (40.0)	Cross-Sectional	8 (100)	Observational	8 (100)
							Cohort	0 (0)
			Community	4 (40.0)	Prospective	0 (0)	Single-Arm Interventional	0 (0)
							RCT	0 (0)
			Clinical	0 (0)	Retrospective	0 (0)	Quasi-Experimental	0 (0)
							Mixed/Qualitative	0 (0)
			Others	0 (0)	Other	0 (0)	Review	0 (0)
							Modelling Studies	0 (0)
	A7. Association between Physical Inactivity, Sedentary Lifestyle and Obesity.	17	Population	5 (29.4)	Cross Sectional	15 (88.2)	Observational	16 (94.1)
							Cohort	0 (0)
			Community	11 (64.7)	Prospective	0 (0)	Single-Arm Interventional	0 (0)
							RCT	0 (0)
			Clinical	1 (5.9)	Retrospective	2 (11.8)	Quasi-Experimental	0 (0)
							Mixed/Qualitative	0 (0)
			Others	0 (0)	Others	0 (0)	Review	0 (0)
							Modelling Studies	1 (5.9)
	A8. Determination of Genetic Factors Influencing Development of Overweight and Obesity.	16	Population	0 (0)	Cross-Sectional	15 (93.8)	Observational	15 (93.8)
							Cohort	0 (0)
			Community	15 (93.8)	Prospective	0 (0)	Single-Arm Interventional	0 (0)
							RCT	0 (0)
			Clinical	0 (0)	Retrospective	0 (0)	Quasi-Experimental	0 (0)
							Mixed/Qualitative	0 (0)
			Others	1 (6.2)	Other	1 (6.2)	Review	1 (6.2)
							Modelling Studies	0 (0)
	A9. Obesity and COVID-19	13	Population	0 (0)	Cross Sectional	13 (100)	Observational	13 (100)
							Cohort	0 (0)
			Community	12 (92.3)	Prospective	0 (0)	Single-Arm Interventional	0 (0)
							RCT	0 (0)
			Clinical	1 (7.7)	Retrospective	0 (0)	Quasi-Experimental	0 (0)
							Mixed/Qualitative	0 (0)
			Others	0	Others	0 (0)	Review	0 (0)
							Modelling Studies	0 (0)
B. To Improve Effectiveness of Intervention and Management of Obesity	B1 Development and Evaluation of Obesity Prevention and Intervention Programmes.	16	Population	0 (0)	Cross-Sectional	2 (12.5)	Observational	2 (12.5)
							Cohort	0 (0)
			Community	15 (93.8)	Prospective	14 (87.5)	Single-Arm Interventional	4 (25.0)
							RCT	9 (56.2)
			Clinical	1 (6.2)	Retrospective	0 (0)	Quasi-Experimental	1 (6.2)
							Mixed/Qualitative	0 (0)
			Others	0 (0)	Other	0 (0)	Review	0 (0)
							Modelling Studies	0 (0)
	B2 Development and Evaluation of Obesity Management Programmes.	23	Population	0 (0)	Cross Sectional	2 (8.7)	Observational	2 (8.7)
							Cohort	5 (21.7)
			Community	10 (43.5)	Prospective	20 (87.0)	Single-Arm Interventional	4 (17.4)
							RCT	10 (43.5)
			Clinical	13 (56.5)	Retrospective	1 (4.3)	Quasi-Experimental	1 (4.3)
							Mixed/Qualitative	1 (4.3)
			Others	0 (0)	Others	0 (0)	Review	0 (0)
							Modelling Studies	0 (0)
	B3 The Impact of Policies and Environment (Food and Physical Activity) on Obesity	15	Population	11 (73.3)	Cross-Sectional	9 (60.0)	Observational	6 (40.0)
							Cohort	1 (6.7)
			Community	4 (26.7)	Prospective	2 (13.3)	Single-Arm Interventional	0 (0)
							RCT	1 (6.7)
			Clinical	0 (0)	Retrospective	1 (6.6)	Quasi-Experimental	0 (0)
							Mixed/Qualitative	4 (26.7)
			Others	0 (0)	Other	3 (20.0)	Review	2 (13.3)
							Modelling Studies	1 (6.7)
C To Develop New Modalities	C1 Identification of New Methods to Define Obesity	3	Population	2 (66.7)	Cross Sectional	1 (33.3)	Observational	1 (33.3)
							Cohort	0 (0)
			Community	1 (33.3)	Prospective	0 (0)	Single-Arm Interventional	0 (0)
							RCT	0 (0)
			Clinical	0 (0)	Retrospective	0 (0)	Quasi-Experimental	0 (0)
							Mixed/Qualitative	0 (0)
			Others	0 (0)	Others	2 (66.7)	Review	0 (0)
							Modelling Studies	2 (66.7)
	C2 Identification of Novel Strategies to Prevent and Manage Obesity.	15	Population	0 (0)	Cross-Sectional	5 (33.3)	Observational	3 (20.0)
							Cohort	0 (0)
			Community	11 (73.3)	Prospective	10 (66.7)	Single-Arm Interventional	3 (20.0)
							RCT	7 (46.7)
			Clinical	4 (26.7)	Retrospective	0 (0)	Quasi-Experimental	0 (0)
							Mixed	2 (13.3)
			Others	0 (0)	Other	0 (0)	Review	0 (0)
							Modelling Studies	0 (0)

Footnotes: Study Purpose and Scopes were referenced and adapted from Nutrition Research Priorities in Malaysia for 12th Malaysia Plan (Technical Working Group on Nutrition Research for National Coordinating Committee on Food and Nutrition) with omission of Scope A3 from the original document’(impact of early nutrition on development of adult obesity). 21 studies covered 2 scopes, 7 studies covered 3 scopes, numbers add up to more than N = 179.

BMI, Body Mass index; NCD, Noncommunicable Diseases; RCT, Randomized Controlled Trial; WC, Waist circumference; WHR, Waist-to-hip ratio; WHtR, Waist-to-height ratio.

#### Analysis of papers

The studies were scrutinized for their data source, study design, and key findings. We classified the data source as ‘population’ if the data was derived from population-based data such as quadrennial NHMS or Malaysian Adult Nutrition Survey (MANS), ‘community’ if it was done among specific segments of the population, or clinical if it was from a hospital or clinic-based study, or ‘others’ for modelling or review studies. We also classified the study type by study design whether observational or interventional, and by temporal nature.

#### Thematic analysis

Finally, using the key findings from the studies, we generated a narrative review. For clarity, we grouped the studies in the mapped scopes into five areas for thematic review synthesis:

1) Bio-psycho-social Impacts of Obesity
2) Predispositions for Obesity- Biological (sex, age, ethnicity and genetic) and Sociodemographic Factors
3) Dietary Drivers of Obesity- Composition, Patterns, and Food Policies
4) Physical Activity, Sedentary Behaviour and Lifestyle Interventions for Obesity
5) Clinical Interventions for Obesity Barriers and New Frontiers

### Findings

#### Overview of studies on obesity in Malaysia between 2015 and 2024

Our initial search returned 3626 articles. After removal of duplicates and exclusion of non-relevant or unavailable full texts, a total of 179 articles focusing on adult obesity in Malaysia were included and classified according to Purpose and Research Scopes (Fig. 1). To further support the discussions in the review, additional reports were included from various sources including government technical reports, relevant international reports, media reports and other Malaysian studies outside the prespecified review period (Supplementary Figure S1).

Table 1 summarizes the overall characteristics of the 179 studies and distribution of the studies across the different scopes, stratified by study designs, further illustrated in Supplementary Figure S2. 21 studies covered 2 scopes, while 7 studies covered 3 scopes. The outlines and key findings for each study for epidemiology studies, interventional studies, and developing new modalities (research purposes 1,2, and 3) are detailed in Supplementary Tables S2–S4 respectively.

Majority (69.8%) of the studies addressed obesity epidemiology (Purpose A), whereas only 8.9% of studies covered developing new modalities (Purpose C). Association of dietary intake, appetite control, eating behaviour and inflammatory status with obesity (Scope A4) had the most (n = 31) studies among all the scopes.

In terms of data source, the evidence base is dominated by community-sourced data (n = 122, 68.2%), with 20.1% of studies sourced from population-based data. There were 7 (3.9%) reviews, 10 (5.6%) modelling studies, and 5 (2.8%) qualitative studies overall. Among epidemiological studies (Purpose A, n = 113), majority (n = 96, 85.0%) were cross sectional. Shown in Table 1, studies assessing interventions (Scope B2, n = 23) comprised various designs including Randomized Controlled Trials (RCT) (43.5%), single-arm interventional studies (17.4%), and quasi-experimental studies, (4.3%). Studies assessing policy (Scope B3) comprised 15 reports, mainly from population-level (73.3%) cross-sectional studies (60.0%), but of a variety of observational (40.0%), qualitative (26.7%), and review (13.3%), one modelling study (6.7%) and RCT (6.7%).

##### Interpretation

The overall patterns in our mapping review closely resembles the earlier scoping review (2008–2017) by Mohamad Nor et al. in that Malaysian obesity research remains dominated by observational, cross-sectional, community-based studies.^7^ Although interventional studies have increased, in particular RCT's, from 3 (1.6%) to 25 (14.0%) in our current review, they were generally short (<1 year) in duration. Longitudinal cohorts and pragmatic interventions, i.e. real-world trials conducted under routine conditions rather than tightly controlled experimental settings, remain scarce, limiting causal inference and implementation guidance. Growth in modelling and analytic approaches reflects increasing methodological sophistication, but qualitative and mixed-methods research remain underrepresented.

### Theme 1: Bio-psycho-social impacts of obesity

#### Biological impacts: evidence by BMI thresholds

Malaysian studies have examined the biological impact of excess adiposity on people with overweight/obesity (PwO) using different BMI thresholds and adiposity measures, with varying associations to abnormal body composition, metabolic complications, inflammatory markers, and mortality. Table 2 summarizes these associations, across different study populations.

**Table 2:** Anthropometry measurements, thresholds and associated complications, evidence from Malaysian studies (2016–2024).

Anthropometry measurement	Threshold	Associations	Evidence	First author, year data source (Size) Study design
BMI	<23 kg/m^2^	Metabolic complications (diabetes, dyslipidaemia, and hypertension)	Women with normal BMI but high %BF, termed “Normal-weight obese” had higher odds of abdominal obesity (aOR: 2.64, 95% CI: 1.73–4.04), hypertriglyceridemia (OR 2.51, 95% CI 1.47–4.29) and hypertension (aOR: 1.63, 95% CI 1.15–2.31)	Moy 2016^9^ Community (N = 1095) Cross-sectional
			Among Malays, prevalence of metabolic syndrome among those with BMI <25 kg/m^2^ (average 22.6 kg/m^2^) was 9.3 to 10.2%, depending on definition, largely driven by abdominal obesity.	Al-Mahmood, 2016^10^ Community (N = 226) Cross-sectional
	23–24.9 kg/m^2^	Metabolic complications (DM, dyslipidaemia, hypertension) and inflammation	BMI >23 kg/m^2^ increased odds of undiagnosed DM (aOR: 1.65, 95% CI: 1.31–2.07), high blood pressure (aOR: 3.08, 95% CI: 2.60–3.63), and hypercholesterolemia (aOR: 1.37, 95% CI: 1.22–1.53).	Koo, 2023^11^ Population (N = 14, 025) Cross-sectional
			91% of patients with T2D had BMI >23 kg/m^2^ BMI >23 kg/m^2^ did not increase risk of DM complications.	Shaharuddin, 2023^12^ Clinical (N = 82) Cross-sectional
			Improved BMI thresholds to predict hypertension: 23.00 kg/m^2^ for men (AUC: 0.78; 95% CI 0.69, 0.90, sensitivity: 100%, and specificity: 47.1%) 24.05 kg/m^2^ for women (AUC: 0.85; 95% CI 0.78–0.91, sensitivity: 84.2%, and specificity: 73.4%)	Ching, 2020^13^ Community (N = 273) Cross-sectional
			Overweight/obese had higher Mean platelet volume, and obese had higher SCD40L levels than both overweight and normal weight group, suggesting higher platelet activation and inflammation	Riyahi, 2018^14^ Community (N = 112) Cross-sectional
	25–29.9 kg/m^2^	Mortality, Metabolic complications (DM, dyslipidaemia and hypertension), and inflammation	Over a follow-up of 153,814 person-years, overweight (BMI 25–29.9 kg/m^2^) had lower mortality compared to normal to at-risk BMI (18.5–24.9 kg/m^2^) (aHR: 0.81; 95% CI 0.68, 0.94)	Kee, 2017^15^ Population (N = 32 839) Retrospective cohort observational
			Overweight/obesity contributed 7.0% (95% CI 4.1–10.2%) to the risk of cancer, second only to tobacco smoking at 14.3% (95% CI 9.9–17.3%)	Teh, 2021^15^ Population (N = 18 800) Retrospective cohort observational
			People with BMI >25 kg/m^2^ had higher risk of DM (aOR: 1.47, 95% CI: 1.23–1.75) and hypertension (aOR: 2.60, 95% CI: 2.20–3.07)	Chong, 2023^16^ Population (N = 9782) Cross-sectional
			BMI positively correlated weakly high-sensitivity C-RP (r = 0.38), systolic diastolic BP (r = 028 and 0.31, p < 0.050)	Daud, 2019^17^ Community (N = 82) Cross-sectional
			BMI was positively associated with TG [*B* = 17, SE = 5.13, p = 0.001], LDL (*B* = 15.5, SE = 6.69, p = 0.014), and negatively associated with TC (*B* = −14.9, SE = 6.45, p = 0.022). WC was positively associated with TG (*B* = 14.6, SE = 4.13, p = 0.001)	Nadeem, 2021^18^ Community (N = 193) Cross-sectional
	≥30 kg/m^2^	Mortality, metabolic complications (DM, dyslipidaemia and hypertension)	23.7% of Malaysian adults were obese and disease free. The probability of these population becoming unhealthy obesity increases by 4.4% with each passing year.	Singh, 2020^19^ Population (N = 741) Cross-sectional
			BMI beyond this threshold increased odds of hypertension for men (aOR 5.29, 95% CI 0.94–29.73), but not for women (aOR 0.99, 95% CI 0.35–2.82)	Chua, 2017^20^ Community (N = 482) Cross-sectional
			BMI 30.0–34.9 kg/m^2^ was associated with lower all-cause mortality in never smokers (aHR 0.71; 95% CI 0.53–0.97) but higher CVD mortality in cohorts without diabetes, hypertension or hypercholesterolaemia. (aHR 4.51; 95% CI 1.03–19.71). BMI ≥35 kg/m^2^ conferred excess cardiovascular mortality for the overall population (aHR: 2.15, 95% CI: 1.11–4.15)	Kee, 2017^21^ Population (N = 32 839) Retrospective cohort observational
WHtR	0.50 (both sexes)	Unhealthy body composition	WHtR >0.5 was a robust threshold for identifying excess body fat [AUC: 0.66 (95% CI 0.59–0.74), sensitivity: 96.6%, specificity: 36.1%]	Low, 2022^22^ Community (N = 399) Cross-sectional
	0.54 (men) 0.53 (women)	Metabolic complications (Metabolic syndrome)	WHtR outperforms BMI to predict metabolic syndrome for men: WHtR of 0.54 (AUC: 0.83; 95% CI 0.74–0.91) vs BMI at 23.00 kg/m^2^ (AUC: 0.78; 95% CI 0.69,0.90) women: WHtR of 0.53 (AUC: 0.86, 95% CI 0.80–0.93) vs BMI at 24.05 kg/m^2^ (AUC: 0.85, 95% CI 0.78–0.91)	Ching, 2020^13^ Community (N = 273) Cross-sectional
	0.45 (men) 0.52 (women)	Metabolic complications (Hypertension)	WHtR was the best predictor of the presence of hypertension, in both men and women. WHtR >0.5 increased odds of hypertension for both men (aOR: 2.15, 95% CI: 1.16–4.01) and women (aOR 1.97, 95% CI 1.02–3.82)	Chua, 2017^20^ Community (N = 482) Cross-sectional
WC	92.5 cm (men) 85.5 cm (women)	Unhealthy body composition	WC cutoff points of 92.5 cm in men (AUC 0.93; 95% CI: 0.87–0.99; sensitivity: 87.9%; specificity 82.9%) and 85.5 cm in women (AUC 0.87, 95% CI 0.83–0.91; sensitivity 94.0% and specificity 55.4%) is the optimal number to detect abdominal obesity.	Ahmad, 2016^23^ Community (N = 669) Cross-sectional, observational
	90 cm (men) 80 cm (women)	Mortality	Over a follow-up of 31 668 person-years, having metabolic syndrome at baseline was associated with increased hazard of CVD mortality (aHR: 2.18, 95% CI 1.03–4.61), p = 0.041) and all-cause mortality (aHR: 1.47, 95% CI 1.00–2.14)	Kee 2021^24^ Population (N = 2525) Retrospective, longitudinal observational
	Not specified	Metabolic complications (Dyslipidemia)	WC was positively associated with TG (*B* = 14.6, SE = 4.13, p = 0.001)	Nadeem, 2021^18^ Community (N = 193) Cross-sectional
	Not specified	Inflammation	Among abdominally obese men (mean WC 103 ± 8.7 cm) and women (mean WC 94 ± 7.0 cm), there is positive correlation between WC and hs-CRP level (men: r = 0.36, p = 0.006; women: r = 0.18, p = 0.043)	Shahadan, 2018^25^ Community (N = 93) Cross-sectional
	85 cm (men) 76 cm (women)	Unhealthy body composition	WC reduction to 76 cm (women) and 85 cm (men) improved prediction of excess adiposity (AUC from 0.71, 95% CI 0.64–0.76 to 0.77, 95% CI 0.71–0.84]	Low, 2022^22^ Community (N = 399) Cross-sectional
	90 cm (men) 80 cm (women)	Metabolic complications	Abdominal obesity doubled the odds for hypertension for both men (aOR 2.66, 95% CI 1.05–6.73) and women (aOR: 2.09, 95% CI: 1.07–4.06)	Chua, 2017^20^ Community (N = 482) Cross-sectional
	90 cm (men) 80 cm (women)	Metabolic complications	Abdominal obesity increased odds of undiagnosed DM (aOR: 1.40, 95% CI: 1.17–1.67), high BP (aOR: 2.83, 95% CI: 2.45–3.26), and hypercholesterolemia (aOR: 1.26, 95% CI: 1.12–1.42).	Koo, 2023^11^ Population (N = 14, 025) Cross-sectional
WHR	>0.9 (men) 0.85 (women)	Metabolic complications (Hypertension)	High WHR was associated with increased odds of hypertension for women (aOR 1.99, 95% CI 1.04–3.80), but not men (aOR: 1.53, 95% CI 0.77–3.27)	Chua, 2017^20^ Community (N = 482) Cross-sectional
	Not specified	Metabolic complications (Dyslipidemia)	WHR was predictive of total cholesterol (*B* = 0.22, SE = 0.09; p = 0.010) and TG (*B* = 0.02, SE = 0.02, p = 0.002)	Nadeem, 2021^18^ Community (N = 193) Cross-sectional

Abbreviations: %BF, body fat percentage; 95% CI, 95% Confidence Interval; aHR, adjusted Hazards Ratio; aOR, adjusted odds ratio; AUC, area under receiver-operating characteristic curve; BMI, body mass index; BPL, blood pressure; CVD, cardiovascular disease; DM, diabetes mellitus; hs-C-RP, high-sensitivity C-reactive protein; LDL, low-density lipoprotein; SE, Standard Error; TC, total cholesterol; TG, triglyceride; WC, waist circumference; WHR, Waist-to-hip Ratio; WHtR, Waist-to-height ratio.

At BMI <23 kg/m^2^, individuals with high body fat already show higher odds of abdominal obesity, hypertension, hypertriglyceridemia,^9^ and substantial rates of metabolic syndrome.^10^

From 23 to 24.9 kg/m^2^, overweight status is associated with undiagnosed diabetes, hypertension, and hypercholesterolemia,^11^^,^^16^ with refined thresholds to improve prediction of hypertension among men at BMI of 23.00 kg/m^2^ and 24.05 kg/m^2^ for women.^13^ A clinic-based study showed most patients with diabetes already had BMI exceeding this threshold, while BMI did not exacerbate the risk of diabetes-related complications.^12^ Inflammatory markers such as Mean Platelet Volume (MPV) was higher among people with BMI >23 kg/m^2^ compared to normal weight individuals.^14^

BMI 25–29.9 kg/m^2^ was paradoxically associated with reduced all-cause mortality compared to BMI 18.5–24.9 kg/m^2^,^21^ though overweight/obesity at this threshold contributed 7% of cancer burden nationally.^15^ Associations with diabetes,^16^ hypertension^16^^,^^17^ and inflammation,^25^ were also established at this BMI range.

Above BMI of 30 kg/m^2^, majority (76.3%) of Malaysian PwO already have at least one metabolic complication, and younger PwO are more likely to be obese but disease-free compared to those aged above 65.^19^ BMI 30.0–34.9 kg/m^2^ was associated with lower all-cause mortality in never smokers but higher cardiovascular mortality in healthy cohorts without diabetes, hypertension or hypercholesterolaemia. Finally, BMI ≥35 kg/m^2^ conferred excess cardiovascular mortality for the overall population.^21^

Notably, waist-to-height (WHtR) ratio >∼0.5 consistently outperformed BMI in predicting excess adiposity and hypertension.^13^^,^^20^^,^^22^ WC and Waist-to-Hip Ratio (WHR) linked to excess adiposity, dyslipidaemia and hypertension.^18^^,^^20^^,^^21^^,^^23^ In a longitudinal study, metabolic syndrome, incorporating abdominal obesity, doubled all-cause mortality risk over 13 years.^24^

#### Psycho-social impacts

One study in northern Peninsula Malysia reported 10–20% of PwO with chronic medical illness had mental health issues, especially among the young, women, and the socioeconomically disadvantaged.^26^ Another study highlighted sex-specific differences in psychological factors driving obesity, whereby in men, perceived stress, suicidal ideation, and physical-health quality of life inversely predicted BMI, while in women, stressful life events increased and psychological QoL decreased BMI.^27^ Among public healthcare workers, depression doubled the odds of obesity.^28^ An mHealth intervention study among postpartum women, 60.8% of which were overweight/obese, reported 34.7% were at increased risk of depression and 25.0% were likely depressed.^29^ Studies during the COVID-19 pandemic also highlighted that links between obesity and depression.^30^^,^^31^ In an online survey during the COVID-19 lockdown period, overweight/obesity was associated with higher odds of mild-to-severe depression.^30^ Malaysians who gained weight during the pandemic reported higher depression scores, but this was not statistically significant after adjusting for sociodemographics and physical activity level.^31^

#### Economic impacts

Only one study based on data from 2011 estimated obesity-attributable burden of disease at 3239 person-years (men) and 2440 person-years (women) per 1000 persons.^32^ However, data on Disability-Adjusted Life Years for obesity in Malaysia are lacking, and data on Years of Life Lost estimates do not yet list obesity as a disease or estimate its economic costs.^33^ On an individual level, obesity did not directly affect wages but workers reported weight stigma in the job market.^34^ New modelling approaches have been proposed to quantify the productivity impact of obesity on the national economy.^35^

##### Interpretation

We grouped studies examining health impacts of obesity under this theme because it directly informs how obesity is defined in the Malaysian context. Local evidence demonstrates that metabolic risk emerges well below conventional BMI thresholds, evident among women with higher body fat and people with abdominal obesity.^9^^,^^10^ While the BMI-mortality curve resembles the commonly-cited obesity paradox,^36^ the current evidence consistently highlight that metabolic, inflammatory and cancer risks are already evident at BMI 23 kg/m^2^ and above. Therefore, we believe this reinforces BMI alone inadequately captures the health risks of excess adiposity. Abdominal measures such as WHtR >∼0.5 consistently provide stronger prediction of cardiometabolic disease. This is echoed by the recent nationally-representative Malaysian Ageing and Retirement Survey (MARS) among older Malaysians that found both BMI and Waist-Hip Ratio as independent risk factors for NCD's.^37^ Adoption of a dual measurement of both BMI and abdominal obesity to diagnose clinical obesity is a pragmatic, low-cost strategy to improve early detection, aligning with recent international recommendations.^4^^,^^38^

Evidence beyond conventional cardiometabolic disease is sparse. Data on sleep disorders, musculoskeletal disease, infertility, and other comorbidities are lacking. Evidence on psychosocial and economic impacts remain scarce, with only a handful of studies exploring mental health and stigma, and no high-quality data on productivity losses. Reports in other countries have estimated that direct medical costs of obesity range up to 17.8% of total health system expenditure and 2.24% of overall national gross domestic product (GDP), with indirect costs often outweighing direct costs.^39^

### Theme 2: Predispositions for obesity-biological (sex, age, ethnicity and genetic) and sociodemographic factors

#### Sex

Malaysian women generally have lower or similar rates of overweight, but consistently higher rates and risks of obesity compared to men.^3^^,^^16^^,^^27^^,^^40^, ^41^, ^42^, ^43^, ^44^, ^45^, ^46^ This echoes serial reports from serial NHMS and findings from analyses of earlier data from MANS (2003–2014) and a meta-analysis of 18 studies (2010–2016).^41^^,^^45^ Women had 33–64% times higher odds of obesity^16^^,^^27^^,^^40^^,^^46^ and displayed steeper BMI and waist circumference increases with age.^44^ Among women, higher body fat percentage (%BF) was linked to lower physical activity and unhealthy dietary patterns,^47^ while in postmenopausal women, skeletal muscle mass was weakly correlated with %BF.^48^

#### Age

Obesity prevalence peaked in middle-aged adults (30–59 years) and declined in older adults (>60 years)^16^^,^^46^ Data from NHMS 2015 indicated 30.7% of adults over 60 were overweight/obese, lower than the national average of 47.7%,^49^ a pattern concurred by two community studies.^42^^,^^46^ A prospective study among older Malaysians found weight gain increased fall risk [adjusted Risk Ratio (aRR) 2.86, 95% CI 1.02–8.02], but the association was attenuated by low baseline lean body mass in women, and strengthened when low muscle strength was additionally adjusted, whereas the effect was not seen in men.^50^

#### Ethnicity

Ethnic differences were consistently observed. Two separate analyses, one using the NHMS 2019 data^16^ and another focused on older Malaysians,^46^ observed that Malay, Indian, and other Bumiputera groups had consistently higher odds of overweight/obesity compared with Chinese. Longitudinally Malaysian Chinese appeared to experience smaller BMI and WC increases across the lifespan.^44^ Socioeconomic and behavioural factors contribute to interethnic difference for metabolic syndrome, with ethnic-specific analyses showing that rapid eating appeared to increase risk among Malays, low physical activity, and older age seemed to elevate risk among Chinese, while higher education level appeared protective among Malays.^51^ Vitamin D deficiency was higher among Indians and Malays than the Chinese, and levels were inversely and weakly correlated with BMI and body fat, with ethnicity emerging as a key determinant.^52^ Among indigenous groups or *Orang Asli*, patterns varied. In East Malaysia, obesity prevalence appeared similar between sexes and across communities, though cardiovascular risk factors differed.^53^ Up to one-third had metabolic syndrome, with central obesity being a major risk factor.^54^ However, compared to Malays, lower rates of abdominal obesity and metabolic syndrome were observed among certain *Orang Asli*.^55^

#### Genetic

A scoping review identified ten obesity-related genetic variants among Malaysians, namely: *LEPR*, *LEP*, *ADIPOQ*, *UCP2*, *ADRB3*, *MC3R*, *PPARγ*, *IL1RA*, *NFKB1*, and *FADS1*.^56^ The *NFKB1* (rs28362491) Ins/Ins genotype in combination with *HIF1* (rs11549465, CC) predicted greater obesity risk,^57^ whereas the *ACE II* with *ADRA2A* (rs553668, GG) appeared protective against central adiposity.^58^ Previously known obesogenic variants such as *IRX3* (rs3751723) and *FASN* (rs4246445, rs2229425, rs2228305, and rs2229422) single nucleotide polymorphisms (SNPs) were not associated with obesity in Malaysians.^59^^,^^60^ *ADIPOQ* +45T > G (rs2241766) and +276G > T (rs1501299), *FTO* (rs9939609) and *RETN* (rs1862513) SNPs were linked with obesity and metabolic syndrome among indigenous groups.^61^^,^^62^ Two studies looked at leptin gene variants but did not find polymorphisms significantly linked with obesity, although gender and interethnic differences were observed.^63^^,^^64^ Dopamine receptor (*DRD2*) SNPs were associated with emotional eating.^65^^,^^66^ while fatty acid translocase gene *CD36* variants influenced oral fat perception.^67^ but direct links with obesity was not reported. Meanwhile, gene–diet interactions may contribute to differing cardiovascular risks among Malaysian PwO.^68^, ^69^, ^70^, ^71^

#### Sociodemographic and cultural determinants

National trend analyses from 1996 to 2011 showed shift of obesity burden to the socioeconomically disadvantaged, with distinct trends at different regions of Malaysia. In Peninsular Malaysia, female overweight/obesity and abdominal obesity are increasingly concentrated among the poor. In contrast, in East Malaysia, rates of obesity in women showed no clear socioeconomic gradient, and distributions of male obesity remained concentrated among the rich.^72^ Spatial-clustering analyses identified suburban areas as obesogenic hotspots whilst urban areas had lower rates.^73^ Separately, obesity was reported to be less prevalent among higher-educated Malaysians.^74^ While socioeconomic disparities in accessibility to healthy diets and environments exist, there may be some commonalities in terms of cultural norms that drives obesity in the Malaysian population, as described in a series of qualitative studies.^75^^,^^76^ Common themes among Malaysian PwO include a food-centric culture, social norms and distorted perceptions on obesity, and weight stigma.^77^ Health Belief Model analysis revealed that perceived severity of obesity and behavioural intention significantly predicted BMI, and these perceptions were shaped by demographic factors.^78^

#### Occupational and environmental exposures

Occupational risks were identified in several groups. Healthcare professionals (HCPs), particularly in the public sector, have increasing trends of obesity and abdominal obesity.^79^ Among them, nurses have particularly higher odds of being obese.^80^ Obesity among HCPs correlated with age and associated with restrained eating behaviours,^81^ and longer years of service (>5 years).^28^ In manufacturing workers, night-shift work doubled the risk of metabolic syndrome.^82^ Environmental studies reported that air pollutant moderately correlated with higher appetite.^83^

#### Novel methods in risk profiling

Several research groups have applied novel methods to assess the epidemiology of obesity in Malaysia. Incorporating artificial intelligence techniques, Safaei and colleagues identified significant risk factors of obesity.,^84^ Wong and colleagues demonstrated that machine learning models could predict overweight/obesity with high accuracy using data from online workplace surveys.^85^ Two groups used Bayesian methods^86^ and geostatistical models,^73^ separately, to identify obesity distribution patterns across different geographical regions in Malaysia.

##### Interpretation

Understanding predisposition patterns for obesity is important for efficient resource allocation. Overall, the studies in this report show obesity risks and trajectories were consistently higher among women, with rates peaking in midlife, similar to the earlier review by Lim KG (2000–2015),^6^ and in tandem with trends in other middle-income countries.^87^ While lower rates were seen in older adults, it may reflect survivorship bias rather than true protection, with sarcopenic obesity posing unique risks. Ethnic differences seen locally are equally observed in neighbouring Singapore with a comparable ethnic composition, which shows risks similarly lowest among Chinese and higher among Malays and Indians.^88^ The reasons for these disparities are complex, and genetic,^89^ adiposity,^90^^,^^91^ hormonal,^92^ and sociodemographic^93^ factors have been implicated. Among indigenous groups, vulnerability tied to nutrition transition is heterogeneous. Local obesity-genetic studies remain largely exploratory, with limited immediate real-world applicability. In contrast, evidence on occupational risks highlights more actionable opportunities for targeting vulnerable groups through tailored interventions. Nevertheless, the approaches that highlighted harnessing AI and big-data modelling offer promise for precision risk-profiling and managements. Qualitative studies reveal cultural norms, distorted weight perceptions, and stigma that can reinforce obesogenic behaviours, and will require validation across the wider Malaysian population.

Transitions in social determinants, such as urbanization and changes in income and education levels, have contributed to shifts in nutrition patterns and rising obesity across Asia.^94^ Jaacks et al. conceptualized a model of country-level obesity burden shifts from higher-income, urban groups (Stage 1) to becoming concentrated among lower-income populations with widening disparities (Stage 2–3).^95^ Macroscopically, the Malaysian pattern aligns with early Stage 3, with obesity increasingly entrenched in lower socioeconomic groups and among women, while national prevalence continues to rise. This trajectory mirrors that of some high-income countries, though Malaysia remains distinct in facing a concurrent double burden of both obesity and undernutrition.^43^^,^^96^ Malaysia is at a critical inflection point–without effective structural and behavioural interventions, it risks consolidating a Stage 3 obesity profile, with deepening disparities and escalating metabolic burden.

### Theme 3: Dietary drivers of obesity: composition, patterns, and food policies

#### Diet composition

Diet studies and reports on food environment policies (Scope 1D and 2C) comprised cross-sectional, longitudinal-observational, RCT's, modelling, and review studies. A quasi-historical analysis of Food Balance Sheets and health surveys linked Malaysia's obesity rise to a 30% calorie oversupply, with higher wheat, sugar, meat, seafood, and eggs, and a shift from plant-to animal-based protein.^97^ A scoping review confirmed poor adherence to healthy eating guidelines across all age groups, with deficits in fruits, vegetables, legumes, and dairy and excess of meat, salt, and sugar,^98^ while another complementary review on youth obesity suggested current research focused more on intake patterns rather than deeper behavioural drivers.^99^ Across multiple studies, excess protein, meat, sugar, sodium, and ultra-processed food intake, combined with inadequate plant, dairy, and fibre consumption were consistently linked to higher obesity risk.^100^, ^101^, ^102^, ^103^, ^104^ A community-based study recommended intake of three servings of legumes daily among PwO to reduce cardiovascular risk.^105^ Other studies highlighted cardiometabolic effects, including worsening insulin resistance and lipid profiles from high-fat, high-carbohydrate diets,^106^ and adverse insulin secretion when saturated fats were replaced by carbohydrates but not by monounsaturated fats.^107^ In terms of micronutrients, low selenium and high copper intake were associated with insulin resistance.^108^ An RCT applied an individualized high-protein, energy-restricted, high-vitamin E and high-fibre (Hipcref) reported modest weight reduction, along with improvements in body composition, insulin resistance and inflammatory markers.^109^

#### Dietary patterns

The decade-long nationwide Malaysian Adult Nutrition Surveys (MANS) classified three dietary patterns, but none consistently predict risk of overweight/obesity, suggesting shifting habits.^110^ Conversely, a recent urban study showed a ‘Western’ diet (high in carbs, animal-based food, sugar, and fast food) raised obesity risk while a ‘Prudent’ diet rich in vegetables, fruits, legumes, and pulses was protective in men.^111^ Behavioural aspects also emerged, as the Malaysian Food Barometer found discordance in reported and actual food intake linked to obesity,^74^ while community studies similarly found low levels of intuitive eating, higher hedonic hunger and cognitive distortions predicted adiposity.^112^^,^^113^ Studies from COVID-19 provide insight on eating behaviour and weight changes, albeit with mixed results with some reporting increase in healthier food choices and eating habits^114^ associated with weight loss,^115^^,^^116^ whereas those who reported weight gain had poor self-regulation in eating^117^, ^118^, ^119^, ^120^ or food choices.^115^^,^^121^ Chrononutrition, linking meal timing, circadian rhythm, and metabolic health is an emerging area.^122^, ^123^, ^124^, ^125^, ^126^ Late-day energy and carb intake increased risk of being metabolic unhealthy among PwO,^123^ supported by an intervention that reported shifting majority daily protein intake to the morning improved odds of transitioning to healthier profiles.^124^ Conversely, another observational study among women reported that skipping breakfast and eating the largest meal at night was associated with being underweight.^125^

#### Food environment policy

While policies exist, a mixed-methods assessment found that their implementation and enforcement remain weak,^127^ with responsibility shared between regulators^128^ and industry.^129^ Dietary public health messaging in Malaysia has improved but needs tailoring to diverse social, cultural, and literacy contexts.^130^ The Malaysian Healthy Plate (MHP) concept, introduced in 2016 for a simple public health messaging on healthy food options, has seen limited reach four years after its implementation as most (79.6%) Malaysians remain unaware of it.^131^ Awareness of the MHP was associated with adequate fruit and vegetable intake,^132^ whereas another RCT showed that the MHP concept was only effective when practiced with intermittent fasting.^133^ Nevertheless, studies suggest awareness alone rarely translates to healthier food purchase. Socioeconomic constraints further shape diets as healthy diets are costly in Malaysia.^134^ Food insecurity among rural Malay and Indian women was linked with higher rates of obesity.^135^ Majority of the bottom 40% (B40) of the Malaysian economic spectrum consume inadequate fruits and vegetables and excessive sugar-sweetened beverages (SSB).^136^ Community surveys in low-income communities reported preference for interventions such as reduction of fruit and vegetable prices over evidence-based strategies like changes in product placement and nutrition labelling.^137^ Lastly, even health-conscious consumers display complex motivations in food purchasing.^138^

##### Interpretation

The mapped evidence overall suggests Malaysia's nutritional transition over the past few decades is characterized by three key factors: caloric oversupply from high availability energy-dense foods, environmental inequity for access to healthy foods, and behavioural maladaptation in eating habits and food options. These drivers closely interlink to perpetuate the obesogenic dietary environment: caloric oversupply sets the scene for overconsumption, inequity in healthy food access drives poor dietary composition, and behavioural maladaptation compound the effect. The complexity of influences in food environment is reflected in another recent population-based spatial analysis that showed proximity to fast-food outlets was associated with higher BMI in men but not women, underscoring the presence of multiple determinants of obesity beyond diet alone.^139^

While sustaining a caloric deficit remains the cornerstone of dietary interventions for weight loss, coupling this with balanced nutritional composition can deliver health benefits beyond weight reduction, as illustrated by the Hipcref diet trial.^109^ The greater challenge lies in translating such individualized, resource-intensive interventions into scalable public health strategies. Even the Malaysian Healthy Plate, a simple, nationally endorsed messaging tool, has achieved limited uptake, reflecting the difficulty of achieving population-level change.^131^^,^^133^ Together, these patterns indicate that poor eating habits and disrupted circadian eating rhythms amplify the impact of caloric oversupply and poor nutrition composition.

### Theme 4: physical activity, sedentary behaviour, and lifestyle interventions for obesity

#### Physical activity and sedentary behaviour

Most Malaysian studies assessed self-reported physical activity (PA) based on the International Physical Activity Questionnaire (IPAQ) or its validated variants. In accordance with the standard IPAQ and WHO guidelines, PA levels are generally classified as low [<600 Metabolic Equivalent of Task (MET)-minutes/week], moderate (600–2999 MET-minutes/week), and high (≥3000 MET-minutes/week) based on total energy expenditure from walking, moderate, and vigorous activities. Clear sex differences were observed: with an analysis of NHMS 2019 data showing while majority (69.0%) of Malaysians reporting at least moderate PA levels, men were more likely to have high PA and higher total and vigorous PA than women, while low PA was associated with overweight/obesity among men but not women.^140^ Young sedentary men with high BMI performed worse in fitness tests.^141^ A study among urban low-income housewives with obesity, majority (90.5%) had low physical activity levels.^142^ Among rural communities in East Malaysia, half were moderately active, with women more active in domestic domains and men in work and leisure-time domains.^143^ Sociodemographic factors also mediated PA levels, as a population-based study reported age affected PA levels among PwO of different severity, and PwO with higher income engaged less in moderate-to-vigorous activity compared to lower income peers.^144^ Locality context matters, as Malaysians living in high-walkability neighbourhoods had higher accelerometer-measured PA,^145^ while a nationwide survey showed domestic activities dominated overall PA and inactivity was more common in rural than urban areas.^146^ Notably, discrepancies emerged between self-reported and objectively measured PA, with only accelerometer-based activity correlating with lower BMI.^147^^,^^148^ The COVID-19 travel restrictions amplified sedentary behavior, leading to consistent reports of modest weight gain across several populations.^31^^,^^116^^,^^118^^,^^149^^,^^150^ Clinically, low PA combined with energy-dense diets were linked to nonalcoholic fatty liver disease in centrally obese patients with diabetes.^151^ A modelling analysis derived from population data concluded that a vigorous level of physical activity of minimum 1500 MET-minutes per week is required for significant weight loss, setting a bar for future recommendations.^152^

#### Lifestyle interventions for obesity

We reviewed 22 studies which investigated various models of community-based lifestyle interventions for obesity, summarized in Table 3. Eight studies examined PA interventions only, two studies examined dietary interventions only and 13 studies examined a combination of both, whereas three studies had unconventional interventions-psychological approach, educational handouts and nutrition education in food handlers. Majority (n = 20, 90.9%) were micro-level interventions which target individual behaviour or clinical management, and only two (0.09%) were meso-level interventions which act on community, organisational, or environmental settings, whereas macro-level interventions which address systemic or policy determinants across society were not found. Durations of studies ranged from six weeks to one year, with most lasting six months. Studies focusing on diet-only interventions^109^^,^^133^ were reported in Theme 3.

**Table 3:** Community-based lifestyle interventions for obesity in Malaysia from 2016 to 2024.

Author/Title	Population/Sample	Level, type and duration of intervention	Intervention	Control	Key findings
Abdullah et al.^133^ The Effect of a Combined Intermittent Fasting Healthy Plate Intervention on Anthropometric Outcomes and Body Composition Among Adults with Overweight and Obesity: Nonrandomized Controlled Trial	Aged 19–59 years old with BMI ≥23 kg/m^2^ N = 177	Micro Diet 6 months	Combined intermittent fasting and healthy plate (IFHP) group: observe dry fasting from dawn to dusk for 2 days a week and practice HP for the rest of the week (n = 91)	Healthy plate (HP) group: division of plate portions into a quarter for protein, a quarter for complex carbohydrates, and a half for fruits and vegetables for at least 1 main meal per day (n = 86)	**Change in anthropometry and body composition** Weight: −1.81% (p = 0.003) BMI: −1.76% (p = 0.005) WC: −1.75ˆ% (p = 0.001) %BF: −4.01% (p < 0.001) Visceral fat area: −4.78% (p < 0.001) Skeletal muscle mass: +0.01% (p > 0.999) **Other findings:** HP group showed significant reduction in HC (−1.034 cm; p = 0.030) but no significant reduction in weight, BMI, %BF, body fat mass, and visceral fat area, and no significant increase in skeletal muscle mass.
Mitra et al.^109^ Effect of an individualised high-protein, energy-restricted diet on anthropometric and cardio–metabolic parameters in overweight and obese Malaysian adults: a 6-month randomised controlled study	Aged >18 years old with BMI ≥23 kg/m^2^ N = 128	Micro Diet 6 months	Hipcref (high-protein, energy-restricted, high-vitamin E and high-fibre) diet charts based on their personal preferences (n = 65)	Generalised dietary advice based on Malaysian Dietary Guidelines 2010 (n = 63)	**Change in anthropometry and body composition** Weight: −4.58% (p < 0.001) BMI: −4.73% (p < 0.001) WC: −6.65% (p < 0.001) %BF: −5.60% (p < 0.001) **Other findings:** Control group had significant increase in weight (+1.76%, p = 0.012) and BMI (+2.04%, p = 0.007) from baseline to month six Intervention group showed 33.3% (p < 0.001) reduction in HOMA-IR and 23.0% (p = 0.020) reduction in hsCRP
Saad et al.^153^ The Effects of a 7000-Step Goal and Weekly Group Walking Program for Overweight and Obese Elderly People in Sarawak, Malaysia: A Quasi-experimental Study	Older adults aged 60 years–75 years old with BMI >24.9 kg/m^2^ N = 109	Micro Physical activity 12 weeks	Combination of gradual walking program that used pedometer feedback as a motivation tool and weekly group activities (n = 48)	No interventions (n = 61)	**Change in anthropometry and body composition** Weight: −3.25% (p < 0.001) BMI: −3.27% (p < 0.001) WC: −3.13% (p < 0.001) Visceral Fat %: −9.58% (p < 0.050) Skeletal Muscle %: +6.68% (p < 0.001)
Kok et al.^154^ A Pedometer-based Intervention with Daily Walking Steps and its Relationship with Nutritional Status among Overweight/Obese University Students in Kuala Terengganu	University students in Terengganu BMI >25 kg/m^2^ N = 23	Micro Physical activity 8 weeks	Participants needed to reach the targeted steps according to the plan of the intervention phases where each phase lasted for 2 weeks. The intervention for was modified to participants’ baseline step count and increased by 10% every 2 weeks (n = 8)	No interventions (n = 15)	**Change in anthropometry and body composition** Weight: not reported BMI: −1.55% (p = 0.017) WC: 3.85% (p = 0.080)
Nik Yahya et al.^155^ A feasibility of simulation-based exercise programme for overweight adult in higher learning institutions	Students in two higher learning institutions in Kuantan, Pahang, Aged >18 BMI ≥23 kg/m^2^ N = 52	Micro Physical activity 10 weeks	A booklet and CD to do aerobic and resistance exercise for a minimum of 25 min per day, 3 times a week (n = 27)	No interventions (n = 25)	**Change in anthropometry and body composition** Weight: not reported BMI: −2.68% (p < 0.001) WC: −1.38% (p = 0.001) %BF: −2.96% (p = 0.001)
Yaacob et al.^156^ Dumbbells and ankle-wrist weight training leads to changes in body composition and anthropometric parameters with potential cardiovascular disease risk reduction	Aged 18–60 years old with BMI ≥23 kg/m^2^ N = 89	Micro Physical activity 6 months	Participants in the dumbbell (DB) group performed structured group exercises 3 times per week using a pair of 1 kg dumbbells (n = 40) Participants in the ankle-wrist weight (AW) group were given one pair of 500g ankle weights and one pair of 500g wrist weights to be worn during the activities of daily living three days per week for at least 20 min (n = 49)	–	**Change in anthropometry and body composition** Weight: not reported BMI: −0.20% (AW), −0.60% (DB) (p = 0.662) WC: −3.82% (AW), −4.44% (DB) (p < 0.001) %BF: −2.95% (AW) −3.23% (DB) (p < 0.001) Skeletal Muscle %: +3.12% (AW) 3.30% (DB) (p < 0.001) **Other findings:** Intervention group had significant reduction in BMI only at week six (−0.23 kg/m^2^; p = 0.007).
Heng et al.^157^ The Feasibility of high-intensity interval training (HIIT) In The Workplace Obesity Management Program	Employees in a research institute aged 25–59 years with BMI ≥23 kg/m^2^ in Kuala Lumpur N = 108	Micro Physical activity 10 weeks	HIIT training 3 times a week, 30 min each	–	**Change in anthropometry and body composition** Weight: −2.38% (p < 0.001) BMI: −2.95% (p < 0.001) WC: −0.01% (p = 0.905) **Other findings:** Reduction in total cholesterol (−3.10%, p = 0.039)
Chen et al.^158^ Effects of Brisk Walking on Plasma Lipoprotein(a), Total Antioxidant Status, Aerobic Fitness, Percent Body Fat, Waist Circumference and Resting Blood Pressure in Overweight and Obese Females	Female participants from Universiti Sains Malaysia Aged 20–35 years old with BMI 23.0–40.0 kg/m^2^ N = 38	Micro Physical activity 6 weeks	Brisk walking exercise conducted 3 times per week for 6 weeks (n = 19)	Refrain from any other exercise programme and continued their normal diet (n = 19)	**Change in anthropometry and body composition** Weight: not reported BMI: not reported WC: −3.48% (p < 0.001) %BF: −3.71% (p < 0.001) **Other findings:** Both groups had significantly reduced plasma lipoprotein(a) level (p < 0.001). Intervention group also had significant reductions in resting blood pressure (p < 0.001). Both groups had no significant differences in total antioxidant status and aerobic fitness after the brisk walking programme.
Cheah et al.^159^ A Multicomponent Workplace Environmental Intervention to Promote Physical Activity among the Staff of Universiti Malaysia Sarawak	University employees aged between 20 and 60 years old N = 148	Micro Physical activity 6 months	Physical activity support and organizational support components (n-75)	No interventions (n-73)	Weight and anthropometry changes not measured **Other findings:** The intervention group showed significant improvements in total physical activity self-regulation (PASR) (η_p_^2^ = 0.021), goal setting (η_p_^2^ = 0.024), total MET-min/week (η_p_^2^ = 0.031), housework-related physical activity (η_p_^2^ = 0.101), and step/week (η_p_^2^ = 0.827)
Mohd Roslan et al.^160^ Effects of an 8-week community exercise program on health-related physical fitness in overweight and obese working adults	Working adults aged 18–50 years old with BMI 23–34.9 kg/m^2^ N = 185	Micro Physical activity 8 weeks	8-week exercise program for 60 min/day, 3 days/week (n = 90)	Briefing regarding physical fitness, health-related benefits and advised to exercise regularly for 8 weeks (n = 103)	Significant differences in the mean improvement between intervention and control group in cardiorespiratory fitness, muscle strength, muscle endurance, flexibility, and the reduction in %BF (all p < 0.001). No significant increase in lean mass between groups (p = 0.218).
Fazliana et al.^161^ Effects of weight loss intervention on body composition and blood pressure among overweight and obese women: findings from the MyBFF@home study	Housewives aged 18–59 years old with BMI 25.0–39.9 kg/m^2^ in Kuala Lumpur N = 328	Micro Diet and physical activity 12 months	Individual diet counselling, group exercise and self-monitoring tools (food diary and physical activity diary) for 6 months, then followed-up for another 6 months without any intervention as part of maintenance period (n = 169)	A women's health seminar package (n = 159)	**Change in anthropometry and body composition** At 6 months: Weight: −1.49% (p < 0.001) BMI: −1.55% (p < 0.001) WC: −3.16% (p < 0.001) %BF: −3.15% (p < 0.001) Visceral fat area: −3.47% (p < 0.001) Body fat mass: −3.46% (p < 0.001) Skeletal muscle mass: +0.22% (p = 0.646) At 12 months: Weight: −0.66% (p < 0.001) BMI: −0.64% (p < 0.001) WC: −4.32% (p < 0.001) %BF: −1.55% (p = 0.152) Visceral fat area: −2.58% (p = 0.004) Body fat mass: −2.59% (p = 0.002) Skeletal muscle mass: +0.90% (p = 0.418) **Other findings:** At six months, both intervention and control groups also reported significant reductions systolic blood pressure (−6.81, 95% CI −9.72, −3.90 mmHg vs −7.95, 95% CI −11.69, −4.20 mmHg, both p < 0.001; between group difference p = 0.629), which were not different between two groups Intervention group showed a consistent decreasing trend of pain score mainly for back pain. Higher BMI was significantly associated with pain score. At six months, both intervention and control groups had significantly reduced total cholesterol (−0.27, 95% CI −0.42, −0.11 mmol/L, p = 0.001 and −0.21, 95% CI −0.38, −0.05, p = 0.012, between group difference −0.06, 95% CI −0.28, 0.17, p = 0.620) but only intervention group retained the reduction in maintenance phase (−0.05, 95% CI −0.19, 0.09 mmol/L, p = 0.488) while the level increased significantly in the control group (+0.22, 95% CI: 0.06,0.38 mmol/L, p = 0.008) Intervention group showed a trend of reduction in Fasting Plasma Glucose (−0.11, 95% CI −0.32, 0.10 mmol/L, p = 0.287) at 6 months and significantly decreased at 12 months (−0.19 95% CI: –0.32, −0.06 mmol/L, p = 0.005). Significant improvement in health-related quality of life (HRQOL) (59.82 ± 26.60 to 66.13 ± 22.82, p < 0.001) at 6 months. No significant association between weight reduction and HRQOL improvement.
Kassim et al.^162^ Effects of Lifestyle Intervention towards Obesity and Blood Pressure among Housewives in Klang Valley: A Quasi-Experimental Study (MyBFF@Home)	As above	As above	As above	As above	
Mohd Zaki et al.^163^ Impact of community lifestyle intervention on anthropometric parameters and body composition among overweight and obese women: findings from the MyBFF@home study	As above	As above	As above	As above	
Mohd Sallehuddin et al.^164^ Changes in body pain among overweight and obese housewives living in Klang Valley, Malaysia: findings from the MyBFF@home study	As above	As above	As above	As above	
Ahmad Zamri et al.^165^ Effectiveness of a community-based intervention for weight loss on cardiometabolic risk factors among overweight and obese women in a low socio-economic urban community: findings of the MyBFF@home	As above	As above	As above	As above	
Ahmad Zamri et al.^166^ Weight Change and Its Association with Cardiometabolic Risk Markers in Overweight and Obese Women	As above	As above	As above	As above	
Ambak et al.^167^ The effect of weight loss intervention programme on health-related quality of life among low income overweight and obese housewives in the MyBFF@home study	As above	As above	As above	Only intervention group was analysed.	
Abdul Aziz et al.^168^ Influence of co-morbidity on body composition changes after weight loss intervention among overweight housewives: a follow-up study of the MyBFF@home	Control group in the My Body is Fit and Fabulous at Home—without comorbidities (MyBFF@home) Phase II (G1). (n = 84) Housewives with obesity and co-morbidities (G2). (n = 42) N = 126	Micro Diet and physical activity 6 months	Both groups received dietary counselling, physical activity (PA) and self-monitoring tools.	–	Obesity without co-morbidities group (G1) showed a larger decrease in mean visceral fat area compared to the obesity with co-morbidities group (G2) (between group difference: −11.49, 95% CI − 20.07, −2.91 cm^2^ p = 0.009).
Tan S-T et al.^169^ Effectiveness of an integrated-weight management programme (i-WMP) in reducing body weight among noncommunicable disease patients in Malaysian government primary care clinics: a randomised controlled trial	Patients attending primary health clinics with NCD aged ≥18 and BMI ≥25 kg/m^2^ in Selangor N = 244	Micro Diet and physical activity 4 weeks	Group-based intervention developed based on behavior change wheel and operationalization of behaviour change techniques delivered over 3 sessions (n = 122)	Standard of care (n = 122)	Absolute changes in anthropometry and body composition not reported. Other findings: Based on Generalized Linear Mix Model (GLMM) analysis, the intervention was significantly effective in reducing weight F (2,499) = 16.020; p < 0.001, BMI, F (2, 499) = 16.711, p < 0.001; WC (F (2, 499) = 16.767, p < 0.001, and improving various diet and physical activity measures. Only %BF and MET-minutes/week were not significantly improved.
Kong et al.^170^ Worksite weight management program: A three-months intervention study in a primary health care setting	Employees from Bintulu government polyclinic aged 18–59 years with BMI ≥18.5 kg/m^2^ in Sarawak N = 88	Micro Diet and physical activity 12 weeks	Modification of dietary intake by community registered dietitians and increasing high-intensity interval training (HIIT) with motivational interviewing (n = 43)	Traditional counselling and weekly aerobic exercise from medical officer and physiotherapist. (n = 45)	**Change in anthropometry and body composition** Weight: −6.80% (p = 0.010) BMI: not reported WC: −8.37% (p = 0.010) HC: −7.05% (p = 0.010) **Other findings:** Intervention group demonstrated a significant improvement in all cardiometabolic risk factors [total cholesterol (−22.7%, p = 0.010), LDL (−30.5%, p = 0.01), HDL (+20.8%, p = 0.010), fasting blood sugar (−4.9%, p = 0.040), 2-h oral glucose tolerance test glucose (−8.3%, p = 0.010), HbA1c (−4.8%, p = 0.010)].
Rusali et al.^171^ Comparison of the Effectiveness of Online and Face-to-Face Weight-loss Interventions in the Workplace: Evidence from Malaysia	Adults aged 20–59 years old with BMI ≥24.9 kg/m^2^ in Klang Valley N = 108	Micro Diet and physical activity 4 months	Face-to-face intervention (FT): Structured weight reduction program called Slim Shape Module™ consisting of 16 2-h sessions (32 h) of talks, demonstrations, interactive activities, hands–on activities and exercise sessions (n = 38) Online intervention (OG) Diet, behavioural and physical activity delivered via an online weight education program (n = 31)	Printed booklets with educational information about diet, physical activity, and behaviour modification for weight reduction given during the first meeting (n = 39)	**Change in anthropometry and body composition** Weight: −1.39% (OG) (p = 0.027), −7.08% (FT) (p = 0.001) BMI: −1.24% (OG), −7.9% (FT) (p = 0.002) WC: −4.36% (OG), −8.03% (FT) (p = 0.194) %BF: −1.30% (OG), −6.47% (FT) p ≤ 0.001 **Other findings:** FT group had significantly greater reduction in triglycerides (−35%), total cholesterol to HDL-C ratio (−7.1%), energy intake (−703 kcal/day), carbohydrates (−38.7%) and fat intake (−41.2%) and significant increase in HDL-C (+7.7%) as compared with OG and control group. (All p < 0.050)
Jamal et al.^172^ Effectiveness of a Group Support Lifestyle Modification (GSLiM) Programme among Obese Adults in Workplace: A Randomised Controlled Trial	Employees from a local university with BMI >27.5 kg/m^2^ in Kuala Lumpur N = 194	Micro Diet and physical activity 24 weeks	Group Support Lifestyle Modification (GSLiM) activities including self-monitoring, cognitive-behaviour sessions, exercise as well as dietary change advocacy, which were conducted through seminars and group sessions (n = 97)	Dietary counselling (n = 97)	**Change in anthropometry and body composition** Weight: −2.94% (p < 0.001) BMI: −2.96% (p < 0.001) WC: −1.58% (p < 0.001) **Other findings:** 19.6% of the participants in the GSLiM group achieved 6% weight loss compared to 4.1% in the comparison group (risk ratio 4.750; 95% CI: 1.680–13.450).
Mohamed et al.^173^ Benefits of Community Gardening Activity in Obesity Intervention: Findings from F.E.A.T. Programme	Aged between 25 and 59 years old with BMI >23 kg/m^2^ N = 61	Micro Diet and physical activity 12 weeks	Fit, Eat, Active and Training (F.E.A.T) programme: focusing on nutrition education, interactive and informative education sessions and participation in physical activity and exercise training, featuring community gardening as a medium (n = 31)	No intervention (n = 30)	**Change in anthropometry and body composition** Weight: −3.50% (p < 0.050) BMI: −4.10% (p < 0.050) WC: not reported **Other findings:** Intervention group had significantly greater vegetable intake (1.8 ± 0.7 serving size) as compared to control group (0.7 ± 0.5 serving size) (p < 0.050)

Saparuddin et al.^174^ Metabolite, Biochemical, and Dietary Intake Alterations Associated with Lifestyle Interventions in Obese and Overweight Malaysian Women	Women with metabolically healthy overweight/obesity (MHO) (n = 36) Women with metabolically unhealthy overweight/obesity (MUO). (n = 34) Aged 18–59 years old, BMI ≥25.0–≤39.9 kg/m^2^ in Klang Valley N = 70	Micro Diet and physical activity	Both groups received intervention consisting of diet control, physical activity and self-monitoring tools (pedometer, physical activity, and diet diaries)	–	6 months	**Change in anthropometry and body composition** MUO Group: Weight: −1.30%, p = 0.015 BMI: −1.17% (p = 0.023) WC: −2.48%, (p < 0.001) **Other findings:** There were no significant improvements in MHO group. There was a significant decreasing trend in serum trimethylamine-N-oxide, arginine, ribose, aspartate, carnitine, choline, and tyrosine in the MHO group
Mazri et al.^124^ Early-day Protein Intake Influences the Transition of Metabolic Health Phenotypes Among Adults with Obesity	Adults aged 20–59 years with BMI >25 kg/m^2^ N = 91 Non-shift workers	Micro Diet and physical activity	Lifestyle intervention (physical activity) and diet: calorie prescriptions of 1600–1800 kcal/day for men and 1200–1500 kcal/day for women, with energy distribution adjusted to chronotype—morning types consuming more earlier in the day, and evening types later.	Usual habits	12 weeks	**Transition in phenotype between Metabolically Healthy Obesity to Metabolically Unhealthy Obesity or Metabolically Healthy Normal Weight** Post-intervention, 24% showed positive phenotype change (MUO→MHO or MHN). Positive responders had greater weight loss (−7.0% vs −3.2%, p = 0.022), fat loss (−3.4% vs −1.3%, p = 0.013), visceral fat loss (−1.3 vs −0.5, p = 0.030), higher protein intake (+5.2% vs +1.1%, p = 0.004) especially earlier in the day (+4.3% vs +1.1%, p = 0.029), and more morning chronotypes (72.7% vs 35.7%, p = 0.033).
Pitil et al.^175^ The impact of acceptance and commitment therapy (ACT) on the psychological defense mechanism and weight loss program: A randomized controlled trial among university students during COVID-19 movement control order	University students with BMI ≥23.0 kg/m^2^ who reported high immature and neurotic defense styles N = 152	Micro Diet, physical activity, and psychobehavioural therapy	ACT program designed to promote psychological flexibility that encourages openness, awareness, and engagement ACT Group: Six sessions of at least 1.5 h per session, once a week (n = 52) ACT-Exercise (ACT-Ex) group: In addition to the ACT sessions, an exercise program was conducted three times per week via a 1-h virtual session (n = 50)	No interventions (n = 50)	6 weeks	**Change in anthropometry and body composition** Weight: −4.55% (ACT), −7.79% (ACT-Ex), p = 0.001 BMI: −4.14% (ACT), −7.73% (ACT-Ex), p = 0.001 WC: not reported **Other findings:** Both interventions were also found to have improving psychological defense mechanism, including experiential avoidance.
Thamby et al.^176^ Effectiveness of an Interventional Educational Tool on Certain Overweight and Obesity Parameters Among the General Public in Sungai Petani, Kedah Darul Aman, Malaysia	Malaysian citizens residing in Sungai Petani for the last three generations, aged between 18 and 30 years old with BMI of >25.0 kg/m^2^ N = 551	Meso Health education	Pre-validated educational handouts given over 3 phases, 6 months apart	–	12 months	**Change in anthropometry and body composition** Weight: −10% (p < 0.001), BMI: −9% (p < 0.001) WC: not measured **Other findings:** 35% increase in knowledge score (p < 0.001), 43.3% increase dietary habits assessment score (p < 0.001) and 17.85% increase in physical activities score (p < 0.001)
Rosmawati et al.^177^ Impact of food nutrition intervention on food handlers' knowledge and competitive food serving: a randomized controlled trial	Food handlers from 16 primary school canteens located in Kota Bharu, Kelantan N = 79	Meso Health education	Food Nutrition Intervention targeting knowledge on food nutrition, healthy food choices, portion sizes and meal planning, encouraging vegetable and milk/milk product servings and reducing unhealthy options. (n = 33)	Usual food handling and serving practices (n = 46)	12 weeks	Anthropometry not measured **Other findings:** Intervention group had significantly greater knowledge about healthy food choice (p < 0.001) and composite knowledge at 12 weeks post intervention than at baseline (p = 0.001). Intervention group had significantly increased number of vegetable menu served by six weeks (p = 0.040) and significantly increased number of milk and milk products served by 12 weeks (p = 0.015) as compared to the control group.

Studies with mainly PA interventions reported minimal reductions in weight (−2.38% to −3.25%), BMI (−1.55% to −3.27%), WC (−1.38% to −4.44%).^153^, ^154^, ^155^, ^156^, ^157^ Yaacob and colleagues showed that both dumbbell exercise and incorporating ankle and wrist weights for daily activities reduced WC (−4.44% and −3.82%, respectively), and improved body composition, although BMI reduction (−0.60% and −0.20%, p > 0.500) was insignificant.^156^ Other studies reported improvements in other parameters such as plasma lipoprotein (a), total physical activity self-regulation, cardiorespiratory fitness.^158^, ^159^, ^160^

Of the 13 publications that assessed the combination of physical activity and dietary interventions, seven were from the My Body is Fit and Fabulous at home (MyBFF@home) program, a government-initiated community-based intervention targeting housewives with overweight/obesity in urban areas of Malaysia.^161^, ^162^, ^163^, ^164^, ^165^^,^^167^^,^^168^ Briefly, intervention comprised a package of individual diet counselling, group exercise and self-monitoring tools given over six months, compared to a control of attending a health seminar. The six-month intervention produced modest improvements in weight (−1.49%), BMI (−1.55%) WC (−3.16%) body composition (%BF −3.15%, visceral fat area: −3.47%); all p < 0.001, and trends in improvement of metabolic risk factors, quality of life, and back pain (p > 0.05). However, follow-up at 12 months showed variable sustainability in these improvements [Weight: −0.66%, BMI: −0.64%, WC: −4.32% (all p < 0.001); visceral fat area: −2.58% (p = 0.004); %BF: −1.55% (p = 0.152)].^166^

One trial in primary care patients with noncommunicable diseases (NCDs) and obesity showed that a behaviour change-based program combining diet and PA improved weight, BMI, and various lifestyle patterns.^169^ Three different workplace interventions ranging three to six months reported modest effects on weight (−1.39% to −7.08%) and BMI (−1.24% to −7.90%).^170^, ^171^, ^172^ Over four months, a structured face-to-face intervention was more effective (weight loss: −7.08%) than online approach (weight loss: −1.39%, p = 0.027).^171^ Group interventions also outperforms individual counselling, being more likely to achieve >6% weight loss over six months.^172^ A community gardening program over three months achieved weight (−3.50%) and BMI reduction (−4.10%), and increased vegetable intake.^173^ Another study over six months found minimal weight change (−1.30%) among women with metabolically unhealthy obesity but demonstrated metabolite shifts even in those with metabolically healthy obesity.^174^

Among unconventional interventions, a virtual program combining psychological therapy and exercise achieved ∼8% reductions in weight and BMI.^175^ while a phased community education tool led to ∼10% weight and 9% BMI reduction alongside better health behaviors.^176^ An intervention with school food handlers improved menu quality, even though weight outcomes were not reported.^177^

The Komuniti Sihat Pembina Negara (KOSPEN) program (not included in Table 3), launched by Malaysia's Ministry of Health in 2014, is a community-led initiative that trains volunteers to promote healthy living, conduct NCD risk screenings, and refer at-risk individuals for care^178^, ^179^, ^180^ Initially implemented in residential areas, it later expanded to workplaces (KOSPEN Plus) and incorporated digital health (KOSPEN@Activ). Reports show good uptake, especially in government agencies, with improved BMI screening and referrals, though high drop-out rates limited impact and large-scale data on obesity outcomes remain lacking.^157^^,^^181^

##### Interpretation

Physical activity in Malaysia varies by sex, sociodemographics, and locality. Women generally report lower overall PA, but higher domestic PA. Discrepancies between self-reported and objectively measured PA highlight the need to reshape perceptions of what constitutes an active lifestyle.^147^^,^^148^ Smartphones offer practical tools for activity tracking, but sustained engagement requires cultural and behavioural shifts.

While modelling studies estimate that minimum requirement of ∼1500 MET-minutes per week for weight loss,^152^ which translates into 6–7 h of moderate PA or 3 h of vigorous PA, this may be difficult to achieve for most Malaysians as 49.9% are classified as sedentary, according to NHMS 2023.^3^ This underscores the importance of combining physical activity with dietary modification, since meaningful and sustainable weight reduction is rarely achievable through exercise alone, as highlighted in the latest Malaysian Obesity CPG.^182^

Lifestyle interventions in Malaysia have generally been short in duration and produced modest weight reduction. Programmes incorporating group-based approaches^172^ and psychological components^175^ show more promise in sustaining behavioural change and should be prioritised in future designs. Moving beyond micro-level interventions, scaling impact will require meso- and macro-level strategies, such as the use of digital tools, improved walkability,^93^ and food vendor education,^177^ which have early evidence for broader population impact.

### Theme 5: Clinical interventions for obesity, barriers and new frontiers

#### Pharmacological

Currently, obesity management medications (OMM) that are available in Malaysia include orlistat, phentermine, bupropion/naltrexone, subcutaneous liraglutide, subcutaneous semaglutide, and subcutaneous tirzepatide. The approved medications, dosages and pharmacokinetics and estimated current retail costs are summarized in Table 4. Current estimated out-of-pocket costs of these medications range between MYR800 and MYR1800 (US$ 170–390) per month. No Malaysian-specific studies have evaluated newer incretin-based therapy such as Glucagon-Like Peptide-1 Receptor Agonists (GLP-1 RA) and Glucose-dependent Insulinotropic Peptide (GIP) agonists, albeit certain trials may have recruited Malaysians as part of larger global cohorts.

**Table 4:** Summary of currently available obesity management medications in Malaysia.

Medication, route of administration, dosage and indication	Mechanism of action	Expected weight loss (Absolute, Percentage of baseline)	Side effects	Contraindications	Estimated retail cost per month
Orlistat Route: Oral Dose: 60–120 mg three times a day with each main meal Adults	Pancreatic lipase inhibitor—inhibits breakdown of dietary triglycerides into absorbable free fatty acids	∼2.6 kg, 2.9–3.4%	Abdominal discomfort Steatorrhea/Faecal incontinence/Flatulence with discharge Reduced absorption of fat-soluble vitamins and certain drugs such as levothyroxine, iodine salts, cyclosporine, anticonvulsants and antiretrovirals Mildly raised liver transaminase Increased urinary oxalate potentially causing acute oxalate nephropathy	Malabsorption syndromes Cholestasis Pregnancy and lactation	MYR 150–210 (US$ 30–40)
Phentermine Route: Oral Dose: 15–30 mg daily Adults and children >12 years of age	Noradrenergic Sympathomimetic—Stimulation of β2-adrenergic receptors in hypothalamus and induce appetite suppression, inhibits monoamine oxidase, potentiating serotonin effect and increasing basal metabolic rate	∼4.43 kg, –	Insomnia Palpitations Increased heart rate Dry mouth	Pulmonary arterial hypertension Existing heart valve abnormalities Moderate-to-severe cerebrovascular disease Severe cardiac disease (arrythmias, advanced arteriosclerosis) Hyperthyroidism History of psychiatric illnesses (anorexia nervosa and depression) Glaucoma History of drug/alcohol abuse Pregnancy and lactation Only indicated for short-term use (3 months) Consider cyclical use	MYR 250–400 (US$ 50–90)
Phentermine/Topiramate Route: Oral Dose: Starting: Phentermine 3.75 mg/Topiramate 23 mg orally once daily for 14 days, followed by: Phentermine 7.5 mg/Topiramate 46 mg orally once daily Phentermine and Topiramate are available separately but not as a combined formulation in Malaysia (as of November 2025) Adults	Phentermine: as above Topiramate: Gamma-aminobutyric acid (GABA) receptor modulator, when used in combination with phentermine: increase energy expenditure, reduce energy efficiency and caloric intake	∼8.8 kg, 8–11%	Paraesthesia, dry mouth, constipation, dysgeusia Mood disturbances: depression, anxiety, irritability Disturbance in attention Increase in heart rate	Same as above	Phentermine: as above Topiramate: MYR 30–150 (US$ 6–30)
Naltrexone/Bupropion Route: Oral Dosage: Naltrexone Hydrochloride 8 mg/Bupropion Hydrochloride 90 mg Twice daily: staggering dose increment by 1 tablet per week, to a maximum of 2 tablets twice a day (Naltrexone hydrochloride 32 mg, bupropion hydrochloride 360 mg) Adults	Naltrexone: Opioid antagonist—blocks opioid-mediated pro-opiomelanocortin (POMC) autoinhibition by endogenous β-endorphins Bupropion: Dopamine and noradrenaline reuptake inhibitor—stimulates hypothalamic POMC neurons to regulate anorectic effects and regulates energy balance	∼4.95 kg, 5–6%	Nausea, Constipation, Vomiting Anxiety Insomnia	Seizures Central Nervous System (CNS) tumours Acute alcohol or benzodiazepine withdrawal Dependent on chronic opioids or opiates (e.g. methadone) Serious psychiatric illnesses (Depression, bipolar disorder, bulimia, anorexia nervosa) Pregnancy and lactation	MYR 400–500 (US$ 90–110)
Liraglutide Route: Subcutaneous Dosage: 1.8 mg daily for type 2 diabetes mellitus, up to 3 mg daily for obesity Adults and children >12 years of age	Glucagon-like Peptide-1 Receptor agonist (GLP-1 RA)—Stimulates incretin-mediated insulin secretion, glucagon suppression, and central appetite suppression	∼5.24 kg, 6–7%	Nausea Vomiting Diarrhoea Constipation Increase in resting heart rate	Personal or family history of Medullary Thyroid Cancer Multiple Endocrine Neoplasia-2 Pregnancy and Lactation Hypersensitivity reactions	MYR 800–1000 (US$ 170–220)
Semaglutide Route: Subcutaneous Dosage: up to 1 mg weekly (Ozempic®) for type 2 diabetes mellitus Up to 2.4 mg weekly (Wegovy®) for obesity Adults	Similar to Liraglutide	∼12.7 kg, Up to 14.9%	Similar to Liraglutide	Similar to Liraglutide Known hypersensitivity to product components Caution in diabetic retinopathy	MYR 700–1200 (US$ 150–300)
Tirzepatide Route: Subcutaneous Dosage: up to 15 mg weekly Adults	GLP1-Glucose-dependent Insulinotropic Peptide (GIP) Receptor Agonist	∼21.9 kg, Up to 20.9%	Nausea, Vomiting Diarrhoea Constipation, Dyspepsia Abdominal pain	Personal/family history of medullary thyroid carcinoma Multiple Endocrine Neoplasia-2 Pregnancy and Lactation Hypersensitivity to tirzepatide.	MYR 800–3000 (US$ 200–730)

Footnotes: Adapted from Clinical Practice Guidelines: Management of Obesity 2nd Edition 2023 (Ministry of Health, Malaysia).

Retail prices are approximate monthly costs based on the Ministry of Health Malaysia Medicine Price Guide 2022 (Pharmaceutical Services Programme, MOH) and local pharmacy listing costs as of August 2026.

#### Metabolic and bariatric surgery

Metabolic and bariatric surgery (MBS) has expanded in Malaysia since its introduction in 1996, with annual procedures increasing from 178 (2010) to 463 (2016), and over 1000 in a single centre (2019).^183^^,^^184^ The cost of MBS in Malaysia ranges between MYR11,000 (US$2433) to MYR30,000 (US$6609) depending on the procedure and setting.^185^ Reported benefits among Malaysian studies include improvements in glycaemia, blood pressure, sleep apnoea, lung function, and cardiac dysfunction,^184^^,^^186^ and cardiac diastolic dysfunction.^187^ Comparative studies found that Roux-en-Y gastric bypass (RYGB) produced greater median excess BMI loss (69.4%) than sleeve gastrectomy (SG: 53.2%, p = 0.016), though both approaches reported comparable diabetes remission rates (RYGB: 54.2% vs SG: 59.0%, p = 0.804) at one year.^188^ An RCT found Omega-3 supplementation comparable to very-low-calorie diet for preoperative liver volume reduction over one month.^189^ Ongoing studies are assessing molecular and nutritional changes postoperatively.^190^ Endoscopic gastric balloon procedures produced modest but sustained weight loss up to one year (weight loss: −10.5% at 4 months and −13.7% at 12 months).^191^ Commonly reported complications post-MBS include hair loss (50.8%), gastroesophageal reflux (49.2%), and vomiting (41.2%).^192^ Pre-existing emotional eating and external eating behaviours are negative predictors of postoperative total weight loss at six months.^193^ Emotional eating and external eating are defined as eating to emotional cues and external cues, respectively. Despite lower food tolerance, patients generally report better health-related quality of life postoperatively.^194^ A qualitative study revealed Malaysian PwO often experienced social restraint, weight bias and stigma but found that MBS brought new life and boosted their self-esteem, despite postoperative physical effects.^195^

#### Barriers and new frontiers of obesity treatment

Although many HCPs directly involved in obesity management (94.3%) recognize obesity as a chronic disease, it is unclear if this view is consistent across the wider Malaysian healthcare system.^196^ Barriers to treatment include high rates of self-weight misperception among PwO,^197^ insufficient exercise, poor hunger control, low motivation, and stigma. From the healthcare worker perspective, challenges include limited patient interest, lack of awareness of treatment options, and time constraints to initiate care.^198^^,^^199^ A separate survey highlighted that HCPs perceived a lack of physical and social opportunities, and require more support to initiate discussions on obesity management in their clinical practice.^196^ Another qualitative study highlighted gaps in skills, social support, and confidence among both patients and providers, with PwO reporting self-blame and hopelessness in their treatment journey.^200^

New avenues are emerging. Community pharmacists are increasingly viewed as potential providers of weight management services, with surveys showing public support for their role in education, monitoring, and support.^201^, ^202^, ^203^ Pharmacists themselves are receptive but noted limited training and therapeutic tools,^202^^,^^203^ while structured education programs may improve readiness.^204^ Digital health and mHealth interventions were variably effective. While face-to-face approaches remain more effective for weight loss, digital programs incorporating peer support and psychological elements may enhance engagement, suggested by a narrative review by Malaysian authors.^205^ Sustained user engagement is critical in ensuring efficacy^29^ and guidance from local IT experts on design features could help ensure cultural fit and long-term sustainability.^206^

##### Interpretation

Studies on clinical interventions of obesity in Malaysia remain scarce and are largely observational and qualitative. The recent Awareness, Care and Treatment in Obesity management (ACTION) Malaysia study revealed that the uptake of OMM in the country is low, with only 10% of all PwO being offered this modality, with both patient and physician citing costs, fear of adverse effects, injection phobia and need for long term use as key barriers.^198^ Other findings were comparable to the ACTION survey in South Korea.^207^ Both studies found high rates of recognition of obesity as a chronic disease among HCPs (∼70–88%). Discussions on weight in clinical settings were infrequent (∼30%) and both groups viewed these efforts as primarily patient-led. Malaysian HCPs more strongly endorsed multidisciplinary care and cited treatment cost as a barrier, while Korean respondents highlighted limited consultation time and patient self-blame as key obstacles to obesity management. These findings are important to inform future public health messaging and to strengthen approaches to clinical obesity management.

Most approved OMMs are currently available in Malaysia, however accessibility and affordability is still questionable.^208^^,^^209^ Increasing demand for incretin therapies, driven by their substantial weight and cardiometabolic benefits, has generated pressures in global supply chains^209^ and even proliferated counterfeit products.^208^ More importantly, the costs of these medications are prohibitive, realistically unaffordable for the average Malaysian with median monthly income of ∼MYR 2800 (US$ 700).^210^ MBS while very effective in weight loss and metabolic improvements, is generally limited by long-term side effect tolerability, cost, and access to surgeons. Nevertheless, the field of bariatric surgery has seen robust growth locally over the last decade.

The Malaysian reimbursement model does not provide universal coverage for clinical obesity treatment, except for patients under civil service or pensioner schemes, who may receive coverage for approved indication of diabetes. This is similar in neighbouring Thailand and Singapore.^211^ Nonetheless there are calls in Thailand to make high-cost medications more accessible via alternative pathways based on specific criteria.^212^ The denial of OMMs for treatment of obesity is similar even in other Western high-income countries, often due to perceived high costs or cost-effectiveness concerns.^213^ Nonetheless, France and Iceland provide a model for equitable and ethical OMM access, by prioritizing PwO with more severe obesity and comorbidities.^213^

### Malaysian obesity landscape in perspective

#### Past and present

The current quoted Malaysian prevalence of overweight/obesity of 54.4% at (BMI ≥25 kg/m^2^) likely under-represents the true at-risk populations (BMI ≥23 kg/m^2^), which goes up to 70.1%.^3^ Malaysia's shift from nutritional scarcity to caloric abundance has not been matched by evolutions in research capacity or policy implementations to encourage healthy lifestyles. Existing Malaysian policy responses include the SSB Tax implemented only in 2019, National Strategic Plan for Non-Communicable Diseases (NSP-NCD), the NPANM and the NRP framework, but the implementation and monitoring remain inconsistent. Compared with its closest socioeconomic peer, Thailand [obesity prevalence: 15.4%, GDP adjusted for Purchasing Power Parity (GDP PPP): 24,708 $], Malaysia's obesity prevalence is ∼1.5 times higher (Supplementary Table S1), despite both countries' similar geographical locations and development trajectories. Thailand's long-standing policies: such as school soda bans (2008), SSB taxation (2017), and launching the ‘Fatless Belly Thais’ campaign (2007), are made successful by strong political will, multisectoral collaboration and emphasis on sustainability.^214^ Singapore, while sharing a similar geopolitical history and ethnic composition, is much wealthier (GDP PPP: 150,689 $) and considerably leaner overall (obesity prevalence: 15.4%). Nevertheless, it has implemented initiatives such as the ‘Healthier SG’ and the ‘Nutri-Grade’ front-of-pack labelling policy as comprehensive national responses towards the obesity epidemic.^215^

#### Future directions

Epidemiological impact data suggests that current local definition of obesity (BMI ≥27.5 kg/m^2^) needs critical revision. Future work should extend to longitudinal, interventional, and life-course design, incorporate refined definitions of adiposity, comprehensive metabolic health and functional outcomes. Strong obesity-focused socioeconomic outcome analyses are sorely needed to guide resource allocation.

Interventional research must guide treatment sequencing with clear frameworks, based on long-term outcomes and cost-effectiveness of expensive but highly effective obesity pharmacotherapy and MBS. Clear definitions of referral criteria, practical measures for multidisciplinary integration are important to ensure equitable access to advanced therapies balancing ethical and economic considerations. As demand to costly therapies increases, enhancement of behavioural and lifestyle interventions is more important than ever. Scalability should be drawn upon unique lessons learnt from prior local and international approaches. Novel approaches should be explored. Implementing more meso-level interventions for more broader population impact, task-shifting to other HCPs such as community pharmacists to expand obesity care delivery, and adoption of digital health with careful considerations of its limitations, are notable examples of lessons drawn from our current evidence mapping.

Policy and implementation research should embed implementation science approaches to examine real-world uptake, enforcement and outcomes. Strengthening research collaboration and integration across fields beyond healthcare experts is important to innovate new strategies, but routine assessment of outcomes is equally important to ensure sustainability. Overall, our recommendations reiterate that of a recent review that endorses coordinated, multidisciplinary approaches supported by strong policies and evidence-based research to manage obesity as a chronic disease.^216^

## Strength and limitations

Our work emulates the strengths of the two previous similar reviews, Lim KG's broad synthesis,^6^ and Mohamed Nor et al.‘s structured approach in categorizing obesity research.^7^ We mapped updated evidence across biological, behavioural and policy domains mapping against a standardized framework and synthesized a thorough thematic overview, providing a template for future assessment of research progress. As a narrative mapping review, selection and interpretive bias cannot be fully excluded. Nevertheless, we employed an exhaustive search strategy to minimize bias. Local-language articles were not included as local academic publications are predominantly in English or bilingual Malay-English. We limited the scope to non-pregnant adults, as incorporating pregnancy and paediatric evidence would have been too extensive for the format of this review. We believe complementary reviews focusing on these populations are warranted.

## Conclusion

Despite the bleak outlook, World Obesity Federation ranks Malaysia's preparedness to address obesity as ‘fairly good’ on a global scale, considering the current healthcare system and policy responses to NCDs in place. We believe the landscape provided in this review partially reflects this, given the local scientific community's enthusiastic response to the national obesity epidemic, yet substantial challenges lie ahead. A coordinated, whole-of-society approach will be essential to reverse current trends and mitigate this growing burden.

## Contributors

QHL: conceptualization, data analysis, writing–reviewing and editing. JKK: data analysis, writing, YGO: data-analysis and editing, AR: data analysis and editing, JR: conceptualization, supervision, reviewing and editing.

## Declaration of interests

QHL has received speaker honoraria and travel support from Novo Nordisk, AstraZeneca, Zuellig Pharma, Viatris, and Boehringer Ingelheim; has served as a co-investigator in an industry-sponsored clinical trial funded by Novo Nordisk; and is a Council Member of the Malaysian Obesity Society. YGO reports that Boehringer Ingelheim provided support to the secretariat of the National Obesity Scientific Meeting for meeting attendance, with no payments made directly to the author. The remaining authors declare no competing interests.
