# Supplementary material for: The landscape of adult obesity in Malaysia: a mapping of evidence and contemporary narrative review
Source: Lancet Reg Health West Pac. 2026 Aug 27;74:101963. doi: 10.1016/j.lanwpc.2026.101963 (PMC13544182; doi:10.1016/j.lanwpc.2026.101963)
Supplement: Supplementary file 1 [file mmc1.docx]

**Supplementary Materials Index:**

**Supplementary Figure 1:** Reports included in review on obesity in Malaysia including additional sources used to support contextual discussion – Page 2

**Supplementary Figure 2**: Characteristics of Studies on Obesity in Malaysia by Research Scope– Page 3

**Supplementary Table 1**: Global, Western Pacific Region (WPR), and Association of Southeast Asian Nation (ASEAN) Rankings of Country Obesity Prevalences and Income Level – Page 4

**Supplementary Table 2**: Summaries of key findings of studies on obesity epidemiology in Malaysia from 2016 to 2024 (Purpose A) – Page 5

**Supplementary Table 3**: Summaries of key findings of studies on obesity interventions in Malaysia from 2016 to 2024 (Purpose B) - Page 40

**Supplementary Table 4**: Summaries of key findings of studies developing new modalities for investigating obesity in Malaysia from 2016 to 2024 (Purpose C) – Page 58

**Reports Identified to Support Discussion**

**Identification of studies via databases and registers**

Government technical reports (n = 8)

Relevant international / regional reports and studies (n=22)

Media articles (n = 3)

Malaysian studies outside review period (n=4)

Duplicates removed

(n = 1139)

Records identified

Google Scholar (n= 1021),

PubMed (n = 102)

Web of Science (n= 2506)

(n=3626)

**Identification**

Excluded (n=2300):

Non-human studies (n = 200)

Studies on adolescents (n = 214)

Studies on pregnant women (n=113)

Studies by Malaysian authors not focused on obesity (n = 1773)

All records were available

(n=37)

Titles and Abstracts screened

(n = 2487)

**Screening**

Reports sought for retrieval (n=187)

Full reports not available (n=8)

Eligible and available studies

(n =179)

**Included**

Reports classified according to Purpose and Research Scope and synthesis of narrative review

Total reports included in review

(n = 216)

**Supplementary Figure 1:** Reports included in review on obesity in Malaysia including additional sources used to support contextual discussion

**Supplementary Figure 1**: **Characteristics of Studies on Obesity in Malaysia by Research Scope**

Studies on obesity in Malaysia published between January 2016 to December 2024, stratified by study design and research scopes. Colour of bubbles indicate data source, and bubble sizes are proportionate to number of studies. Research Scopes: A1: Relationship between Waist Circumference (WC), Waist-Hip Ratio (WHR), Waist-Height Ratio (WHtR) and Body Mass Index (BMI) on Non-Communicable Diseases (NCDs), A2: Relationship between Adiposity and Noncommunicable Diseases Risk Factors, A3: The Impact of Obesity on Social and Economic Cost, A4: Association of Dietary Intake, Appetite Control, Eating Behaviour and Inflammatory Status with Obesity, A5: Determination of Socio-Cultural Factors Influencing Obesity, A6: Effect of Metabolic Predisposition to Onset of Obesity, A7: Association between Physical Inactivity, Sedentary Lifestyle and Obesity.A8: Determination of Genetic Factors Influencing Development of Overweight and Obesity, A9: Obesity and COVID-19, B1: Development and Evaluation Of Obesity Prevention and Intervention Programmes, B2 Development and Evaluation Of Obesity Management Programmes, B3: The Impact of Policies and Environment (Food and Physical Activity) on Obesity, C1: Identification of New Methods to Define Obesity, C2: Identification of Novel Strategies to Prevent and Manage Obesity

**Supplementary Table 1**: Global, Western Pacific Region (WPR), and Association of Southeast Asian Nation (ASEAN) Rankings of Country Obesity Prevalences and Income Level

| **Global Rank** | **Regional Rank (WPR)** | **Regional Rank (ASEAN)** | **Country / Area** | **Adult Obesity Prevalence (%)** | **Income Group** | **Most Recent GDP PPP (Current International $)** |
| --- | --- | --- | --- | --- | --- | --- |
| 1 | 1 | - | American Samoa | 75‧9 | High | N/A |
| 2 | 2 | - | Tonga | 72‧4 | Upper-middle | 7,853 |
| 3 | 3 | - | Nauru | 71‧1 | High | 14,327 |
| 4 | 4 | - | Tokelau | 71‧0 | — | N/A |
| 5 | 5 | - | Cook Islands | 69‧6 | — | N/A |
| 6 | 6 | - | Niue | 67‧3 | — | N/A |
| 7 | 7 | - | Tuvalu | 65‧3 | Upper-middle | 6,151 |
| 8 | 8 | - | Samoa | 63‧7 | Upper-middle | 7,837 |
| 9 | 9 | - | French Polynesia | 49‧0 | High | N/A |
| 10 | 10 | - | Micronesia (FSM) | 48‧2 | Lower-middle | 4,346 |
| 12 | 11 | - | Marshall Islands | 47‧1 | Upper-middle | 8,198 |
| 13 | 12 | - | Kiribati | 46‧9 | Lower-middle | 3,702 |
| 21 | 13 | - | Palau | 42‧1 | High | 17,532 |
| 35 | 14 | - | Fiji | 34‧9 | Upper-middle | 16,032 |
| 40 | 15 | - | New Zealand | 34‧3 | High | 55,094 |
| 53 | 16 | 1 | Brunei Darussalam* | 32‧1 | High | 90,007 |
| 58 | 17 | - | Australia | 31‧0 | High | 71,193 |
| 89 | 18 | - | Mongolia | 24‧8 | Upper-middle | 19,098 |
| 100 | 19 | - | Solomon Islands | 23‧3 | Lower-middle | 2,873 |
| **108** | **20** | **2** | **Malaysia*** | **22**‧**4** | **Upper-middle** | **38,728** |
| 111 | 21 | - | Vanuatu | 22‧1 | Lower-middle | 3,602 |
| 118 | 22 | - | Papua New Guinea | 21‧2 | Lower-middle | 4,889 |
| 149 | 23 | 3 | Singapore* | 14‧0 | High | 150,689 |
| 143 | - | 4 | Thailand* | 15‧4 | Upper-middle | 24,708 |
| 163 | - | 5 | Indonesia* | 11‧5 | Upper-middle | 16,448 |
| 175 | 24 | 6 | Philippines* | 9‧0 | Lower-middle | 11,794 |
| 178 | 25 | - | China | 8‧4 | Upper-middle | 27,105 |
| 179 | 26 | 7 | Lao PDR* | 8‧3 | Lower-middle | 9,788 |
| 182 | - | 8 | Myanmar* | 7‧7 | Lower-middle | 5,998 |
| 191 | 27 | - | Japan | 5‧6 | High | 51,685 |
| 196 | 28 | 9 | Cambodia* | 4‧5 | Lower-middle | 7,970 |
| 199 | - | 10 | Timor-Leste* | 2‧4 | Lower-middle | 4,758 |
| 200 | 29 | 11 | Vietnam* | 2‧0 | Lower-middle | 16,386 |
| - | - | - | Hong Kong SAR | 32‧6 | High | 75,216 |
| Obesity was defined as Body Mass Index≥30 kg/m^2^  Countries/regions with no available data: Guam, Macao SAR, New Caledonia, Northern Mariana Islands, Wallis and Futuna, and Pitcairn Islands.  Hong Kong SAR and Macao SAR are administrative regions of the People’s Republic of China but are listed separately owing to distinct data availability and health system characteristics.  *ASEAN countries  Sources:  Global Obesity Observatory, World Obesity Federation 2025. NCD Risk Factor Collaboration (NCD-Risch). Worldwide Trends in Underweight and Obesity from 1990 to 2022. Lancet. 2024.  World Bank. GDP per capita, PPP (current international $). Accessed November 2025.  World Health Organization. Western Pacific Region Member List. 2025.  Abbreviations: FSM= Federated States of Micronesia; GDP PPP = Gross domestic product adjusted for purchasing power parity; PDR=People’s Democratic Republic; SAR = Special Administrative Region; WPR = Western Pacific Region. | | | | | | |

**Supplementary Table 2**: Summaries of key findings of studies on obesity epidemiology in Malaysia from 2016 to 2024 (Purpose A)

| **Research Scope** | **Studies** | **Data Source** | **Design** | **Key Findings** |
| --- | --- | --- | --- | --- |
| A1. Relationship between waist circumference (WC), waist-hip ratio (WHR), waist-height ratio (Whirr) and body mass index (BMI) on non-communicable diseases (NCDs).  n=7  Data Source:  Population: 1  Community: 6  Study Type:  Cross-sectional: 7  Study Design:  Observational: 7 | Ahmad et al., 2016  Abdominal obesity indicators: Waist circumference or waist-to-hip ratio in Malaysian adult population | Community  Adults aged >18 years of age, from a rural community in Tanjung Karang, Selangor.  N=669 (59.2% female; average age not specified) | Cross-sectional, observational  Correlation of WC and WHR with BMI  Compare prevalence of abdominal obesity based on WHO Asian and Non-Asian cut-off | WC stronger correlation with BMI than WHR.  Male WC r = 0.78, male WHR r = 0.24; female WC r = 0.72, female WHR r = 0.19; P < 0.001.  Non-Asian WHO BMI cut off: Underweight 10.6%, Normal 45.1%, Overweight 26.8%, Obesity 17.4%.  Asian WHO BMI cut off: Underweight 10.6%, Normal 29.2%, Overweight 32.3%, Obesity 27.7%.  Non-Asian WHO WC cut off for abdominal obesity: Male 23.8%, Female 66.4%.  Asian WHO WC cut off for abdominal obesity: Male 37.4%, Female 66.4%.  Non-Asian WHO WHR cut off for abdominal obesity: Male 6.2%, Female 54.2%.  Asian WHO WHR cut off for abdominal obesity: Male 26.4%, Female 75.0%. |
|  | Low et al., 2022  Comparing the performance of body mass index, waist circumference and waist-to-height ratio in predicting Malaysians with excess adiposity | Community  Adults aged ≥40 years from Klang Valley  N=399  (52.6% female, mean age: 56.1±8.1 years; 47.5% male, mean age 57.8±9.6 years) | Cross-sectional, observational  Compare performance of BMI, WC, WHR with DXA-defined excess adiposity | Optimal performance of WHtR at the standard cut-off value of 0.5 (AUC: 0.664 (95% CI 0.585,0.743), sensitivity: 96.6%, specificity: 36.1%)  Performance of BMI and WC required adjustments.  BMI threshold of 25 kg/m^2^ adjusted to 22.2 kg/m^2^ for women and 24.2 kg/m^2^ for men improved AUC from 0.709 (95% CI 0.649, 0.769) to 0.792 (95% CI 0.734,0.850), sensitivity from 55.7% to 76.5%, and specificity from 86.1% to 81.9%.  WC threshold of 80 cm adjusted to 76 cm for women and 90 cm to 85 cm for men improved AUC from 0.709 (95% CI 0.649, 0.769) to 0.772 (95% CI 0.708-0.836), sensitivity from 62.7% to 80.7% and specificity from 90.3% to 73.6%. |
|  | Nadeem et al., 2021  Correlation of anthropometric indices with lipid profile indices among Malay obese and non-obese subjects in Malaysia | Community  Malay Adults aged 18-60 years in Terengganu  N=193 (63.7% Female, Pooled mean age: 36.2±5.8 years)  *Only Malays ethnicity group were represented | Cross-sectional, observational  Regression analysis of BMI, WC, HC, WHR, WHtR with lipid metrics: Triglyceride (TG), Total Cholesterol (TC), Low-density Lipoprotein (LDL), High-density Lipoprotein (HDL). | BMI positively associated with TG [B=17., SE=5.13, p=0.001], LDL (B=15.5, SE=6.69,p=0.014).  BMI negatively associated with TC (B=-14.9, SE=6.45, p=0.022).  WC positively associated with TG (B=14.6, SE=4.13, p=0.001).  WHR positively associated with TC (β=0.22, SE=0.09; p=0.010), TG (β= 0.05, SE=0.02, p=0.002).  WHR negatively correlate with LDL (β =-0.28, SE=0.10, p=0.004).  All results above were independent of sex, age and prevalent obese status. |
|  |  |  |  |  |
|  | Ching et al., 2020  Comparisons of conventional and novel anthropometric obesity indices to predict metabolic syndrome among vegetarians in Malaysia  (Also in A2) | Community  Adult vegetarians aged 18 to 74  N=273 (64.8% female, pooled mean age 47.6±13.1 years)  *Only Malaysian Chinese and Indians ethnicity groups were represented | Cross-sectional, observational  ROC curve analysis of BMI, body fat percent (%BF), WHtR in predicting metabolic syndrome  Metabolic syndrome definition: Joint Interim Statement 2009 | WHtR outperforms BMI and %BF in predicting metabolic syndrome.  Men  -WHtR of 0.54 (AUC: 0.825; 95% CI 0.739,0.910, sensitivity 82.1%, and specificity 75.0%)  -BMI of 23.00 kg/m^2^ (AUC: 0.777; 95% CI 0.686,0.898, sensitivity 100%, and specificity 47.1%)  -%BF of 28.05% (AUC 0.782; 95% CI 0.685,0.878, sensitivity 78.6%, and specificity 75.0%)  Women  -WHtR of 0.532 (AUC: 0.863; 95% CI 0.797,0.928, sensitivity 81.6%, and specificity 79.9%)  -BMI of 24.05 kg/m^2^ (AUC: 0.847; 95% CI 0.782,0.913, sensitivity 84.2%, and specificity 73.4%)  -%BF of 34.5% (AUC 0.785; 95% CI 0.708,0.863, sensitivity 71.1%, and specificity 72.7%) |
|  | Aizuddin et al., 2021  Performance of body mass index in identifying obesity defined by body fat percentage and hypertension among Malaysian population: A retrospective study | Community  Participants in a health screening in Kuala Lumpur.  N=136 (66.9% female, 62.5% <40 years old) | Cross-sectional. Observational  ROC curve analysis of BMI against bioelectric impedance analyser (BIA)-measured %BF  Cut-off: %BF >25% in men; >35% in women | ROC curve analysis:  Suggested new BMI cutoff 24.8 kg/m^2^ (κ=0.722, AUC: 0.867, sensitivity 80%, and specificity 34%)  Malaysian BMI cutoff 27.5 kg/m^2^ (κ=0.5322, AUC: 0.778, sensitivity 56%, and specificity 98.4%)  Cross-tabulation:  Suggested new BMI cutoff 24.8 kg/m^2^ predict hypertension with sensitivity of 63.9%, specificity 59% vs BMI 27.5 kg/m^2^ sensitivity 36.1%, specificity 69.0%. (from cross-tabulation, not ROC curve) |
|  | Teh et al., 2023  Body mass index and waist circumference trajectories across the life course and birth cohorts, 1996–2015 Malaysia: sex and ethnicity matter | Population  Pooled data from four population-based NHMS census.  1996 (N=22,631)  2006 (N=34,184)  2011 (N=18,017)  2015 (N= 19,705)  Adult aged 18–80 years | Cross-sectional. Observational  Hierarchical Age-Period-Cohort (HAPC) analysis of BMI and WC trajectories across life course and birth cohorts by sex and ethnicity. | The prevalence of both general and abdominal obesity has been increasing from 1996–2015.  HAPC result:  -BMI for both males and females increased steeply from age 18 to. 60 years and then plateaued.  -WC increased monotonically across life course, more profound in female.  -Chinese have lesser BMI and WC increases across life course and birth cohorts than other ethnic groups. |
|  |  |  |  |  |
|  | Chua et al., 2017  Obesity indices predict hypertension among indigenous adults in Krau Wildlife Reserve, Peninsular Malaysia | Community Adults aged ≥18 years across 20 *Orang Asli* (indigenous peoples) villages in Krau Wildlife Reserve, Pahang.  N=482 (53.7% female, mean age 35.4 years) | Cross-sectional, observational  ROC curve analysis of WHtR, BMI, WC, WHR against hypertension status. | WHtR best predict hypertension, in both men and women.  Men:  WHtR of 0.45 (AUC 0.66, 95% CI 0.59,0.74, Sensitivity 84.3%, Specificity 42.5%)  BMI of 23.54 kg/m^2^ (AUC 0.55, 95% CI 0.47,0.64, Sensitivity 51.4%, Specificity 64.1%)  WC of 76.0 cm (AUC 0.65, 95% CI 0.57,0.73, Sensitivity 62.9%, Specificity 69.3%)  WHR of 0.81 (AUC 0.65, 95% CI 0.57,0.72, Sensitivity 81.4%, Specificity 49.0%)  Women:  WHtR of 0.52 (AUC 0.60, 95% CI 0.52,0.68, Sensitivity 62.3%, Specificity 55.8%)  BMI of 24.72 kg/m^2^ (AUC: 0.59, 95% CI 0.50,0.67, Sensitivity 58.1%, Specificity 67.0%)  WC of 75.0 cm (AUC 0.61, 95% CI 0.53,0.69, Sensitivity 69.8%, Specificity 58.7%)  WHR of 0.82 (AUC 0.67, 95% CI 0.60,0.74, Sensitivity 79.2%, Specificity 57.3%) |
| A2. Relationship between adiposity and NCDs risk factors  n=21  Data Source:  Population: 5  Community: 14  Clinical: 2  Study Type:  Cross-sectional: 16  Retrospectivel: 5  Study Design:  Observational: 20  Cohort: 1 | Ching et al., 2020  Comparisons of conventional and novel anthropometric obesity indices to predict metabolic syndrome among vegetarians in Malaysia  (Also in A1) | Community  Adult vegetarians aged 18 to 74  N=273 (64.8% female, pooled mean age 47.6±13.1 years)  *Only Malaysian Chinese and Indians ethnicity groups were represented | Cross-sectional, observational  ROC curve analysis of novel markers: Lipid Accumulation Product (LAP), Visceral Adiposity Index (VAI), Body Shape Index (ABSI) and Body Roundness Index (BRI) in predicting metabolic syndrome  Metabolic syndrome definition: Joint Interim Statement 2009 | LAP was a better predictor to predict metabolic syndrome than other novel obesity indices.  (Analysis result for conventional obesity indices refer to row above)  Men  -LAP of 41.44 (AUC: 0.923; 95% CI 0.867,0.980, sensitivity 85.7%, and specificity 85.3%)  -VAI of 2.231 (AUC: 0.864; 95% CI 0.786,0.942, sensitivity 82.1%, and specificity 83.8%)  -ABSI of 0.780 (AUC 0.652; 95% CI 0.534,0.770, sensitivity 67.9%, and specificity 57.4%)  - BRI of 2.248 (AUC 0.583; 95% CI 0.457,0.709, sensitivity 42.9%, and specificity 77.9%)  Women  -LAP of 21.74 (AUC: 0.920; 95% CI 0.862,0.977, sensitivity 94.7%, and specificity 70.5%)  -VAI of 1.965 (AUC: 0.882; 95% CI 0.818,0.947, sensitivity 84.2%, and specificity 87.1%)  -ABSI of 0.777 (AUC 0.636; 95% CI 0.536,0.735, sensitivity 63.2%, and specificity 61.9%)  - BRI of 2.714 (AUC 0.537; 95% CI 0.436,0.639, sensitivity 57.9%, and specificity 57.6%) |
|  | Shahadan et al., 2018  Abdominal obesity and high-sensitivity C-reactive protein level among Malay obese adults in Kuantan, Malaysia | Community  Healthy obese Malay adults with BMI 27.5-39.9 kg/m^2^ from Kuantan  N=93 (67.7% female, pooled mean age 33.6±9.6 years)  *Only Malay ethnicity group were represented | Cross-sectional, observational  Correlation of WC with hs-CRP level (Kendall’s Tau) | WC positively correlated with hs-CRP in obese Malay adults.  Men: r = 0.355, p = 0.006.  Women: r = 0.177, p = 0.043. |
|  | Chong et al., 2023  Prevalence of overweight and its associated factors among Malaysian adults: Findings from a nationally representative survey  (Also in A6) | Community  Adults aged ≥18 years old from NHMS 2019  (nationally representative sample)  N=9782 (47.6% female, mean age not reported) | Cross-sectional, observational  Prevalence of obesity and  multiple logistic regression analysis of independent factors associated with obesity and NCD  Overweight: BMI 25 - 29.9 kg/m^2^  Obesity: BMI ≥ 30 kg/m^2^ | Increasing rate of overweight-obesity in Malaysia: 44.5% (2011), 47.7% (2015), 50.1% (2019).  Overweight significantly higher risk of NCD  -Diabetes (adjusted OR= 1.47; 95% CI 1.23, 1.75; p < 0.001).  -Hypertension (adjusted OR = 2.60; 95% CI 2.20, 3.07; p < 0.001).  -Hypercholesterolaemia (adjusted OR = 1.16; 95% CI 0.99, 1.35; p < 0.001).  Independent association of overweight  -Female gender (adjusted OR 1.33; 95% CI 1.11, 1.58; p = .002),  -Ages 30–59 years (adjusted OR 1.61; 95% CI 1.31, 1.97; p < .001)  -Malay (adjusted OR 1.68; 95% CI 1.36, 2.07; p < .001);  -Indian (adjusted OR 2.59; 95% CI 1.80, 3.74; p < .001);  -other Bumiputera (adjusted OR 1.82; 95% CI 1.38, 2.39; p < .001)  -Married (adjusted OR 1.23; 95% CI 1.00, 1.50; p = .046),  -Adequate health literacy (adjusted OR 1.19; 95% CI 1.01, 1.39; p =.033). |
|  | Johari et al., 2017  High body fat percentage among adult women in Malaysia: the role of lifestyle | Community  Adult women aged 40-59 years old from urban area in central Malaysia.  N=327 (mean age 50.4±5.6 years) | Cross-sectional, observational  Regression analysis for lifestyle predictors of high %BF (defined as >33%) | Lower protein intake, low higher calcium intake, and lower intensity of household-related physical activity were independently associated with higher %BF. |
|  | Moy and Loh, 2015  Cardiometabolic risks profile of normal weight obese and multi-ethnic women in a developing country  (Also in C1) | Community  Female secondary school teachers with normal BMI from multi-stage sampling across six states  N=1095 (Mean age: 40.5±9.0 years) | Cross-sectional, observational  Normal weight obese (NWO) defined as normal BMI for Asians and the highest tertile of %BF  Prevalence of NWO.  Multivariate logistic regression to estimate OR of cardiometabolic risks across %BF tertiles, adjusted for age and ethnicity. | Prevalence of NWO women: 19.8%  Prevalence of metabolic syndrome among NWO women was 5.4%.  Regression analysis for factors associated with high %BF  Women with NWO had higher odds for  -Abdominal obesity (adjusted OR 2.64; 95% CI 1.73, 4.04; p<0.05)  -Hypertriglyceridemia (adjusted OR 2.51; 95% CI 1.47, 4.29; p<0.05)  -Hypertension (adjusted OR 1.63; 95% CI 1.15, 2.31; p<0.05) |
|  | Abidin, 2023  The “Fat or Fit Paradox” in postmenopausal women: a cross-sectional study on the association between skeletal muscle mass, adiposity, and muscle strength | Community  Postmenopausal women from Semenyih and Klang Valley  N=134 (Mean age: 60.4±7.5 years) | Cross-sectional, observational  Assessment of body composition measured by bioimpedance analysis and muscle strength measured by dynamometer  Linear regression between muscle strength and skeletal muscle mass index (ASMI) | Positive correlation (r=0.359, p<0.001) between appendicular ASMI and %BF.  ASMI was predictive of muscle strength, but the association reduced with increasing obesity categories. |
|  | Shafinaz and Moy, 2016  Vitamin D level and its association with adiposity among multi-ethnic adults in Kuala Lumpur, Malaysia: a cross-sectional study | Community  Teachers from secondary schools in Kuala Lumpur  N=858 (90.9% female, mean age not reported) | Cross-sectional, observational  Assessment of anthropometry, body composition and serum Vitamin D levels | Overall prevalence of vitamin D deficiency (<20 ng/ml) was 67.4 %.  Higher BMI and larger WC (p < 0.05) significantly associated with lower serum 25(OH)D level.  25(OH)D level negatively correlated with BMI (β = −0.23) and %BF (β = −0.14) |
|  | Cheah et al., 2016.  Gender and racial differences in the cardiovascular risk factors among overweight and obese rural adults, Kuching and Samarahan Division, Sarawak, Malaysia | Community  Adults aged 18-65 years old from rural communities in Kuching and Samarahan division, Sarawak  N=155 (78.1% female, mean age: 45.1±10.2 years) | Cross-sectional observational  Analysis of anthropometry, body composition, and cardiovascular risk estimated by atherogenic index of plasma (AIP) | Bidayuh ethnicity group (compared to Malays ethnicity)  -Higher odds of having higher %BF (OR:4.9, 95%CI 1.22,7.89).  -Lower levels of AIP-estimated cardiovascular risk (OR: 0.30, 95%CI 0.12,0.78).  Iban ethnicity group (compared to Malays ethnicity)  -Higher odds for hyperglycaemia (OR: 3.86, 95%CI 1.06, 6.66).  -Lower AIP (OR 0.29, 95%CI 0.12,0.69). |
|  | Aghakhanian et al., 2019.  Metabolic syndrome and cardiometabolic risk factors among indigenous Malaysians  (Also in A5,A9) | Community  People from Indigenous communities across peninsula Malaysia aged >18 years.  N=629 (Females 41.8%) | Cross-sectional, observational  Analysis of anthropometry, plasma glycaemic and lipid profiles | Prevalence of obesity 52.1%, and abdominal obesity was 72.6%.  Metabolic syndrome prevalence 29.57%; more prevalent in females than males (35.25%;21.95%, p<0.001). |
|  | Hashim et al., 2024.  Association between weight and body composition changes with falls risk in the Malaysian Elders Longitudinal Research (MELoR) study. | Community  Community-dwelling older adults aged ≥55 years, part of the Malaysian Elders Longitudinal Research (MELoR) study  N=225 (mean age: 71.8±6.8 years, Female: 56.9%) | Retrospective, observational  Analysis, baseline (2013–2016) and follow-up (2020–2022) cohorts for falls incidence over 12 months, and associations with changes in weight and body composition. | Weight gain of ≥ 5% was associated with increased fall risk at follow-up compared with stable weight.  This relationship attenuated by low baseline percentage lean body mass among women. |
|  | Singh et al., 2020  Obese and disease-free (ODF) population in Malaysia: Findings from National Health and Morbidity Survey (NHMS) Malaysia 2015 | Population (NHMS)  N= not reported clearly | Cross-sectional, observational  Prevalence of ODF | Overall prevalence of ODF: 23.7% |
|  | Koo et al., 2023  Obesity and its association with undiagnosed diabetes mellitus, high blood pressure and hypercholesterolemia in the Malaysian adult population: A national cross-sectional study using NHMS data. | Population (NHMS)  N= 13024 (Female 50.8%, mean age not reported) | Cross-sectional, observational  Evaluation association between obesity and other NCDs | BMI defined overweight/obese group (Asian cut-off)  -undiagnosed diabetes mellitus (aOR: 1.65, 95% CI: 1.31–2.07)  - high blood pressure (aOR: 3.08, 95% CI: 2.60–3.63)  - hypercholesterolemia (aOR: 1.37, 95% CI: 1.22–1.53)  Central obesity  -undiagnosed diabetes mellitus (aOR: 1.40, 95% CI: 1.17–1.67)  -high blood pressure (aOR: 2.83, 95% CI: 2.45–3.26)  -hypercholesterolemia (aOR: 1.26, 95% CI: 1.12–1.42) |
|  | Daud and Shahadan, 2017  Association between body mass index and cardiometabolic risks among Malay obese adults | Community  Malay obese adults from suburban areas of Kuantan, Pahang  N= 82 (Mean age 34.1 ±9.5 years, 69.5% female) | Cross-sectional, observational  Evaluate association between cardiometabolic risk factors with BMI among obese adults | Weak correlation between BMI and systolic blood pressure (r =0.276), diastolic blood pressure (r=0.311) and hs-CRP (r=0.384). |
|  | Riyahi et al., 2018  Platelet-related cytokines among normal body mass index, overweight, and obese Malaysians | Community  Staffs and students from Universiti Putra Malaysia  N= 112 (mean age not reported, 54.5% female) | Cross-sectional, observational  Investigate association between novel inflammatory markers with obesity  -Soluble CD40 ligand (sCD40L)  -Soluble P selectin (sP-sel)  -Mean platelet volume (MPV)  -Platelet count | Soluble P selectin (sP-sel) correlated significantly with BMI (r =0.36) and WC (r =0.25)  Mean platelet volume (MPV) correlated significantly with BMI (r =0.2) and WC (r =0.2) |
|  | Shaharuddin et al., 2023  Investigating the prevalence of diabetic complications in overweight/obese patients: A study in a tertiary hospital in Malaysia | Clinical  Registry at hospital Serdang - sub analysis of cohort with type 2 diabetes  N= 495 (mean age 52.2 years, female 57%) | Cross-sectional, observational  Prevalence of obesity and diabetic complications among diabetic patients in Malaysia | 91% are overweight/ obese. No difference in rate of diabetes-related complications among normal BMI versus overweight/obese BMI group. |
|  | Al-Mahmood et al., 2016  The metabolic syndrome in normal weight Malay subjects | Community  Malay community in Kota Bharu, Peninsula Malaysia with BMI <25kg/m^2^  N= 226 (57.5% female) | Cross-sectional, observational  Determine the prevalence of metabolic syndrome (MS) in normal body weight normoglycemic Malay subjects.  MS defined by AHA/NHLBI (≥3/5) or IDF (central obesity +2); association between MS features and HOMA%S assessed by ANOVA. | MS criteria prevalence (%)  -High waist circumference 33.93%  -Low HDL-Cholesterol 19.58%  -High Triglyceride 25.56%  -High fasting plasma glucose 4.15%  -High blood pressure 67.41%  Addition of each feature of MS was associated with reduced insulin sensitivity. (p<0.001 for trend) |
|  | Teh and Woon, 2021  Burden of cancers attributable to modifiable risk factors in Malaysia | Population (NHMS 2006 and Globocan 2018 data)  Pooled data from multiple database/registry | Retrospective, observational  Estimate the Population Attributable Fraction (PAF) of the modifiable risk factors to cancers incidences in Malaysia. | 22.2% (95% confidence interval (CI):14.9 to 29.6%) of the cancer incidences included in this study were attributable to the investigated modifiable risk factors.  The top main cancers attributed by the risk factors were lung cancer (65.1%; 95% CI:56.4 to 72.9%), laryngeal cancer (63.6%; 95% CI:39.9 to 80.5%), and oesophageal cancer (51.5%; 95% CI:39.9 to 62.0%). |
|  | Kee et al., 2017  Association of BMI with risk of CVD mortality and all-cause mortality  (Also in A3) | Population (All adult participant in NHMS 2006, followed up 5 years with mortality data from Malaysia National Registration Department)  N=32829 | Retrospective, observational  Determine relationship between BMI and risk of CVD mortality and all-cause mortality | Underweight (BMI<18·5 kg/m^2^ ) associated with a significantly increased risk of all-cause mortality (adjusted HR 0.47, 95% CI 1.21, 1.79)  Obesity (BMI=30·0–34·9 kg/m^2^_ was inversely associated with all-cause mortality in never smokers (Adjusted HR=0·71; 9 % CI 0·53, 0·97)  Obesity (BMI ≥30·0 kg/m^2^ ) associated with a heightened risk of CVD mortality in cohort without diabetes, hypertension or hypercholesterolaemia. (Adjusted HR=4.51; 95%CI 1.03, 19.71) |
|  | Chan et al., 2015.  Low physical activity and energy dense Malaysian foods are associated with non-alcoholic fatty liver disease in centrally obese but not in non-centrally obese patients with diabetes mellitus | Clinical  Patients at a diabetes clinic in an academic medical institution in Kuala Lumpur  N=299  Mean age: 63.3±10.5  Female: 59.9% | Cross-sectional, observational  Analysis between physical activity levels measured using Global Physical Activity Questionnaire, and dietary composition using a semi-quantitative food-frequency questionnaire, with anthropometry and diagnosis of non-alcoholic fatty liver disease (NAFLD) | Patients with low Physical activity were more likely to have NAFLD (OR:1.75, 95%CI: 1.03,2.99, p=0.029).  Among centrally obese patients, patients with low PA and in the highest quartile of percentage energy intake from fat (OR:4.03, 95% CI:1.12,15.0, p=0.015), high cholesterol food (OR:3.61, 95%CI: 1.37,9.72, p=0.004) and high saturated fatty acid food (OR:2.67, 95%CI: 1.08,6.67, p=0.019) were most likely to have NAFLD.  The same finding was not found in non-centrally obese patients. |
|  | Ahmad Zamri et al., 2020  Weight change and its association with cardiometabolic risk markers in overweight and obese women  (Also in B1) | Community  Overweight and obese women from low socioeconomic areas in Klang Valley  243 women (Age range 18 to 59 years) | Longitudinal, observational  Analysis of covariance (ANCOVA) to compare changes of NCD risk factors across weight change categories (2% gain, ±2% maintain, >2 to <5% loss, and 5 to 20% loss) within lifestyle intervention and control group | Within the intervention group at six months post-lifestyle intervention, participants who lost 5 to 20% of weight had the greatest improvements in NCD risk markers compared to those who did not.  -Waist circumference −5.67 cm; 95% CI: −7.98,−3.36,  -Fasting insulin level −4.27 μU/mL; 95% CI: −7.35, −1.19  -Total cholesterol level −0.59 mmol/L; 95% CI: −0.99, −0.19 |
|  | Kee et al., 2021  Association of metabolic syndrome with risk of cardiovascular disease mortality and all-cause mortality among Malaysian adults: A retrospective cohort study | Population  N= 2525 adults, aged 24–64 years, in MyNCDS-1 2005/2006, 59.9% female | Longitudinal, observational  Cox proportional hazards regression model to determine association between metabolic syndrome (MetS) and risk of CVD mortality and all-cause mortality with adjustment for sociodemographic and lifestyle behavioural factors. | Point prevalence of MetS was 30.6% (95% CI: 28.0 to 33.3).  MetS associated with a significantly increased hazard of CVD mortality (adjusted HR: 2.18; 95% CI: 1.03 ,4.61, p=0.041) and all-cause mortality (adjusted HR: 1.47; 95% CI: 1.00, 2.14, p=0.048). |
| A3. The impact of obesity on social and economic cost  n=10  Data Source:  Community: 5 Clinical: 1 Population: 3 Others: 1  Study Type:  Cross-sectional: 6 Retrospective: 2 Prospective: 1 Others: 1  Study Design:  Observational: 8 Interventional: 1 | Hassan et al., 2018  Obesity and overweight issues could undermine Malaysia's competitiveness. | Population-level BMI data from Malaysia and neighbouring Southeast Asian countries (from WHO / national databases), years 2010 & 2014 | Retrospective, observational  WHO data on average, overweight, obese prevalence categories  Comparisons across sex, country, and over time (2010 → 2014) | - Malaysia ranked second highest in prevalence of overweight and obese people in Southeast Asia (after Brunei) in both 2010 & 2014  - Percentage of overweight in Malaysia increased from 34.4 % (2010) to 38.5 % (2014)  - Obesity in Malaysia rose from 10.5 % → 13.3 % over same period  - Women had higher prevalence than men in both years  - Malaysia had highest absolute increase in overweight prevalence among the ASEAN countries studied. |
|  | Kee et al., 2017  Association of BMI with risk of CVD mortality and all-cause mortality  (Also in A2) | Population (All adult participant in NHMS 2006, followed up 5 years with mortality data from Malaysia National Registration Department)  N=32829 | Retrospective, observational  Determine relationship between BMI and risk of CVD mortality and all-cause mortality | Underweight (BMI<18·5 kg/m^2^ ) associated with a significantly increased risk of all-cause mortality (adjusted HR 0.47, 95% CI 1.21, 1.79)  Obesity (BMI=30·0–34·9 kg/m^2^_ was inversely associated with all-cause mortality in never smokers (Adjusted HR=0·71; 9 % CI 0·53, 0·97)  Obesity (BMI ≥30·0 kg/m^2^ ) associated with a heightened risk of CVD mortality in cohort without diabetes, hypertension or hypercholesterolaemia. (Adjusted HR=4.51; 95%CI 1.03, 19.71) |
|  | Moy et al., 2023  Depression and its associated factors among COVID-19 survivors in Malaysia - An online cross-sectional study. | Community  COVID-19 survivors  N=732 | Cross-sectional, observational  Online questionnaire on socio-demographic variables, comorbidities, self-perception of health, information on the person’s acute condition during COVID-19 infection, symptoms and duration of symptoms post-COVID, and mental health state (depression)  Depression was assessed using the Patient Health Questionnaire 9 (PHQ-9). | One-third of the participants perceived themselves as having poor health.  One in five participants reported having Long COVID.  Slightly less than half (47.3%) of the respondents had mild to severe depression (total PHQ-9 score of 5-27). Being female (aOR: 1.68; 95% CI: 1.08,2.62), of younger age (20s – aOR: 3.26; 95% CI: 1.47, 7.25; 30s – aOR: 2.08; 95% CI: 1.05, 4.15; and 40s – aOR: 2.43; 95% CI: 1.20, 4.90; compared to those in their 50s and above), being overweight/obese (aOR: 1.83; 95% CI: 1.18, 2.83), having Long COVID (aOR: 2.45; 95% CI: 1.45, 4.16) and perceived poorer health (aOR: 4.54; 95% CI: 2.89, 7.13) were associated with mild to severe depression. |
|  | Foo LP. Economic Analysis on the Relationship Between Medical Cost, Obesity, Health Outcomes and Labour Market in Malaysia [PhD Thesis], 2017. | Others (Clinical, population, and community)  Patient data from Serdang Hospital  Mortality data from Department of Statistics  Respondents aged 20 to 59 years old  N=391 | Others (Modelling study)  Generalized Linear Models  Prevalence and incidence data from existing literature and computed by DISMOD II  Face-to-face interviews and online questionnaire | Obese patients paid lower personal medical cost as they are covered mainly by the public hospital policies and government subsidies.  Burden of disease attributable to obesity was 2951 person-years (PYs) and 1657 PYs per 1000 persons for men and women respectively. Among all obesity related diseases worldwide, hypertension has the most significant burden of disease attributable to being obese, where men were 523 PYs per 1000 persons; this was followed by women 524 PYs per 1000 persons  Employers do not consider workers' appearance in their hiring process. However, obese workers admitted that they received unfavourable treatment during the employment |
|  | Hassan et al., 2019  Depression, anxiety and stress among obese patients with chronic illnesses: Prevalence and associated factors in Northeast Malaysia | Clinical  Obese patients with chronic medical illnesses at the Universiti Sains Malaysia Hospital outpatient clinic  N=274 | Cross-sectional, observational  21-item Depression, Anxiety and Stress Scale questionnaire | The prevalences of depression, anxiety and stress were 13.9%, 23.4% and 10.9%, respectively.  Younger age [p=0.003, adjusted odds ratio (AOR), 1.0; 95% confidence interval (CI), 0.91–0.98], unemployed employment(p=0.013, AOR,3.7; 95% CI, 1.32–10.09) and smoking (p=0.022, AOR,3.2; 95% CI, 1.18–8.55) were associated with depression.  No formal education (p=0.011, AOR,5.7; 95% CI,1.49–21.89), high body mass index (p=0.029, AOR, 1.1; 95% CI, 1.01–1.13) and family history of psychiatric illness (p=0.018, AOR,5.1; 95% CI, 1.33–19.56) were associated with anxiety.  Stress was strongly associated with females (p=0.004, AOR,5.0; 95% CI, 1.70–15.13) and smoking (p=0.002, AOR, 6.5; 95% CI, 2.03–20.7) |
|  | Mohd-Sidik et al., 2021.  Prevalence, associated factors and psychological determinants of obesity among adults in Selangor, Malaysia | Community  Adults aged ≥18 years in Selangor recruited via stratified sampling  N=1380 (62.83% Female, mean age not reported, 36.9% aged 20-29) | Cross-sectional, Observational  assessment of anthropometry and structured validated questionnaires on mental health | Being female (OR = 1.61, 95% CI [1.20–2.17]), aged between 30 to 39 years old (OR = 1.40, 95% CI [1.04–1.88]), being Indian (OR = 1.55, 95% CI [1.13–2.12]), married (OR = 1.37, 95% CI [1.03–1.83]), and having only primary school education (OR = 1.80, 95% CI [1.17–2.78] or secondary school education (OR = 1.37, 95% CI [1.04–1.81]) increased risks of being obese.  In males, higher perceived stress (B = −0.107, p = 0.041), suicidal ideation (B = −2.423, p = 0.003), and poorer physical health–related quality of life (B = −0.350, p = 0.003) were each significantly associated with lower BMI. In contrast, among females, stressful life events were positively associated with BMI (B = 0.711, p < 0.001), while poorer psychological quality of life was linked to lower BMI (B = −0.478, p < 0.001) |
|  | Wan Mohamed Radzi et al., 2020  mHealth apps assessment among postpartum women with obesity and depression. | Community  Postpartum women within one year after their pregnancy  N=819 | Prospective, interventional  Online questionnaires on the frequency of the mHealth applications usage was categorized into daily, weekly, rarely and never streams. | With the increased use of the fitness applications, there was also a significant effect in maintaining and decreasing the BMI and depression levels amongst Malaysian postpartum women.  From the data of weekly and daily use of fitness applications, the contribution toward the BMI and depression levels was high (*p* = 0.000).  Nutrition applications amongst the users were not significant within the main variables (*p* > 0.05).  The significance of using the fitness applications within the depression levels started from daily usage, whereas for BMI, it started from weekly usage. |
|  | Ng and Cheng, 2025  Weight gain, poor mental health and increased sedentary hours among Malaysian adults during the COVID-19 pandemic.  (Also in A9) | Community  Malaysian adults aged 18 and above  N=338 | Cross-sectional, observational  Online questionnaire.  Physical activity and sedentary behaviour were assessed using International Physical Activity Questionnaire-Short Form (IPAQ-SF) while the Perceived Stress Scale (PSS-10), Patient Health Questionnaire (PHQ-9) and Generalised Anxiety Disorder Assessment (GAD-7) were used to examine stress, depression and anxiety, respectively | Average weight gain of 0.6 kg among the participants, 45.5% of them experienced weight gain.  Sedentary behaviour (*p* < 0.001), PSS-10 score (*p* < 0.001), PHQ-9 score (*p* = 0.002) and GAD-7 score (*p* = 0.001) were significantly increased during the COVID-19 pandemic.  Level of physical activity was significantly decreased (*p* = 0.003) during the pandemic.  Weight changes during the pandemic were found to be associated with age, sedentary hours, and PHQ-9 score.  Sedentary hours (AOR = 1.068, 95% CI = 1.002–1.139, *p* = 0.043) were identified to be a risk factor for weight gain during the pandemic. |
|  | Khaw et al., 2023  Malaysian burden of disease: years of life lost due to premature deaths. | Population  Department of Statistics Malaysia data on life expectancy and mortality | Cross-sectional, observational  Years of life lost (YLL) method. | Non-communicable diseases caused 72.2% of total YLL. Ischaemic heart disease was the leading cause of premature mortality among Malaysians (17.7%), followed by lower respiratory infections (9.7%), road traffic injuries (8.7%), cerebrovascular disease (stroke) (8.0%), and diabetes mellitus (3.9%) |
|  | Peng et al., 2018  Effects of obesity in labour market outcomes: Evidence from Malaysia. | Community  Random sample of individuals aged 20-59 years old.  N=391 (72.1% female; mean age not reported) | Cross-sectional, observational  Face-to-face interviews and online questionnaires | BMI failed to explain differences in various income levels, but obese workers agreed that stigma does exist in Malaysia labour market.  More than half of the obese respondents claimed that they were being offered inferior jobs. The respondents felt that they were treated differently due to their body weight and some asserted that their supervisors would take into consideration their weight in delegating job assignments. |
| A4. Association of dietary intake, appetite control, eating behaviour and inflammatory status with obesity  n=33  Data Source:  Community: 28 Population: 2 Clinical: 1 Others: 2  Cross-sectional: 22 Longitudinal: 2 Observational: 22 Interventional: 2 Review: 1 | Fournier et al., 2016  Eating patterns and prevalence of obesity. Lessons learned from the Malaysian Food Barometer.  (Also in A5) | Population  Malaysians aged ≥15 years from across the country recruited via stratified random sampling  N=2000 (Mean age not reported, Female: 49.7%) | Cross-sectional, observational  analysis of associations between anthropometry with dietary habits using a quantitative survey and socioeconomic background. | Chinese had lowest mean BMI (22.88 ± 3.77) compared to Malays (23.92 ± 4.61) and Indians (24.67 ± 5.38). There was an inverse relationship between level of education and average BMI.  Overweight and obesity were not associated with higher frequency of meals per day, nor with frequency of eating out.  Obesity is over-represented in people with eating behaviour dissonance (between declared food intake than actual food intake) |
|  | Chin et al., 2022  The impact of movement control order during the COVID-19 pandemic on lifestyle behaviours and body weight changes: Findings from the MyNutriLifeCOVID-19 online survey.  (Also in A9) | Community  1,319 Malaysian adults | Cross-sectional, observational  Online survey self-reported height, weight, lifestyle behaviours (eating pattern, physical activity, sleep)  Multivariable generalized linear mixed models adjusting for age, sex, ethnicity, baseline weight status | During movement control order  -41.2% of the respondents perceived that their eating patterns were healthier  -36.3% reduced their physical activities  -25.7% had a poorer sleep quality.  -Before MCO: 54.7 % normal weight; 7.8 % underweight; 25.5 % overweight; 12.1 % obese  -During MCO: 30.7 % gained weight (mean +2.1 kg), 32.2 % lost weight (mean −2.3 kg)  -Among overweight respondents, 36.1 % lost weight, 29.4 % gained weight  Lifestyle behaviours including less frequent practice of healthy cooking methods and lunch skipping were associated with weight gain, while less frequent consumption of high fat foods, more frequent physical activity, and good sleep latency were associated with lower risk of weight gain. |
|  | Sean et al., 2024  Obesity, eating habits, and physical activity before and during COVID-19 pandemic among university lecturers.  (Also in A9) | Community  323 university lecturers from Malaysian universities | Longitudinal, observational  - Self-reported questionnaire containing sections on demographics, weight, physical activity, and eating habits changes  - Physical activity level assessed using adapted Johansson & Westerterp questionnaire  - Statistical testing for paired differences (weight changes, physical activity) and associations between weight change and behavioural factors | Baseline: 27.6 % overweight; 13.9 % obese  - During pandemic: overweight prevalence dropped to 24.8 %, obesity rose to 15.2 %  - Mean weight increased by 0.57 ± 0.56 kg during pandemic (statistically significant)  - Physical activity level decreased (from mean 1.67 ± 0.17 to 1.60 ± 0.17)  - Weight change was significantly associated with physical activity, total food intake, and increased consumption of sweetened products / confectionery, salty snacks, fast food, and sweetened drinks |
|  | Tan ST., 2022  Screen time-based sedentary behaviour, eating regulation and weight status of university students during the COVID-19 lockdown.  (Also in A7,A9) | Community  186 Malaysian private university students. | Cross-sectional, observational  - Logistic regression (multiple models) adjusting for age, lifestyle, socioeconomics, reproductive factors | Mean non-study internet use: 148 ± 77.7 min/day.  64.5% medium eating self-regulation (mean 3.0 ± 0.5).  Weight gain associated with lower eating self-regulation: β = −0.24, *p* = 0.01; ANOVA F = 5.7, *p* = 0.01 (gainers 2.9 ± 0.5 vs stable/loss 3.2 ± 0.4–0.5).  χ² = 19.9, *p* = 0.01 for association between self-regulation category and weight status.  Screen time not significantly associated with weight change. |
|  | Lim et al., 2022  Exploring the impact of COVID-19 movement control orders on eating habits and physical activity in low-resource urban settings in Malaysia.  (Also in A9) | Community  N=95  B40 Community via KOSPEN Leaders | Cross-sectional, observational  Mixed-method approach (Quantitative /qualitative) delivered via telephone interviews | There was significantly increased consumption of home-cooked meals (z = 5.099, p < 0.001).  53.7% reduced their consumption of packaged snack foods , street desserts (54.7%), fast food (50.5%), soft drinks (50.5%), and 3-in-1 or instant drinks (50.5%) due to limited access during the MCO. B40 communities were receptive to potential interventions to encourage healthier eating and physical activity leveraging digital approaches under the ‘new normal’. Reported concerns included internet accessibility and affordability, functionality, and digital literacy. |
|  | Tan et al., 2021  Trajectories of Food Choice Motives and Weight Status of Malaysian Youths during the COVID-19 Pandemic.  (Also in A9) | Community  Malaysians aged 18-30 Mean age: 22.65 ± 2.46  Female: 64.4% | Cross-sectional, observational  Self-measured anthropometry  38-item food choice questionnaire (FCQ) | 48.6% gained weight due to the confinement, with an average weight gain of 3.90 ± 2.92 kg.  47.0% to 73.0% of the youths changed their food choice motives in the time of COVID-19.  mean scores of health (t = −3.324, p = 0.001), convenience (t = −5.869, p < 0.001), weight control (t = −7.532, p < 0.001), natural content (t = −5.957, p < 0.001), ethical concern (t = −4.419, p < 0.001) and price (t = −3.737, p < 0.001) were significantly higher during the pandemic compared to pre-pandemic  Those highly concerned for weight control were more likely to be in the weight loss category (aOR) = 1.633,95%CI:1.230,2.168, p = 0.001). Conversely, those who gained weight due to the pandemic confinement highly valued natural content in foods (aOR = 0.653,95% CI = 0.481,0.886, p = 0.006) when making their food choices |
|  | Tan et al., 2022  Poor self-regulation in eating behaviour during the COVID-19 lockdown contributes to weight gain among young adults in Malaysia.  (Also in A9) | Community  Young Malaysians | Cross-sectional, observational  Self-reported weight trajectory  International Physical Activity Questionnaire-Short Form (IPAQ-SF)  Self-Regulation of Eating Behaviour Questionnaire (SREBQ) | 82.0% were reported to be physically active during the MCO, with a weekly physical activity intensity of 4551.16 ± 4744.89 MET-minute.  majority (71.0%) had medium self-regulation in eating behaviour during the lockdown, with a mean score of 3.08 ± 0.49. Slightly more than half of the young adults (57.0%) gained weight since the MCO enforcement, with an average weight increment of 1.28 ± 0.60 kg.  Young adults who attained a higher score in SREBQ were more likely in sustained weight (AOR = 2.514, p = 0.024, CI: 1.129–5.597) and weight loss (AOR = 2.624, p = 0.011, CI: 1.250–5.507) categories. |
|  | Tan & Kannan, 2023  Comparison of eating behaviour by relative weight change status of young adults throughout the COVID-19 pandemic.  (Also in A9) | Community  Diet  Adults aged 18-30 years old free from obesity-related comorbidities, and not participating in weight management interventions throughout the pandemic  N=682 (60.9% Female; Pooled mean age: 22.8±2.5 years) | Cross-sectional, observational  Compare eating behaviour by relative weight change status among young adults in Malaysia throughout the COVID-19 pandemic | 32.1% of adults put on body weight after the pandemic, with an average relative weight gain of 12.44 ± 9.67%.  weight gain category had statistically higher standardised raw scores in uncontrolled eating (49.23 ± 20.84) and emotional eating (48.66 ± 26.41) compared to those in the sustained weight category (uncontrolled eating = 44.42 ± 21.57, p = 0.039 and emotional eating = 42.81 ± 26.30, p = 0.041) |
|  | Abdul Basir et al., 2022  The challenges and strategies towards healthy eating during COVID-19 home confinement period among working adults with BMI ≥ 25 kg/m^2^ enrolled in a weight loss program: qualitative findings.  (Also in A9) | Community  Adults aged 20-59 years old with a BMI ≥ 25.0 kg/m^2^ (phone interview).  N=11 (54.5% Female, Pooled mean age: 39.6±5.4 years) | Cross-sectional, observational  Challenges and strategies for healthy diets during the first wave of the COVID-19 home confinement period among working adults | Facilitating self-control and self-efficacy in weight-related eating behaviours is imperative during the challenging pandemic-related home confinement period. |
|  | Tan et al., 2022  Changes in dietary intake patterns and weight status during the COVID-19 lockdown: a cross-sectional study focusing on young adults in Malaysia.  (Also in A9) | Community  Adults aged 18–30 years old from Malaysia (snowball sampling via social media platforms)  N=1045 (63.8% Female, Pooled mean age: 22.7±2.5 years) | Cross-sectional, observational  Changes in dietary intake patterns and their impacts on the body weight status of adults throughout the COVID-19 home confinement. | Overweight/obesity increased from 39.8% (pre-pandemic) to 41.5% (during the pandemic)  Reduced consumption in cereals and grains (β= 0.156, p < 0.001, 95% CI = 0.122–0.288), as well as oils and fats (β= 0.091, p = 0.012, 95% CI = 0.022–0.183), contributed significantly to weight loss during the pandemic confinement |
|  | Karupaiah et al., 2019.  A cross-sectional study on the dietary pattern impact on cardiovascular disease biomarkers in Malaysia | Community  Multiethnic adults aged 20 to 65 years from urban Kuala Lumpur and Petaling Jaya.  N=577 (Mean age: 38.3 ± 11·4 years, Female: 62.4%) | Cross-sectional, observational  analysis of dietary patterns with anthropometry and biomarkers of lipids, insulin resistance and inflammatory. | Dietary macronutrients showed complex associations with cardiometabolic outcomes: waist circumference was positively linked to carbohydrate and negatively to protein after adjustment, while carbohydrate intake was associated with adverse lipid markers and lower HDL-C, with protein showing a modest positive association with HDL-C. Insulin resistance markers lost significance after adjustment, glucose was inversely related to carbohydrate and positively to fat intake, and only systolic blood pressure remained inversely associated with fat in adjusted analyses.  There were no significant associations with inflammatory markers.  High-fat high-carbohydrate dietary patterns were twice as likely to have insulin resistance (aOR: 2.43,95% CI: 1·03, 5·72), and unhealthy lipid profile (aOR: 1.91, 95% CI 1.06,3.44) |
|  | Mazri FH, 2021.  Do Temporal Eating Patterns Differ in Healthy versus Unhealthy Overweight/Obese Individuals?  (Also in C2) | Community  Adult non-shift government workers from Klang Valley with BMI ≥ 25.0 kg/m2, aged 20 to 59  N=299  (Mean age: 40.3 ± 6.9, Female 73.6%) | Cross-sectional, observational  Cross-sectional analysis on relationships between dietary habits and timing, derived from a 7-day diet history questionnaire, and anthropometry and metabolic health status [metabolically healthy obesity (MHO) versus metabolically unhealthy obesity (MUO) based on standard definitions] | Adults with MUO consumed less carbohydrate and protein earlier in the day and more carbohydrate and fat later in the day compared to those with MHO. Low early energy intake was strongly associated with MUO (aOR 4.30, 95%CI 1.41,13.11), while higher late energy intake significantly increased MUO risk (ORs 2.33 to 4.45 across percentiles).  Overall, greater evening energy and carbohydrate intake and reduced morning intake were linked to higher odds of MUO. |
|  | Mohd Suki, 2024.  Association between Sleep, Stress and BMI with Chrononutrition Behaviours among Military Personnel in Malaysia | Community  Active members of Malaysian armed forces aged 18 to 50 years.  N=210  Median age: 27.5 (Interquartile range: 7), Female: 23.8% | Cross-sectional, observational  Cross-sectional assessment of relationships between sleep quality, stress level, anthropometry and chrononutrition. | Prevalence of overweight/obese was 33.3%. Sleep quality was significantly associated with evening eating (p=0.004) and night eating (p=0.028). Stress level was significantly associated with evening eating (p=0.051), night eating (p=0.019), and eating window (p=0.014).  However, there was no association between chrononutrition behaviours and BMI. |
|  | Mazri FH, 2024.  Early-Day Protein Intake Influences the Transition of Metabolic Health Phenotypes among Adults with Obesity.  (Also in C2) | Community  Adult non-shift government workers from Klang Valley with BMI ≥ 25.0 kg/m2, aged 20 to 59  N=91  Mean age: 39.6±6.3 years; Female: 74.7% | Longitudinal, interventional  Secondary data analysis of a quasi-experimental weight reduction intervention incorporating multiple lifestyle domains, including modifications in diet, physical activity, behavioural strategies, and chrono-nutrition, which encompasses temporal eating patterns, meal timing and sleep, analysing changes from between MHO and MUO status. | 24% of participants transitioned from MUO to MHO or MHO/MUO to metabolically healthy normal weight. Those who showed positive transitions were associated with: highest increase in energy intake from protein sources (+5.2% vs +1.1%, p=0.004), particularly during the earlier part of the day (+4.3% vs +1.1%, p=0.029), greater body weight loss (-7.0% vs -3.2%, p=0.022), body fat loss (-3.4% vs -1.3%, p=0.013) and visceral fat loss  (-1.3 vs -0.5, p=0.013) as well as a higher proportion of morning chronotype (72.7% vs 35.7%, p=0.033). |
|  | Mohd Fahmi Teng NI, 2023.  Chrononutrition behaviours, sugar-sweetened beverage consumption, sleep quality and the association with body mass index among Malaysian women. | Community  Adult Malay women aged from 18 to 62 across Malaysia  N=934 (Mean age: 34.3 ± 12.4 years) | Cross-sectional, observational  Analysis of chrononutrition behaviours, , sugar-sweetened beverage intake, sleep quality , collected from standardized questionnaires distributed virtually , and self-reported anthropometry | 40% of the women were overweight/obese and 65.4% reported poor sleep quality.  Breakfast skipping (OR 4.10, 95%CI 2.38,7.07), poor evening eating (aOR 4.07, 95%CI 1.63,10.19), and eating the largest meal at night (OR 6.97, 95%CI 1.94,24.99) were linked to higher odds of being underweight, while daily intake of 100% fruit juice (OR 1.67, 95%CI 1.06,1.73) and sweetened coffee/tea (aOR 1.71, 95%CI 1.16,2.51) increased odds of overweight.  Eating the largest meal at dinner also predicted poor sleep quality (aOR 2.19, 95%CI 1.12–4.30) |
|  | Juliana N, 2023.  Chrononutrition behaviour during the COVID-19 pandemic and its relationship with body weight among college students | Community  College students aged 18 to 35 years from Klang Valley.  N=409  Mean age 21.45 ± 2.2 years, Female: 89.5% | Cross-sectional, observational  analysis of chrononutrition behaviour using a standardized questionnaire, and self-reported anthropometry. | Prevalence of underweight was 24.7%, and overweight was 25.9%. There were significant associations between underweight BMI and poor eating behaviours in terms of eating window, evening latency, evening eating, and night eating. |
|  | Say YH, 2024.  Cross-sectional association study of hedonic hunger, self-control, cognitive distortion, and well-being with adiposity measures among a sample of urban Malaysian adults. | Community  Urban Malaysian adults aged 18 to 80 years in urban Selangor.  N=186  Mean age: 22.09 ± 5.04 years  Female: 72.6% | Cross-sectional, observational  Analysis associations between four psychometric measures – hedonic hunger, self-control (impulsivity and restraint), cognitive distortion (CD), and well-being, with anthropometric and body composition measures. | Greater hedonic hunger and cue-reactive eating were linked to elevated systolic blood pressure and increased adiposity across all measures. Individuals with overweight showed stronger hedonic responses when highly palatable foods were readily available or physically present. Weak associations were observed between lower self-control and higher central adiposity, while reduced impulsivity and restraint were modestly related to greater visceral fat. |
|  | Muhammad R, Ismail 2023.  Intuitive Eating Behaviour among Young Malay Adults in Malaysian Higher Learning Institutions. | Community  Students from higher learning institutions across Malaysia  N=367 (Mean age: 22.17 ± 1.59, Females: 83.9%) | Cross-sectional, observational  analysis of intuitive eating practices with weight-control behaviours and binge eating using standard questionnaires [Intuitive Eating Scale-2 (IES-2) and Diabetes Eating Problems Survey (DEPS)]. | There were similar overall intuitive-eating scores by sex (IES-2: men 3.52±0.32, women 3.47±0.35; p=0.147), but men scored higher on Eating for Physical rather than Emotional Reasons and Body–Food Choice Congruence (both p<0.05); among women, intuitive-eating responses varied by living area (p<0.05).  Weight-related behaviours were common: 9.8% of men and 52.3% of women were currently dieting; 76.3% men and 80.7% women had unhealthy weight-control behaviours; 81.4% men and 84.7% women had binge eating behaviours  Intuitive eating was not associated with lower BMI. Dieting was explained in part by weight-control behaviour and binge eating (R²=0.34), |
|  | Tengku Ismail TA, 2018.  Understanding Dieting and Previous Weight Loss Attempts among Overweight and Obese Participants: Insights into My Body Is Fit and Fabulous at Work Program | Community  Participants in a weight-loss intervention program at work in Kota Bharu, Kelantan.  N=129 (Mean age: 43.5±9.22, Females: 60%) | Cross-sectional, observational  In-depth interviews using focus group discussions and thematic analysis on weight loss attempts and body image. | Perception of dieting were various, including skipping meals and removing certain food groups such as rice from daily diets.  Weight loss strategies employed include lifestyle, pharmacological (both traditional and modern medicine), and over=the-counter products. Outcomes of attempts were variable, with weight regain being common.  Health and appearance, social support and compliance with current trends were motivation factors for weight loss.  Weight loss attempts were limited by lack of motivation, unpleasant side effects, lack of initial positive outcomes, inconducive social environments. |
|  | Ganesrau G, Ali 2023.  Ultra-Processed Food Consumption About Body Mass Index (BMI) of Public University Students in Malaysia. | Community  Public university students aged ≥18 in four states (Kedah, Terengganu, Johor and Selangor) and Kuala Lumpur.  N=250  Median age: 22, Interquartile range:1; Female: 76.8% | Cross-sectional, observational  analysis of ultra-processed food consumption derived from 24-Hour dietary recall with anthropometry. | There was a weakly positive correlation between ultra-processed food intake with BMI (r=0.16, p=0.014) |
|  | Yap 2019.  Poor Diet Quality among Overweight/Obese (OW/OB) Young Adults in Klang Valley, Malaysia: A Case–control Study | Community  Private university students aged 18 to 25 in Klang Valley.  N=294 (females: 53.7%) | Cross-sectional, observational  analysis of dietary quality assessed using healthy eating index, derived from 24-hour dietary recall, with anthropometry, and body composition. | Overweight/obese groups had significantly higher energy, macronutrient, sugar, saturated fat, cholesterol and sodium intake compared to normal weight group.  Healthy eating index score was lower in overweight/obese group compared to normal weight group (45.14±11.13 versus 51.43±11.61).  Both overweight/obese group and normal weight group had inadequate consumption of fruits, vegetables and milk. |
|  | Kaur, 2022.  Identifying Dietary Pattern Associated with Adiposity among Malaysian Young Adults. | Community  Private university students aged 18 to 25 in Klang Valley.  N=294 (females: 53.7%) | Cross-sectional, observational  Analysis of dietary quality assessed using healthy eating index, derived from 24-hour dietary recall, with anthropometry, and body composition | Youths consumed 214 (247) kcal/day of ultra-processed foods and exceeded recommended sugar [32.43 (41.20) g] and sodium [2425.26 (1455.18) mg] intakes, while fruit (0.29 ± 0.72), vegetable (0.95 ± 0.77), and milk (0.24 ± 0.38) servings/day were below guidelines. Higher meat and sodium intake correlated with increased BMI, waist circumference, body fat, and visceral fat (p<0.001), while ultra-processed food intake was linked to elevated BMI (p=0.009), waist circumference (p=0.046), and visceral fat (p=0.014); high sugar intake was similarly associated with greater BMI (p=0.003), body fat (p=0.020), and visceral fat (p=0.027) |
|  | Chen AB, 2023.  Recent research patterns and factors influencing eating behaviour amongst Malaysian youths: a scoping review | Others  Research reports focusing on dietary patterns among Malaysian youths published between 1st January 2017 to 31 December 2022  N(studies)=34 | Review  Scoping review of relevant studies and identification of themes | Most studies on eating behaviour among Malaysian youths has mainly focused on institutional and urban settings, with limited work in East Malaysia.  Most research focused on intrapersonal factors such as knowledge, attitudes, perceptions, and body image, while fewer studies examined interpersonal and environmental influences.  Although youths generally demonstrated good knowledge of healthy eating, actual practices often contradicted this, highlighting the need for more qualitative research and broader attention to socio-cultural, ethnic, and environmental determinants of eating behaviour. |
|  | Johari SM 2017.  High body fat percentage among adult women in Malaysia: the role of lifestyle | Community  Female participants of a health screening program in urban central Malaysia.  N=327  Mean age 50.4 ± 5.6 years | Cross-sectional, observational  analysis of data on body composition using bioimpedance analysis, dietary and physical activity | Lower protein intake (B=-0.018, p=0.001), Higher calcium intake (B=0.001, p=0.010), and lower levels of household-level intensity physical activity (B= -4.566E-5, p=0.021) were associated with higher body fat percentage. |
|  | Mohd Hasni, 2023.  New insights of minimum requirement on legumes (Fabaceae sp.) daily intake in Malaysia | Community  Adults aged 35 to 70 years from various urban and rural areas in Malaysia.  Mean age: 54.80 ± 8.76, Females: 55.78% | Cross-sectional, observational  analysis of anthropometry, comorbidities, and legume intake measured using standardized questionnaires. | People who consumed ≥3 servings of legumes per day were protected against cardiovascular risks (OR:0.56 95%CI 0.37,0.85), especially those with body mass index ranking 26 to 34 kg/m^2^. |
|  | Lee YY, 2019.  Dietary intakes and obesity of Malaysian adults. | Community  Adults aged 20 to 65 years from Penang and Kota Bharu.  N=490  Mean age:40.0 ± 14.3  Female: 64.7% | Cross-sectional, observational  analysis of anthropometry and diet history via 3-day 24-Hour dietary recall. | Mean energy intake was 1550 kcal/day, males had higher energy and macronutrient intake than females.  Protein consumption and percentage of energy contribution exceeded recommended range.  Consumption of fruits, vegetables, and dairy were lower than recommended.  Carbohydrate intake was negatively associated with BMI (Estimate *b* = −0.008) and waist circumference (Estimate *b*=-0.019). |
|  | Tan PY 2021.  Dietary copper and selenium are associated with insulin resistance in overweight and obese Malaysian adults | Community  Nondiabetic Malaysian adults aged ≥18 years with BMI ≥23 kg/m^2^ in Selangor  N=128  Mean age: 44.0 ± 10.9  Female: 84.4% | Cross-sectional, observational  analysis of dietary content of copper and selenium via food frequency questionnaires and insulin resistance measured using homeostatic model assessment-insulin resistance (HOMA-IR) | There was a U-shaped non-linear relation between Selenium intake and insulin resistance, with critical points at <0.3 µg/kg/day and ≥1.01 µg/kg/day.  There was significant positive association with dietary copper and insulin resistance at intake levels of copper ≥ 13.4 µg/kg/day. |
|  | Chang LF, 2016.  Adverse effects on insulin secretion of replacing saturated fat with refined carbohydrate but not with monounsaturated fat: A randomized controlled trial in centrally obese subjects | Community  Adults aged 20-60 years with abdominal obesity.  N=47  Mean age 32.8 ± 8.7, females: 74.5% | Longitudinal, interventional  Randomized controlled crossover trial of substitution of 7% of total energy intake as carbohydrate or monounsaturated fatty acids for saturated fatty acids for 6 weeks, and its effects on glucose tolerance and insulin resistance. | Lower-fat, higher carbohydrate diet attenuated postprandial insulin secretion. Saturated fatty acids and monounsaturated fatty acids had similar effects on all markers, but glucose-dependent insulinotropic peptide increased after monounsaturated fatty acid intake. |
|  | Shyam S, 2020.  Association between Dietary Patterns and Overweight Risk among Malaysian Adults: Evidence from Nationally Representative Surveys. | Population  Nationally representative sample of Malaysian adults from two iterations of Malaysian Adult Nutrition Surveys  N (2003)=6928 N (2014)=3000). | Cross-sectional, observational  analysis of dietary patterns derived from food frequency questionnaires with prevalence of overweight | Different dietary patterns (‘Traditional’, ‘Western’, ‘Mixed’, ‘Flatbread & beverages’, and ‘Noodles & Meat’) were associated with different sociodemographic profiles.  Overweight could not be attributed to adherence to a single dietary pattern |
|  | Eng JY, 2020.  Dose–Response Relationship between Western Diet and Being Overweight among Teachers in Malaysia. | Community  Female teachers from multi-stage sampling across three states from 2014 to 2015  N=6472 (Mean age: 40.3±0.1 years, Female: 82.6%) | Cross-sectional, observational  analysis of two dietary patterns (Western diet: high in whole grains, vegetables, fruits, legumes, pulses, low-fat dairy, poultry, and fish, versus Prudent diet: high in whole grains, vegetables, fruits, legumes, pulses, low-fat dairy, poultry, and fish) derived from food frequency questionnaires | Higher adherence to a Western dietary pattern was linearly associated with increased odds of overweight and obesity, with participants in the highest quintile of Western diet score being more likely (aOR: 1.4 95% CI: 1.11, 1.83, p-trend <0.001) to be overweight/obese than those in the lowest quintile.  In contrast, greater adherence to a Prudent diet was inversely associated (p-trend<0.001) with overweight/obesity in men |
|  | Chan WK, 2015.  Low physical activity and energy dense Malaysian foods are associated with non-alcoholic fatty liver disease in centrally obese but not in non-centrally obese patients with diabetes mellitus  (Also in A7) | Clinical  Patients at a diabetes clinic in an academic medical institution in Kuala Lumpur  N=299  Mean age: 63.3±10.5  Female: 59.9% | Cross-sectional, observational  analysis between physical activity levels measured using Global Physical Activity Questionnaire, and dietary composition using a semi-quantitative food-frequency questionnaire, with anthropometry and diagnosis of non-alcoholic fatty liver disease (NAFLD) | Patients with low Physical activity were more likely to have NAFLD (OR:1.75, 95%CI: 1.03,2.99, p=0.029).  Among centrally obese patients, patients with low PA and in the highest quartile of percentage energy intake from fat (OR:4.03, 95% CI:1.12,15.0, p=0.015), high cholesterol food (OR:3.61, 95%CI: 1.37,9.72, p=0.004) and high saturated fatty acid food (OR:2.67, 95%CI: 1.08,6.67, p=0.019) were most likely to have NAFLD.  The same finding was not found in non-centrally obese patients. |
|  | Mat Ludin 2024.  Association between Knowledge, Attitude, and Practice on Dietary Fiber Intake and Body Mass Index among Undergraduate Students in Universiti Kebangsaan Malaysia. | Community  Dentistry and Software Engineering with Information System Development undergraduate students at a public university in Selangor.  N=183 (Mean age not reported, females:39.3%) | Cross-sectional, observational  analysis of knowledge, attitudes and practices on dietary fibre intake and anthropometry. | Levels of knowledge, attitudes and practices on fibre intake were moderate across both groups.  The knowledge, attitude and practice on fibre intake did not influence BMI in both studied groups. |
|  | Brian CA, Stephenson ML, Tan AL. Recent research patterns and factors influencing eating behaviour amongst Malaysian youths: a scoping review. Frontiers In Sustainable Food Systems. 2023;7. | Others  Studies published on eating behaviour amongst Malaysian youths from 2017 to 2022  N=34 studies | Scoping review | Majority of the recent studies focused on the general adequacy and frequency of consumption of food groups studied using quantitative approaches across multi-ethnic target groups within the central region of Malaysia  More studies on the consumption of specific food groups, eating disorders and weight control that could be more ethnic-centric within other geographical areas within Malaysia are needed |
| A5. Determination of socio-cultural factors influencing obesity  n: 19  Data source:  Community: 13 Population: 5 Clinical: 1  Study Type  Cross-sectional: 16 Prospective: 0 Retrospective: 2 Others: 1  Study Design  Observational: 18 Review: 1 | Baharudin A, et al. 2017  Changes in Nutritional Status among Malaysian Adults Population from 2003 to 2014. | Population  Participants in MANS 2003 and 2014  N=4044 | Retrospective, observational | The mean body mass index for adult Malaysians increased from 24.4 (95% CI: 24.2-24.5) in Malaysian Adult Nutrition Survey (MANS) 2003 to 25.6 (95% CI: 25.3-25.9) in MANS 2014. The prevalence of overweight and obesity was 32.4% (95% CI: 30.29-34.59) and 18.5% (95% CI: 16.6-20.5) respectively, increases since MANS 2003 when the prevalence was 26.71% (95% CI: 25.5-27.96) and 12.15% (95% CI: 11.26-13.1), respectively |
|  | Raman S et al. 2023  Application of Health Belief Model: Demographic Factors Affecting Body Mass Index (BMI) and Perceptions of Weight Management among Malaysians.  . | Community  Adults aged 18 and above  N=444  Mean age not reported Majority (41.8% aged 18-30) Female 56.6% | Cross-sectional, observational  Self-measured anthropometry  Health Belief Model Questionnaire (HBMQ). | Significant associations were observed between body mass index (BMI) and demographic characteristics such as age range, gender, education level, marital status, employment status, intention to reduce weight, and health issues (P < 0.05)  perceived severity and behavioural intention were the significant variables to predict a person’s BMI. |
|  |  |  |  |  |
|  | Mariapun, 2018.  The Gradual Shift of Overweight, Obesity, and Abdominal Obesity Towards the Poor in a Multi-ethnic Developing Country: Findings from the Malaysian National Health and Morbidity Surveys | Population  Serial population-based National Health and Morbidity Survey Censuses for 1996,2006 and 2011 among adults aged≥30 years  N, (% Female):  1996: 20,34; (51.5%) 2006: 22,693; (54.3%) 2011: 11,989; (53.5%)  Mean age not reported | Retrospective, observational  Assessment of trends of overweight/obesity and abdominal obesity prevalence over time among different socioeconomic strata | Women in Peninsula Malaysia showed a trend of distribution of overweight, obesity and abdominal obesity prevalence towards the poor over time.  For men in East Malaysia, distributions were concentrated in the rich.  Chinese women in the high socioeconomic strata had lowest increment in rates. |
|  | Aghakhanian F, 2019.  Metabolic syndrome and cardiometabolic risk factors among indigenous Malaysians.  (Also in A2,A9) | Community  People from Indigenous communities across peninsula Malaysia aged >18  N=629 (Females 41.8%) | Cross-sectional, observational  analysis of anthropometry, plasma glycaemic and lipid profiles | Prevalence of obesity was 52.1% (95%CI 44.9, 59.3), and abdominal obesity was 72.6% (95%CI 66.1,79.2). Metabolic syndrome affected 29.57% of the indigenous people, being more prevalent in females than males (35.25% versus 21.95%, p<0.001). |
|  | Zainuddin AA, et al. 2016  Prevalence and Socio-Demographic Determinant of Overweight and Obesity among Malaysian Adult.  (Also in A6) | Population  NHMS 2015  N=17,257 | Cross-sectional, observational | Risk factors associated with obesity include: Indian (aOR: 1.8, 95%CI: 1.2-2.8), aged 50-59 years (aOR: 2.8, 95%CI: 2.0-3.9), widower (aOR: 1.6, 95%CI: 1.3-2.0), subject with secondary education (aOR: 1.2, 95%CI: 1.0-1.4), Homemaker/unpaid worker (aOR: 1.3, 95%CI: 1.1-1.4), and with high household income group (aOR: 1.3, 95%CI: 1.2-1.6). The identified risk of obesity were women (aOR: 1.4, 95%CI: 1.2-1.6), Indian (aOR: 1.7, 95%CI: 0.9-3.2), aged 30-39 years (aOR: 3.6, 95%CI: 2.4-5.5), widower (aOR: 1.2, 95%CI: 0.9-1.6), subjects with primary education (aOR: 1.2, 95%CI: 0.9-1.6), Homemaker/unpaid worker (aOR: 1.3, 95%CI: 1.1-1.6), and with middle household income group (aOR: 1.3, 95%CI: 1.2-1.6) |
|  | Kyaw et al. 2022  Obesity and its associated factors among older adults: MyHEBAT (Malaysian HEalth and Well-Being AssessmenT) study  (Also in A6) | Community  Older Adults aged >= 60 years who attended health screening in most states in East and West Malaysia  N=716  (54.5% females, mean age 66.6 ± 6.0 years) | Cross-sectional, observational  Regression analysis of factors associated with obesity (BMI≥30) | Factors associated with obesity: aged 70-79 years old (compared to 60-69 years old), female, non‐Malay, with tertiary level education, monthly income ≥RM3000, has diabetes, hypertension, family history of hypertension, and a family history of CVD  (Effect size not included because of multiple interactions – refer main text) |
|  | Iqbal SP, 2020.  Relationship of sociodemographic and lifestyle factors and diet habits with metabolic syndrome (MetS) among three ethnic groups of the Malaysian population | Community  Residents of Johor aged ≥18 years  N=481  62.0% with abdominal obesity Mean age and BMI not reported | Cross-sectional, observational  study among attendees of a medical camp | Indians had highest rates of metabolic syndrome (51.9%). Indians had higher odds of metabolic syndrome (aOR: 1.95 95%CI 1.12-3.38). Among Malays and Chinese, quick finishing of meals (aOR: 0.44, 95%CI 1.02-4.60), and low physical activity (aOR: 4.76, 95%CI: 1.49-15.26) increased odds of metabolic syndrome. |
|  | Sugathan S, 2021.  Socio demographic and lifestyle factors of metabolic syndrome among adult rural indigenous Malaysian population from Perak State, Malaysia | Community  People from four rural indigenous villages in Perak aged ≥18 years  N=293 (Mean age not reported, majority (62.5%) were <45 years old, females: 66.2%) | Cross-sectional, observational  analysis with comparison between rural Malays and indigenous Malaysians (*Orang Asli),* in terms of sociodemographic and lifestyle factors with metabolic syndrome (abdominal obesity, dyslipidaemia, high blood pressure and high fasting glucose) | Prevalence of metabolic syndrome was 20.8%. The prevalence was higher among those who were obese and overweight, and among Malays who regularly snacked between meals.  There was no association between cigarette smoking, alcohol use or physical activity with Metabolic syndrome. |
|  | Fournier T, 2016.  Eating patterns and prevalence of obesity. Lessons learned from the Malaysian Food Barometer.  (Also in A4) | Population  Malaysians aged ≥15 years from across the country recruited via stratified random sampling  N=2000 (Mean age not reported, Female: 49.7%) | Cross-sectional, observational  analysis of associations between anthropometry with dietary habits using a quantitative survey and socioeconomic background. | Chinese had lowest mean BMI (22.88 ± 3.77) compared to Malays (23.92 ± 4.61) and Indians (24.67 ± 5.38). There was an inverse relationship between level of education and average BMI.  Overweight and obesity were not associated with higher frequency of meals per day, nor with frequency of eating out.  Obesity is over-represented in people with eating behaviour dissonance (between declared food intake than actual food intake) |
|  | Ramadas, 2021.  Diet Quality of Malaysians across Lifespan: A Scoping Review of Evidence in a Multi-Ethnic Population | Population  Studies on dietary quality, influencing factors, and health outcomes across the lifespan of Malaysians.  N(Studies)=29 | Scoping review of relevant studies | Overall, diet quality was poor in all age groups.  The intake of fruit, vegetable, legumes, and dairy intake were generally deficient. Meat, salt, and sugar intake were reportedly excessive in many studies |
|  | Kocher E, 2022.  Community Priorities for Obesity Prevention among Low-Income Adults in Kuala Lumpur: A Discrete Choice Experiment | community  Participants across Malaysia recruited using online social media portals  N=440 | Cross-sectional, observational  Survey using self-administered Health Belief Model Questionnaire (HBMQ). | Significant associations were observed between body mass index (BMI) and demographic characteristics such as age range, gender, education level, marital status, employment status, intention to reduce weight, and health issues (P < 0.05). A statistically significant difference was observed between HBM subscales and different demographic characteristics. The regression model explained ~8.3% of the variance in BMI (P < 0.001) and revealed that perceived severity and behavioural intention were the significant variables to predict a person’s BMI. |
|  | Abdullah Z,2022.  Contrariwise obesity through organic food consumption in Malaysia: a signalling theory perspective  (Also in B3) | Community  Online survey from social media websites of Malaysian communities  N= 241 | Cross-sectional, observational | Health consciousness not significantly predicts the purchase intention of healthy food. All antecedents were significant contributors to the prediction of foods’ purchase intentions in this study. However, the findings indicated that no positive relationship exists between brand image identifications and brand credibility identifications, and healthy foods’ purchase intentions identifications. |
|  | Omar R, 2023.  Prevalence Of Non-Communicable Disease Risk Factors Among Healthcare Workers in The KOSPEN WOW Program in Kedah. | Community  All healthcare workers under public health facilities that implemented the Healthy Community Builds the Nation – Wellness of Workers (KOSPEN-WOW) program by Ministry of Health Malaysia from 2020-2022.  N=8603 (2020), 5600 (2021), 10967 (2022)  Mean age and sex distribution not reported | Cross-sectional, observational  Serial assessment of trends of noncommunicable disease and risk factors among healthcare workers from 2020 to 2022 | Rates of obesity (BMI > 25 kg/m2) increased from 45.4% (2020) to 54.5% (2021) and reduced to 48.6% (2022), and rates of abdominal obesity increased from 29.7% (2020) to 40.3% (2021) and 41% (2022).  The prevalence of anxiety was highest (10.6% to 13.8%) compared to stress (7.0% to 7.5%) or depression (5.9% to 7.8%). |
|  | Kunyahamu MS, 2021.  Obesity among Health-Care Workers: Which Occupations Are at Higher Risk of Being Obese? | Community  Healthcare workers who participated in a health screening program in 2019.  N=4241 (Mean age: 35.64 +/- 7.37, Females 75.3%) | Cross-sectional, observational  analysis of secondary data | Prevalence of overweight was 21.1% and obesity was 33.1%.  Nurses had higher risk of being obese (aOR: 1.91, 95%CI 1.45,2.53). |
|  | Hng SH 2019.  Eating Behaviour and Its Associations with Overweight among Nurses on Shift Duty in Teaching Hospital. | Community  Nurses from an academic teaching hospital in Kuala Lumpur.  N=308 (Mean age: 33.62+/- 5.49 | Cross-sectional, observational  assessment of anthropometry and eating patterns among nurses on shift duty. | Prevalence of overweight was 37.1% and obesity was 31.4%.  Restraint eating behaviour was associated with higher likelihood of obesity (F=6.056, p=0.003) |
|  | Lim YC, 2018.  Association Between Night-Shift Work, Sleep Quality and Metabolic Syndrome. | Community  Manufacturing workers from 11 factories in Klang Valley as part of Social Security Organization’s (SOCSO) Health Screening Programme  N=494 (Mean age: 46.8±5.3, Female: 48.8%) | Cross-sectional, observational  analysis of work patterns, sleep patterns and biomarkers of metabolic health | Prevalence of abdominal obesity: 52.6%, higher in night-shift workers. Night-shift work was independently associated with a twofold increase in the risk of metabolic syndrome (aOR: 1.92, 95%CI: 1.24,2.97), regardless of sex. Night-shift workers also reported significantly poorer sleep quality, longer sleep latency, shorter sleep duration, sleep disturbances and daytime dysfunction. |
|  | Dadar Singh, 2022.  Obesity and Mental Health Issues among Healthcare Workers: A Cross-Sectional Study in Sabah. | Community  Healthcare workers in Kota Kinabalu District Health Office, Sabah in 2019  N=387 (Mean age not reported, Female: 78.2%) | Cross-sectional, observational  analysis of anthropometry and measures of mental health | Prevalence of obesity was 29%. Duration of service of more than 5 years (aOR 2.23, 95%CI 1.16,4.28), high depression scores (aOR 2.09, 95%CI 1.18,3.71) were independently associated with obesity. |
|  | Wan AH, 2020.  Barriers in Adopting Healthy Body Weight Among Malaysian Population: A Cross-Sectional Study of Body Weight Perception and Misperception Versus Actual Body Weight | Clinical  Mean age not reported,  Majority (59.1%) aged between 25 and 54  Female: 59.4% | Cross-sectional, observational  Self-administered questionnaire consisting of the sociodemographic profile, body weight perception question adopted from Weight Management Questionnaire and Youth Risk Behaviour Surveillance System (YRBSS) 1991-2017 | The prevalence of body weight misperception in the sample population was 58.6%. Most of the respondents were overweight (33.9%) and obese (33.9%). Respondents aged 25 years and older were 2.98 times more likely to have misperception compared with other age groups (95% CI: 1.21-3.19, P = .006). Divorced respondents were 4.70 times more likely to have body misperception compared with married respondents (95% CI 1.44-15.32, P = 0.01). |
|  | Yunus NA, 2022.  What is it like to live with obesity in Peninsular Malaysia? A qualitative study | Community  Adults with obesity in Malaysia  N=25 (Mean age not reported:92% Female)  (Mean age | Cross-sectional, observational  Qualitative study with in-depth teleconference interviews, and identification of themes using reflexive thematic analysis approach. | Five themes were identified:   1. Malaysian life is centred around food 2. Social norms shape people living with obesity's perceptions of themselves and obesity 3. People living with obesity are physically restricted by their body 4. People living with obesity have repeated thoughts about efforts to lose weight 5. Stigmatization of people living with obesity leads to negative emotions |
| A6. Effect of metabolic predisposition to onset of obesity.  n=8  Data Source:  Community: 5 Population: 3  Study Design:  Cross-sectional: 8 Observational: 8 | Chong et al. 2023  Prevalence of overweight and its associated factors among Malaysian adults: Findings from a nationally representative survey  (Also in A2) | Population  Adults from National Health and Morbidity Survey 2019 (nationally representative sample)  N=9782 | Cross-sectional, observational  Regression analysis of factors associated with obesity | Female gender [aOR (95% CI) = 1.33 (1.11, 1.58); p = .002], ages 30–59 years [aOR (95% CI) = 1.61 (1.31, 1.97); p < .001], being Malay [aOR (95% CI) = 1.68 (1.36, 2.07); p < .001], Indian [aOR (95% CI) = 2.59 (1.80, 3.74); p < .001] or other Bumiputera [aOR (95% CI) = 1.82 (1.38, 2.39); p < .001], being married [aOR (95% CI) = 1.23 (1.00, 1.50); p = .046], and having adequate health literacy [aOR (95% CI) = 1.19 (1.01, 1.39); p = .033] |
|  | Cheah WL, 2016.  Gender and Racial Differences in the Cardiovascular Risk Factors among Overweight and Obese Rural Adults, Kuching and Samarahan Division, Sarawak, Malaysia | Members of two rural communities in Sarawak  N=155 (mean age: 45.1 +/- 10.23, Females: 78.06%) | Cross-sectional observational  analysis of anthropometry, body composition, and cardiovascular risk estimated by atherogenic index of plasma (AIP) | Compared to Malays, Bidayuh people had higher odds of having higher body fat percentage (OR:4.9, 95%CI 1.22,7.89), and lower levels of AIP-estimated cardiovascular risk (OR: 0.30, 95%CI 0.12,0.78), whereas Iban people had higher odds for hyperglycaemia (OR: 3.86, 95%CI 1.06, 6.66), but lower AIP (OR 0.29, 95%CI 0.12,0.69)., adjusted for age and sex. |
|  | Aghakhanian F, 2019.  Metabolic syndrome and cardiometabolic risk factors among indigenous Malaysians.  (Also in A5, A9) | People from Indigenous communities across peninsula Malaysia aged >18  N=629 (Females 41.8%) | Cross-sectional, observational  analysis of anthropometry, plasma glycaemic and lipid profiles | Prevalence of obesity was 52.1% (95%CI 44.9, 59.3), and abdominal obesity was 72.6% (95%CI 66.1,79.2). Metabolic syndrome affected 29.57% of the indigenous people, being more prevalent in females than males (35.25% versus 21.95%, p<0.001). |
|  | Zainuddin AA, , et al. 2016  Prevalence and Socio-Demographic Determinant of Overweight and Obesity among Malaysian Adult.  (Also in A5) | Population  NHMS 2015  N=17,257 | Cross-sectional, observational | Risk factors associated with obesity include: Indian (aOR: 1.8, 95%CI: 1.2-2.8), aged 50-59 years (aOR: 2.8, 95%CI: 2.0-3.9), widower (aOR: 1.6, 95%CI: 1.3-2.0), subject with secondary education (aOR: 1.2, 95%CI: 1.0-1.4), Homemaker/unpaid worker (aOR: 1.3, 95%CI: 1.1-1.4), and with high household income group (aOR: 1.3, 95%CI: 1.2-1.6). The identified risk of obesity were women (aOR: 1.4, 95%CI: 1.2-1.6), Indian (aOR: 1.7, 95%CI: 0.9-3.2), aged 30-39 years (aOR: 3.6, 95%CI: 2.4-5.5), widower (aOR: 1.2, 95%CI: 0.9-1.6), subjects with primary education (aOR: 1.2, 95%CI: 0.9-1.6), Homemaker/unpaid worker (aOR: 1.3, 95%CI: 1.1-1.6), and with middle household income group (aOR: 1.3, 95%CI: 1.2-1.6) |
|  | Kyaw et al. 2022  Obesity and its associated factors among older adults: MyHEBAT (Malaysian HEalth and Well-Being AssessmenT) study  (Also in A5) | Community  Older Adults aged >= 60 years who attended health screening in most states in East and West Malaysia  N=716  (54.5% females, mean age 66.6 ± 6.0 years) | Cross-sectional, observational  Regression analysis of factors associated with obesity (BMI≥30) | Factors associated with obesity: aged 70-79 years old (compared to 60-69 years old), female, non‐Malay, with tertiary level education, monthly income ≥RM3000, has diabetes, hypertension, family history of hypertension, and a family history of CVD  (Effect size not included because of multiple interactions – refer main text) |
|  | Chong ETJ, 2018.  Prevalence of Overweight and Obesity in Malaysia, 2010-2016: A Comprehensive Meta-Analysis. | Community  Secondary data  N=20 751 | Cross-sectional, observational  Meta-analysis of 18 observational studies published in PubMed from 2010 to 2016. | Overall prevalence rate of overweight was 25.0%, 5% lower than NHMS 2015 data.  Stratifying by gender, there is no difference in the prevalence of overweight between females (25.2%) and males (25.1%), but prevalence of obesity is higher in females (15.3%) compared to males (12.6%) |
|  | Apalasamy YD, 2021.  Factors Associated with Obesity and Abdominal Obesity Among Malaysian Older Adults | Community  Malaysians aged ≥ 40 years across the country, recruited via stratified sampling as part of the Malaysian Ageing and Retirement Survey (MARS)  N=5613 | Cross-sectional, observational  analysis of a planned longitudinal study, data collected using computer-assisted personal interviewing, including detailed demographics, healthcare utilization, self-perception of health, and physical activity levels.  Regression analysis for factors associated with obesity and abdominal obesity | Females had higher risk of being obese (OR: 1.64, 95%CI: 1.45,1.86),  People aged 40-49 years old had highest odds of being obese (OR: 2.58, 95%CI 2.06,3.23) compared to those aged 70 or above.  Ethnicities that had higher odds of being obese included Indians (OR:1.69, 95% CI 1.34-2.12), Malays (OR: 1.40, 95%CI 1.22,1.64), whereas Chinese had lower odds (OR: 0.63,95% CI 0.50,0.79), compared to other natives.  Income of RM2000 or higher were more likely to be obese (OR: 1.22, 95%CI 1.05-1.43) |
|  | Ariaratnam S,2020.  Prevalence of obesity and its associated risk factors among the elderly in Malaysia: Findings from The National Health and Morbidity Survey (NHMS) 2015. | Population  Census data from National Health and Morbidity Survey 2015, among elderly population aged ≥ 60 years  N=3794 (Mean age not reported, Female: 52.9%) | Cross-sectional, observational  analysis for risk factors of obesity | Elderly females (aOR: 1.52, 95%CI 1.28,1.81), having secondary education (aOR 1.96, 95% CI 1.51,2.54), those with household income of RM 3000 and above (aOR 1.57, 95%CI: 1.29,1.91) as well as being hypertensive (aOR 1.61, 95%CI: 1.35,1.93) and diabetic (aOR 1.50, 95%CI 1.28.1.76) were more likely to be obese. Current smokers (aOR 059, 95%CI 0.45,0,77) and those who were more physically active (aOR 0.78, 95%CI 0.67,0.91) were less likely to be obese. |
| A7. Association between physical inactivity, sedentary lifestyle and obesity.  n=17  Data Source:  Community: 11 Population: 5 Clinical: 1  Study Type:  Cross-sectional: 15 Retrospective: 2  Study Design:  Observational: 16 Modelling: 1 | Ng GF 2025  Weight gain, poor mental health and increased sedentary hours among Malaysian adults during the COVID-19 pandemic | Community  Malaysians aged 18 and above recruited via online social media portals  N=338  33.7 ± 14.0  Female: 68.6% | Cross-sectional, observational  Questionnaire delivered via online  International Physical Activity Questionnaire-Short Form (IPAQ-SF) while the Perceived Stress Scale (PSS-10), Patient Health Questionnaire (PHQ-9) an & Generalised Anxiety Disorder Assessment (GAD-7)  Self-measured anthropometry | Average weight gain of 0.6 kg among the participants with 45.5% of them experiencing weight gain.  Sedentary behaviour (p < 0.001), PSS-10 score (p < 0.001), PHQ-9 score (p = 0.002) and GAD-7 score (p = 0.001) were significantly increased during the COVID-19 pandemic  Level of physical activity was significantly decreased (p = 0.003) during the pandemic. Weight changes during the pandemic were found to be associated with age, sedentary hours, and PHQ-9 score.  Sedentary hours (AOR = 1.068, 95% CI = 1.002–1.139, p = 0.043) were identified to be a risk factor for weight gain during the pandemic. |
|  | Tan ST 2022  Screen time-based sedentary behaviour, eating regulation and weight status of university students during the COVID-19 lockdown.  (Also in A4,A9) | Community  Private university students  N=186 | Cross-sectional, observational  Self-reported anthropometry  HELENA sedentary behaviour questionnaire:  Self-Regulation of Eating Behaviour Questionnaire (SREBQ) | Respondents spent most of the time on the internet for non-study purposes (148 ± 77.7 min).  64.5% of the respondents had medium eating self-regulatory skill during the MCO, with an average score of 3.0 ± 0.5.  Poor eating self-regulation significantly contributed to the weight gain during home confinement (ß = −0.24, p = 0.01). |
|  | Lim SC 2022  Exploring the impact of COVID-19 movement control orders on eating habits and physical activity in low-resource urban settings in Malaysia.  (Also in A9) | Community  N=95  B40 Community via KOSPEN Leaders | Cross-sectional, observational  mixed-method approach (Quantitative /qualitative) delivered via telephone interviews | Movement restrictions significantly reduced the frequency and duration physical activity.  More time was spent in using screen-devices z = 5.479, p < 0.001 Less time doing exercise z = 4.543, p < 0.001 and less frequent exercise sessions z = 3.515, p < 0.003 |
|  | Chin YS 2022  The impact of Movement Control Order during the COVID-19 pandemic on lifestyle behaviours and body weight changes: Findings from the MyNutriLife COVID-19 online survey. | Community  Malaysians aged 18 and above recruited via social media platforms  N=1319  Mean age: 36.3± 1.12  Female: 76.3% | Cross-sectional, observational  Self-reported anthropometric data including body weight and height, and lifestyle behaviours including eating pattern, physical activity, and sleep pattern (new-developed questionnaire) | Lifestyle behaviours including less frequent practice of healthy cooking methods (OR = 1.61, 95% CI = 1.08–2.40) and lunch skipping (OR = 2.39, 95% CI = 1.25–4.60) were associated with weight gain, while less frequent consumption of high fat foods(OR = 0.64, 95% CI = 0.41–0.99), more frequent physical activity of at least 30 minutes/day for at least 5 days/week reduced the odds of weight gain by 45% (OR = 0.55, 95% CI = 0.38–0.79), and good sleep latency (OR = 0.62, 95% CI = 0.43–0.90). were associated with lower risk of weight gain. In contrast, practicing healthy eating concept, skipped lunch, and more frequent physical activity were significantly associated with weight loss. |
|  | Wong KYY 2024  Identifying obesogenic environment through spatial clustering of body mass index among adults  (Also in B3) | Population  Malaysians aged 18 to 59 from population-based censuses National Health and Morbidity Surveys 2014 and 2015  N=17168 (Mean age: 38 ± 11.9 years, Female 51.9%) | Nationwide cross-sectional (Observational)  Ecological study integrating spatial analysis with national survey and administrative data to examine geographic patterns of overweight and obesity | Identified geographic clustering of overweight and obesity in both Peninsula and East Malaysia (Global Moran’s Index 0.12 and 0.15, respectively; +1: strong positive spatial correlation, 0: no correlation, -1: strong negative correlation).  Hotspots with high-BMI clusters were in suburban districts mainly involving females, hotspots in urban areas were greater among males. |
|  | Salway R 2021  The impact of COVID-19 movement restrictions on physical activity in a low-income semi-rural population in Malaysia: A longitudinal study.  (Also in A9) | Community  Adults from Segamat  N=1003 | Retrospective, observational  Physical activity: GPAQ | Physical activity reduced by a mean of 6.7 hours/week (95% confidence interval (CI) = 5.3, 8.0) compared to 2018, with the largest reductions among those in employment. By June, PA was 3.4 hours/week (95% CI = 2.0, 4.8) less than 2018, leaving 34% of adults currently inactive (20% in 2018). |
|  | Sean et al., 2024  Obesity, eating habits, and physical activity before and during COVID-19 pandemic among university lecturers.  (Also in A4,A9) | Community  323 university lecturers from Malaysian universities | Retrospective, observational  - Self-reported questionnaire containing sections on demographics, weight, physical activity, and eating habits changes  - Physical activity level assessed using adapted Johansson & Westerterp questionnaire  - Statistical testing for paired differences (weight changes, physical activity) and associations between weight change and behavioural factors | Baseline: 27.6 % overweight; 13.9 % obese  - During pandemic: overweight prevalence dropped to 24.8 %, obesity rose to 15.2 %  - Mean weight increased by 0.57 ± 0.56 kg during pandemic (statistically significant)  - Physical activity level decreased (from mean 1.67 ± 0.17 to 1.60 ± 0.17)  - Weight change was significantly associated with physical activity, total food intake, and increased consumption of sweetened products / confectionery, salty snacks, fast food, and sweetened drinks |
|  | Chan YY, 2017.  Physical activity and overweight/obesity among Malaysian adults: findings from the 2015 National Health and morbidity survey (NHMS). | Population-based census data from National Health and Morbidity Survey 2015, among adults aged ≥18 years  N=17 261  Mean age not reported  Female: 47.4% | Cross-sectional, observational analysis of objectively measured anthropometry and physical activity levels measured using International Physical Activity Questionnaire (IPAQ). | Men had overall higher physical activity levels than women.  A low level of physical activity was associated with the risk of overweight/obesity (aOR: 1.14, 95%CI: 1.01,1.30) compared to a high level of physical activity among men but not among women |
|  | Mohd Saat NZ, 2021.  Relationship between Physical Activity and Cardiovascular Risk Factors: A Cross-Sectional Study among Low-Income Housewives in Kuala Lumpur. | Community  Housewives from low-income households in Kuala Lumpur.  N=63  Mean age: not reported, majority (41.3%) were aged 51 to 60. | Cross-sectional, observational analysis on sociodemographics, physical activity levels measured using International Physical Activity Questionnaire (IPAQ)-short form and anthropometry, and biomarkers of cardiovascular health. | The study population demonstrated an overall low level of physical activity, with only 70.5 ± 232.4 min/week spent on moderate-to-vigorous physical activity.  Majority (90.5%) of the subjects were rated as having low physical activity, 6.3% were moderate, and only 3.2% were considered as having a high level of physical activity.  Levels of moderate-to-vigorous physical activity was negatively associated with fasting blood glucose (*B*=-0.57 95%CI -0.84,-0.30), and total cholesterol (*B*=-3.69, 95%CI=-4.86,-2.51). |
|  | Chang, 2016.  Domain-specific physical activity among indigenous overweight and obese communities in Sarawak | Community Adults aged 18 to 64 attending a health screening activity, with BMI ≥25kg/m^2^ in villages in Kuching and Samarahan, Sarawak.  N=351  Mean age: 43.8± 11.10  Females: 74.4% | Cross-sectional, observational analysis of sociodemogrphics and physical activity across four domains (leisure, occupational, domestic and transportation) collected using International Physical Activity Questionnaire (IPAQ)- -long form | About half of respondents (53%) were moderately active, with women more active in the domestic domain and men in work and leisure-time domains; younger adults (<30 years) had the lowest physical activity levels. Significant associations were observed between gender and physical activity across work, domestic, and leisure domains, and between occupation and all domains of physical activity. |
|  | Lee YY, 2019.  Associations between self-reported and objectively measured physical activity and overweight/obesity among adults in Kota Bharu and Penang, Malaysia. | Community  Adults aged 20 to 65 years from Kota Bharu, Kelantan and Penang.  N=490  Mean age: 40.0± 14.3 years  Female: 64.7% | Cross-sectional, observational analysis of self-reported versus objectively-measured (using accelerometers) physical activity levels and anthropometry. | There was significant negative association between objectively-measured total moderate-to-vigorous physical activity and BMI. However, there were no significant association between self-reported physical activity with BMI or waist circumference. |
|  | Tan AKG, 2016.  Physical Activity and Body Weight among Adults in Malaysia. | Population  Adults aged 25 to 64 years from Malaysian non-communicable disease surveillance (MyNCDS-1) in 2005/2006  N=2380  Mean age 41.7 15.6 years  Female: 54.3% | Cross-sectional, modelling analysis of treatment-effect model accounting for endogeneity between physical activity levels (measured using IPAQ) and BMI | Only vigorous PA (≥1500 MET-min/week) was significantly associated with lower BMI.  Switching from sedentary to vigorous PA reduced BMI by ~0.74 units (≈1.88 kg); switching from moderate to vigorous PA reduced BMI by ~0.85 units (≈2.14 kg) |
|  | Cheah YK, 2019.  Vigorous and moderate physical activity among overweight and obese adults in Malaysia: Sociodemographic correlates. | Population-based census data from National Health and Morbidity Survey 2015, among adults aged ≥18 years  N=10 141  Average age not reported  Female: 53% | Cross-sectional, obesrvational secondary data analysis of sociodemograhics and participation in moderate-to-vigorous physical activity, stratified by different BMI groups. | Overweight individuals spent the most time in total physical activity (~421 min/week), while obese II spent the least (~343 min/week)  Age increased moderate activity in Obese I but reduced activity in Obese II adults.  Higher income overweight and obese adults spent less time in moderate/vigorous activity compared to lower income peers.  Males engaged in more vigorous and total activity (121–216 min/week more) but less moderate activity than females  Education was only significant for Obese I, where tertiary-educated individuals spent less time in activity than those with primary education.  Being employed was positively associated with all intensity levels of activity across BMI groups. |
|  | Yi LY, 2017.  Accelerometer-Measured Physical Activity and its Relationship with Body Mass Index (BMI) and Waist Circumference (WC) Measurements: A Cross-Sectional Study on Malaysian Adults. | Community  Adults aged 20 to 65 from Kota Bharu, Kelantan and Penang.  N=490  Mean age: 40.0 ± 14.3 years;  Female: 64.7% | Cross-sectional, observational  analysis of levels of moderate-to-vigorous physical activity (MVPA), measured objectively using accelerometers and anthropometry | Participants averaged only 13.5 minutes/day of MVPA, with just 22.7% achieving ≥20 min/day and 11.2% achieving ≥30 min/day. MVPA was higher in men (17.4 vs. 11.3 min/day), in unmarried adults (18.0 vs. 10.6 min/day), and in those from high-walkability neighbourhoods (19.7 vs. 9.1 min/day); total MVPA was inversely associated with BMI (b = –0.045, p < 0.05) but not waist circumference. |
|  | Nik-Nasir, 2022.  Physical Activity in Malaysia: Are We Doing Enough? Findings from the REDISCOVER Study. | Population  Adults aged 18 to 90 years across six states and Federal Territory of Kuala Lumpur  N=7479  Mean age: 51.68 ± 9.5 years  Females: 56.9% | Cross-sectional, observational  Analysis across four domains: of physical activity measured using IPAQ, and associations with sociodemographic factors, anthropometry, and cardiovascular biomarkers. | The median total reported physical activity was 1584.0 (0–5637.3) MET-minute per week. The highest total domain for physical activity was in the domestic domain which is 490 (0–2400) MET-minutes per week.  Factors associated with sufficiently active or very active physical activity include urban residents, Malay ethnicity, no formal education, elementary occupation, current smokers and high high-density lipoprotein.  Low income, male, and normal BMI are less likely to participate in sufficiently active or very active PA. |
|  | Ahmad MH, 2018.  Comparison between self-reported physical activity (IPAQ-SF) and pedometer among overweight and obese women in the MyBFF@home study. | Community  Participants in a community-based intervention study to combat obesity among women with overweight and obesity in Klang Valley.  N=169  Mean age: not reported  Female: 100% | Cross-sectional, observational  comparison (at baseline) between physical activity levels measured by pedometer and self-reported using IPAQ-SF, and longitudinal assessment of pattern of change after six months of intervention. | There were large discrepancies in the classification of ‘active’ via pedometer-measured physical activity (8.4%) versus IPAQ-SF reported physical activity (61.1%), with overall poor agreement (Cohen’s κ = 0.055, p>0.05). After six months, there was 9.4% increment in IPAQ-SF-measured physical activity, but 1.3% reduction in pedometer-measured physical activity. |
|  | Chan WK, 2015.  Low physical activity and energy dense Malaysian foods are associated with non-alcoholic fatty liver disease in centrally obese but not in non-centrally obese patients with diabetes mellitus  (Also in A4) | Clinical  Patients at a diabetes clinic in an academic medical institution in Kuala Lumpur  N=299  Mean age: 63.3±10.5  Female: 59.9% | Cross-sectional, observational  analysis between physical activity levels measured using Global Physical Activity Questionnaire, and dietary composition using a semi-quantitative food-frequency questionnaire, with anthropometry and diagnosis of non-alcoholic fatty liver disease (NAFLD) | Patients with low Physical activity were more likely to have NAFLD (OR:1.75, 95%CI: 1.03,2.99, p=0.029).  Among centrally obese patients, patients with low PA and in the highest quartile of percentage energy intake from fat (OR:4.03, 95% CI:1.12,15.0, p=0.015), high cholesterol food (OR:3.61, 95%CI: 1.37,9.72, p=0.004) and high saturated fatty acid food (OR:2.67, 95%CI: 1.08,6.67, p=0.019) were most likely to have NAFLD.  The same finding was not found in non-centrally obese patients. |
| A8. Determination of genetic factors influencing development of overweight and obesity.  n=16  Data Source:  Community: 5 Others: 1  Study Type:  Cross-sectional: 15 Others: 1  Study Design:  Observational: 15 Others: 1 | Al-Shajrawi et al., 2020  *HIF*1 (rs11549465) and *NFKB1* (rs28362491) variants association with obesity in Malaysia | Community  Malay adults aged 20 to 59 years in Terengganu  N=188  Mean age: 35  Female: 62.8% | Cross-sectional case-control analysis of Hypoxia Inducible Factor-1 - *HIF*1 (rs11549465) and nuclear factor kappa‑beta subunit1- *NFKB1* (rs28362491) genetic polymorphism between normal weight and overweight/obese and association with metabolic profile and anthropometry | HIF1 (rs11549465) showed no significant association, the NFKB1 Ins/Ins genotype was significantly linked with higher obesity risk (p = 0.002), whereas the Del/Del genotype appeared protective. |
|  | Amiratul Athirah et al., 2018  Effects of leptin gene variants on obesity and its attributes in Malay population | Community  Adults aged 18 and above at Terengganu  N=249  Mean age:40.0 ± 1.8  (Female 68.7%) | Cross-sectional, observational  PCR-RFLP technique was performed to determine the genotype and allele distribution of leptin gene variants. | The genotypic and allelic frequencies of leptin gene variants presented no significant difference between groups, G2548A (P = 0.93 and 0.74); H1328080 (P = 0.58 and 0.56); and A19G (P = 0.72 and 0.38) correspondingly.  Statistically significant difference between triglyceride level and genotypes of G2548A variant (P = 0.016); between total cholesterol level and H1328080 genotypes (P = 0.027).  Male gender (aOR= 26.27; CI= 1.06-1.25; P = 0.009), waist circumference (aOR = 1.15; CI = 1.06-1.25; P = 0.001) and body fat percentage (aOR = 1.43; CI = 1.20-1.70; P<0.001) were the independent risk factors for obesity. |
|  | Ching et al., 2023  Interactions of genetic and macronutrient intake with abdominal obesity among middle-aged vegetarians in Malaysia | Community  Adult Vegetarians (Chinese and Indians) from Klang Valley  N=163  Mean age:50 ±5  Female: 69,3% | Cross-sectional, observational  Dietary intakes via food frequency questionnaire.  Anthropometry  Genotypes of the rs174547 of vegetarians were determined by using Agena® MassARRAY | People with TT genotype at higher intake of carbohydrates and protein had higher odds of abdominal obesity (p-interaction <0.05).  The gene-diet interaction remained significant for fibre intake (OR: 4.71, 95% CI: 1.25-17.74, p-interaction = 0.022) among vegetarians with TT genotype at higher intake of fibre after adjusting for age and sex and considering the effects of ethnicity and food groups.  rs174547 significantly interacted with fibre intake on abdominal obesity |
|  | Chong et al., 2018  Association of *IRX3* rs3751723 polymorphism with the risk of overweight and obesity: Case-control study and meta-analysis | Community  Adult Malaysian volunteers  N=1030  Mean age: 26.4 ±11.6 years  Female: 50.1% | Cross-sectional case-control age-matched analysis of Iroquois Homeobox 3(IRX3) rs3751723 polymorphism with sociodemographics and anthropometry. This study also combined with meta-analysis of two additional studies | In Malaysians, homozygous G/G genotype was significantly associated with higher obesity risk (OR:1.72, 95%CI 1.03,2.91).  The G allele increased overweight susceptibility in females but appeared protective in smokers. Paradoxically, those with G/G who did not consume fast food had a four-fold higher obesity risk  The meta-analysis found no significant association between IRX3 rs3751723 and obesity in broader Asian populations across all genetic models |
|  | Chong et al., 2018  Risk association, linkage disequilibrium and haplotype analyses of *FASN* rs4246445, rs2229425, rs2228305 and rs2229422 polymorphisms in overweight and obesity | Community  Malaysian volunteers;  N=1030  Mean age: 23.4 ± 9.8 years  Female: 40.9% | Age- and-sex-matched cross‑sectional comparison between BMI categories on genetic association of Fatty Acid Synthase (*FASN*) single nucleotide polymorphisms (*SNP*), plus haplotype & linkage disequilibrium (LD) analyse, and adjusted for lifestyle factors. | No significant association of any of the four *FASN* SNPs with overweight or obesity status, even combined. SNPs rs4246445 & rs2229425 (and some other combinations) had LD but allele frequencies of some SNPs were very low. Five common haplotypes were identified but none showed significant risk association. |
|  | Hamzah et al., 2024  Genetic variants of obesity in Malaysia: A scoping review | Malaysian studies  N(Studies)=35 | Scoping review of articles published on genetic variants among Malaysians and risk of obesity from Scopus, PubMed and ScienceDirect. | Identified gene variants associated with obesity included: LEPR (K656N), LEP (G2548A—Indian only), *ADIPOQ* (rs17366568), *UCP2* (45bp-I/D), *ADRB3* (rs4994), *MC3R* (rs3827103), *PPARγ* (pro12Ala—Malay only), *IL1RA* (intron 2 VNTR), *NFKB1* (rs28362491), and *FADS1* (rs174547—Indian only) |
|  | Harun et al., 2023  The contribution of *FTO* rs9939609 and *RETN* rs1862513 polymorphisms in predisposing resettled indigenous (Orang Asli) Temiar to metabolic syndrome | Community  Adults from *Temiar* tribe in Kelantan  N=123  Mean age: 36.4±13.7  Female: 69.9% | Cross-sectional, observational  Anthropometrics  Genotyping FTO rs9939609 and RETN rs1862513 polymorphisms using PCR–RFLP | FTO rs9939609 and RETN rs1862513 were associated with risk of MetS susceptibility among the Temiar subtribe with estimated OR 19.9 (P < 0.001) and 20.7 (P = 0.006) for heterozygous (T/A) and homozygous (A/A) genotype at FTO rs9939609 locus, respectively; OR 222.5 (P < 0.001) and 26.2 (P = 0.005) for heterozygous (C/G) and homozygous (G/G) genotype at RETN rs1862513 locus, respectively. |
|  | Lek et al., 2018  Association of dopamine receptor D2 gene (DRD2) Taq1 polymorphisms with eating behaviours and obesity among Chinese and Indian Malaysian university students | Community  Malaysian adults from Kampar  N=594  Mean age: 20.9±0.1  Female 59.13% | Cross-sectional, observational  Anthropometrics  Three Factor Eating Questionnaire-R18.  7-point hedonic scale: preference/intake frequency/craving of 26 common high-fat Malaysian foods was assessed using  Taq1 gene polymorphisms using PCR-RFLP | The overall minor allele frequencies of Taq1A, Taq1B and Taq1D according to ethnicities (Chinese/Indian) were 0.37/0.29, 0.39/0.28, 0.06/0.30, respectively; genotype and allele distributions of Taq1B and Taq1D were significantly different between ethnicities  Those with A1 or B1 allele had lower Cognitive Restraint score and higher Uncontrolled Eating score, while those with A1/A1 or B1/B1 genotype had higher fast food preference. D1 allele was associated with increased starchy food craving and mamak (Malaysian Indian-Muslim) food preference but not eating behaviour scores. |
|  | Lim et al., 2020  Influence of dopamine receptor gene on eating behaviour and obesity in Malaysia | Community  Malaysian adults from Kampar  N=394  Mean age: 23.10 ± 4.70 (male), 22.70 ± 5.36 (Female)  Female: 68.2% | Cross-sectional, observational  Anthropometrics  Eating behaviour: Three-Factor Eating Questionnaire-R18 (TFEQ-R18)  Genotyping of Taq1 gene via PCR RFLP | No significant association between DRD2 gene variants with obesity (p > .05).  ANKK1/DRD2 Taq1A gene polymorphism demonstrated a significant difference in EE score (p < .05).  All three Taq1 gene variants showed no significant difference in CR and UE score (p > .05). |
|  | Mitra et al., 2018  Effect of FTO rs9930506 on obesity and interaction of the gene variants with dietary protein and vitamin E on C-reactive protein levels in multi-ethnic Malaysian adults | Community  Malaysian adults  N=178 | Cross-sectional, observational  Anthropometrics | Malaysian Indians expressed the highest risk allele frequency (G) of FTO rs9930506 compared to the Malays and the Chinese (P < 0.001).  No significant association was found between FTO rs9930506 and obesity (dominant model). Risk allele carriers (G) consumed significantly lower vitamin E (P = 0.020) and had a higher fibre intake (P = 0.034) compared to the noncarriers (A). Gene–diet interaction analysis revealed that risk allele carriers (G) had lower high sensitivity C-reactive protein (hsCRP) levels with higher energy from protein (≥14% day−1; P = 0.049) and higher vitamin E (≥5.4 mg day−1; P = 0.038). |
|  | Mitra et al., 2019  Association of ADRB2 rs1042713 with obesity and obesity-related phenotypes and its interaction with dietary fat in modulating glycaemic indices in Malaysian adults | Community  Malaysian adults aged 18 to 74  N=178  Mean age: 41.67±2.59 years  Female:86.5% | Cross-sectional evaluation of association between *ADRB2* rs1042713 gene with obesity and metabolic parameters, and effect of dietary nutrients on these associations. | Logistic regression revealed no association between ADRB2 rs1042713 and obesity (p=0.725)  Carriers of G allele of rs1042713 were associated with increased odds of insulin resistance (aOR:2.83 95%CI: 1.04,7.70), and among people with obesity, carriers had poor dyslipidemic profile.  Carriers of GG genotype with high polyunsaturated fatty acid intake had lower insulin resistance. |
|  | Mohanraj et al., 2022  Association between leptin (G2548A) and leptin receptor (Q223R) polymorphisms with plasma leptin, BMI, stress, sleep and eating patterns among the multiethnic young Malaysian adult population from a healthcare university | Community  Medical students from a university, aged 18 to 24  N=185 | Cross-sectional, observational  Anthropometrics  Eating pattern: TFEQ Stress: DASS-21 PHQ-9  Leptin gene via PCR RFLP and ELISA | Gender and ethnicity are linked to fasting plasma leptin levels (p < 0.001). No significant association was found between ethnic groups with G2548A LEP (p = 0.338) and Q223A LEPR (p =0.151) polymorphisms  Plasma leptin also affects stress, anxiety, and sadness. Leptin (LEP) and Leptin Receptor (LEPR) polymorphisms were not associated with BMI, plasma leptin, sleep, eating behaviour, or psychological state. |
|  | Ong et al., 2017  Fatty acid translocase gene CD36 rs1527483 variant influences oral fat perception in Malaysian subjects | Community  University Students from Kampar  N=313  Mean age: 20.73±1.5  Female: 62.3% | Cross-sectional, observational  Anthropometry  Ratings of fatty foods: assessed using visual analogue scale | Overall minor allele frequencies for rs1761667 and rs1527483 were 0.30 and 0.26,  Females and individuals with rs1527483 TT genotype significantly perceived greater creaminess of 10% fat-by-weight custard.  Individuals with rs1527483 TT genotype and T allele significantly perceived greater fat content of cream crackers, independent of fat concentration. rs1761667 SNP did not significantly affect OFP, except for cream crackers. Both gene variants were also not associated with obesity measures.  CD36 - specifically rs1527483, plays a role in OFP, but not in influencing obesity |
|  | Shunmugam and Say, 2016  Evaluation of association of ADRA2A rs553668 and ACE I/D gene polymorphisms with obesity traits in the Setapak population, Malaysia | Community  Adults from multiethnic background in Setapak, Selangor.  N=214  Females: 53.7% | Cross-sectional analysis for association of adrenergic receptor α2A (*ADRA2A*) rs553668 and angiotensin-converting enzyme (ACE) I/D single nucleotide polymorphisms (SNPs) with anthropometry | ACE II genotype and I allele were initially linked with greater odds of central adiposity (OR: 2.15 and 1.55, respectively), but this association disappeared after adjusting for age, sex, and ethnicity.  Subjects with the combined *ADRA2A* rs553668 GG and ACE II genotypes had the lowest waist-hip ratio, suggesting a potential protective gene–gene interaction against central obesity |
|  | Tan et al., 2022  Dietary protein interacts with polygenic risk scores and modulates serum concentrations of C-reactive protein in overweight and obese Malaysian adults | Community  Volunteer Malaysian adults from Klang Valley  N=128  Mean age: 44.0 ± 1.0  Female:89% | Cross-sectional, observational  Anthropometry  Food frequency questionnaire  Biochemistry for metabolic and inflammatory biomarkers  Polygenic risk scores (PRS) of rs9930501, rs9930506, and rs9932754 and beta-2 adrenergic receptor rs1042713 | Second- and third-tertile PRS were significantly associated with increased odds of elevated inflammatory status, 7.56 (95% confidence interval [CI], 1.98-28.80; adjusted P = .003) and 3.87 (95% CI, 1.10-13.60; adjusted P = .035), respectively.  Individuals in the third-tertile PRS had significantly lower CRP concentrations (4.61 ± 1.3 mg/L vs 9.60 ± 2.6 mg/L, P = .019) when consuming ≥14% energy from protein (with an average of 18.0% ± 2.4%, 43.0% ± 7.7%, and 39.0% ± 8.0% energy from protein, carbohydrate, and fat per day)  Higher protein intake may alleviate inflammatory status and reduce CRP concentrations systemically in people with high PRS |
|  | Zahary et al., 2020  Increased risk of metabolic syndrome with genetic polymorphism of ADIPOQ among a Temiar population in Malaysia | Community Adult volunteers from *Temiar* tribes in Kelantan  N=123  Mean age: 36.46  Female 69.9% | Cross-sectional, observational  Anthropometry, metabolic biomarkers  ADIPOQ +45T>G and +276G>T polymorphisms via PCR RFLP | genetic polymorphisms at the ADIPOQ +45T>G (rs2241766) and +276G>T (rs1501299) loci increased the odds for development of MetS among the relocated Temiar subtribe  Odds Ratio for the occurrence of MetS with heterozygous (T/G) was 87.2 and with homozygous (G/G) genotype was 26.5 at the ADIPOQ +45T>G locus. Similarly, the OR was 38.2 for heterozygous (G/T) and 19.7 for homozygous (T/T) at the ADIPOQ +276G>T locus |
| A9. Obesity and COVID-19  n=13  Data Source:  Community: 12 Clinical: 1  Study Type:  Cross-sectional: 11 Retrospective: 2  Study Design:  Observational: 13 | Sim et al., 2020  Clinical characteristics and risk factors for severe COVID-19 infections in Malaysia: A nationwide observational study. | Clinical  Patients admitted for COVID-19 aged 12 and above nationwide  N=5889  Median age: 34 years,  Female 28.3% | Cross-sectional observational  Clinical records analysis | Obesity was associated with higher odds of severe COVID [OR:3.20 (95%CI 1.89 - 5.19)] but not significant [aOR: 4.28 (95%CI 0.32–47.67)] after adjusted for age, sex and other comorbidities |
|  | Salway R 2021  The impact of COVID-19 movement restrictions on physical activity in a low-income semi-rural population in Malaysia: A longitudinal study.  (Also in A7) | Community  Adults from Segamat  N=1003 | Retrospective, observational  Physical activity: GPAQ | Physical activity reduced by a mean of 6.7 hours/week (95% confidence interval (CI) = 5.3, 8.0) compared to 2018, with the largest reductions among those in employment. By June, PA was 3.4 hours/week (95% CI = 2.0, 4.8) less than 2018, leaving 34% of adults currently inactive (20% in 2018). |
|  | Ng and Cheng, 2025  Weight gain, poor mental health and increased sedentary hours among Malaysian adults during the COVID-19 pandemic.  (Also in A9) | Community  Malaysian adults aged 18 and above  N=338 | Cross-sectional, Observational  Online questionnaire.  Physical activity and sedentary behaviour were assessed using International Physical Activity Questionnaire-Short Form (IPAQ-SF) while the Perceived Stress Scale (PSS-10), Patient Health Questionnaire (PHQ-9) and Generalised Anxiety Disorder Assessment (GAD-7) were used to examine stress, depression and anxiety, respectively | Average weight gain of 0.6 kg among the participants, 45.5% of them experienced weight gain.  Sedentary behaviour (*p* < 0.001), PSS-10 score (*p* < 0.001), PHQ-9 score (*p* = 0.002) and GAD-7 score (*p* = 0.001) were significantly increased during the COVID-19 pandemic.  Level of physical activity was significantly decreased (*p* = 0.003) during the pandemic.  Weight changes during the pandemic were found to be associated with age, sedentary hours, and PHQ-9 score.  Sedentary hours (AOR = 1.068, 95% CI = 1.002–1.139, *p* = 0.043) were identified to be a risk factor for weight gain during the pandemic. |
|  | Chin et al., 2022  The impact of movement control order during the COVID-19 pandemic on lifestyle behaviours and body weight changes: Findings from the MyNutriLifeCOVID-19 online survey  (Also in A4) | Community  1,319 Malaysian adults | Cross-sectional, observational  Online survey self-reported height, weight, lifestyle behaviours (eating pattern, physical activity, sleep)  Multivariable generalized linear mixed models adjusting for age, sex, ethnicity, baseline weight status | During movement control order  -41.2% of the respondents perceived that their eating patterns were healthier  -36.3% reduced their physical activities  -25.7% had a poorer sleep quality.  -Before MCO: 54.7 % normal weight; 7.8 % underweight; 25.5 % overweight; 12.1 % obese  -During MCO: 30.7 % gained weight (mean +2.1 kg), 32.2 % lost weight (mean −2.3 kg)  -Among overweight respondents, 36.1 % lost weight, 29.4 % gained weight  Lifestyle behaviours including less frequent practice of healthy cooking methods and lunch skipping were associated with weight gain, while less frequent consumption of high fat foods, more frequent physical activity, and good sleep latency were associated with lower risk of weight gain. |
|  | Sean et al., 2024  Obesity, eating habits, and physical activity before and during COVID-19 pandemic among university lecturers.  (Also in A4,A7) | Community  323 university lecturers from Malaysian universities | Retrospective, observational  - Self-reported questionnaire containing sections on demographics, weight, physical activity, and eating habits changes  - Physical activity level assessed using adapted Johansson & Westerterp questionnaire  - Statistical testing for paired differences (weight changes, physical activity) and associations between weight change and behavioural factors | Baseline: 27.6 % overweight; 13.9 % obese  - During pandemic: overweight prevalence dropped to 24.8 %, obesity rose to 15.2 %  - Mean weight increased by 0.57 ± 0.56 kg during pandemic (statistically significant)  - Physical activity level decreased (from mean 1.67 ± 0.17 to 1.60 ± 0.17)  - Weight change was significantly associated with physical activity, total food intake, and increased consumption of sweetened products / confectionery, salty snacks, fast food, and sweetened drinks |
|  | Tan ST., 2022  Screen time-based sedentary behaviour, eating regulation and weight status of university students during the COVID-19 lockdown.  (Also in A4,A7) | Community  186 Malaysian private university students. | Cross-sectional, observational  - Logistic regression (multiple models) adjusting for age, lifestyle, socioeconomics, reproductive factors | Mean non-study internet use: 148 ± 77.7 min/day.  64.5% medium eating self-regulation (mean 3.0 ± 0.5).  Weight gain associated with lower eating self-regulation: β = −0.24, *p* = 0.01; ANOVA F = 5.7, *p* = 0.01 (gainers 2.9 ± 0.5 vs stable/loss 3.2 ± 0.4–0.5).  χ² = 19.9, *p* = 0.01 for association between self-regulation category and weight status.  Screen time not significantly associated with weight change. |
|  | Lim et al., 2022  Exploring the impact of COVID-19 movement control orders on eating habits and physical activity in low-resource urban settings in Malaysia.  (Also in A7) | Community  N=95  B40 Community via KOSPEN Leaders | Cross-sectional, observational  mixed-method approach (Quantitative /qualitative) delivered via telephone interviews | Movement restrictions significantly reduced the frequency and duration physical activity.  There was significantly increased consumption of home-cooked meals  53.7% reduced their consumption of packaged snack foods , street desserts (54.7%), fast food (50.5%), soft drinks (50.5%), and 3-in-1 or instant drinks (50.5%) due to limited access during the MCO. B40 communities were receptive to potential interventions to encourage healthier eating and physical activity leveraging digital approaches under the ‘new normal’. Reported concerns included internet accessibility and affordability, functionality, and digital literacy. |
|  | Tan et al., 2021  Trajectories of Food Choice Motives and Weight Status of Malaysian Youths during the COVID-19 Pandemic  (Also in A4) | Community  Malaysians aged 18-30 Mean age: 22.65 ± 2.46  Female: 64.4% | Cross-sectional, observational  Self-measured anthropometry  38-item food choice questionnaire (FCQ) | 48.6% gained weight due to the confinement, with an average weight gain of 3.90 ± 2.92 kg.  47.0% to 73.0% of the youths changed their food choice motives in the time of COVID-19.  mean scores of health (t = −3.324, p = 0.001), convenience (t = −5.869, p < 0.001), weight control (t = −7.532, p < 0.001), natural content (t = −5.957, p < 0.001), ethical concern (t = −4.419, p < 0.001) and price (t = −3.737, p < 0.001) were significantly higher during the pandemic compared to pre-pandemic  Those highly concerned for weight control were more likely to be in the weight loss category (aOR) = 1.633,95%CI:1.230,2.168, p = 0.001). Conversely, those who gained weight due to the pandemic confinement highly valued natural content in foods (aOR = 0.653,95% CI = 0.481,0.886, p = 0.006) when making their food choices |
|  | Tan et al., 2022  Poor self-regulation in eating behaviour during the COVID-19 lockdown contributes to weight gain among young adults in Malaysia.  (Also in A4) | Community  Young Malaysians | Cross-sectional; observational  Self-reported weight trajectory  International Physical Activity Questionnaire-Short Form (IPAQ-SF)  Self-Regulation of Eating Behaviour Questionnaire (SREBQ) | 82.0% were reported to be physically active during the MCO, with a weekly physical activity intensity of 4551.16 ± 4744.89 MET-minute.  majority (71.0%) had medium self-regulation in eating behaviour during the lockdown, with a mean score of 3.08 ± 0.49. Slightly more than half of the young adults (57.0%) gained weight since the MCO enforcement, with an average weight increment of 1.28 ± 0.60 kg.  Young adults who attained a higher score in SREBQ were more likely in sustained weight (AOR = 2.514, p = 0.024, CI: 1.129–5.597) and weight loss (AOR = 2.624, p = 0.011, CI: 1.250–5.507) categories. |
|  | Tan & Kannan, 2023  Comparison of eating behaviour by relative weight change status of young adults throughout the COVID-19 pandemic  (Also in A4) | Community  Adults aged 18-30 years old free from obesity-related comorbidities, and not participating in weight management interventions throughout the pandemic  N=682 (60.9% Female; Pooled mean age: 22.8±2.5 years) | Cross-sectional, observational  Compare eating behaviour by relative weight change status among young adults in Malaysia throughout the COVID-19 pandemic | 32.1% of adults put on body weight after the pandemic, with an average relative weight gain of 12.44 ± 9.67%.  weight gain category had statistically higher standardised raw scores in uncontrolled eating (49.23 ± 20.84) and emotional eating (48.66 ± 26.41) compared to those in the sustained weight category (uncontrolled eating = 44.42 ± 21.57, p = 0.039 and emotional eating = 42.81 ± 26.30, p = 0.041) |
|  | Abdul Basir et al., 2022  The challenges and strategies towards healthy eating during COVID-19 home confinement period among working adults with BMI ≥ 25 kg/m^2^ enrolled in a weight loss program: qualitative findings.  (Also in A4) | Community  Diet  Adults aged 20-59 years old with a BMI ≥ 25.0 kg/m^2^ (phone interview).  N=11 (54.5% Female, Pooled mean age: 39.6±5.4 years) | Cross-sectional, observational  Challenges and strategies for healthy diets during the first wave of the COVID-19 home confinement period among working adults | Facilitating self-control and self-efficacy in weight-related eating behaviours is imperative during the challenging pandemic-related home confinement period. |
|  | Tan et al., 2022  Changes in dietary intake patterns and weight status during the COVID-19 lockdown: a cross-sectional study focusing on young adults in Malaysia.  (Also in A4) | Community  Diet  Adults aged 18–30 years old from Malaysia (snowball sampling via social media platforms)  N=1045 (63.8% Female, Pooled mean age: 22.7±2.5 years) | Cross-sectional, observational  Changes in dietary intake patterns and their impacts on the body weight status of adults throughout the COVID-19 home confinement. | Overweight/obesity increased from 39.8% (pre-pandemic) to 41.5% (during the pandemic)  Reduced consumption in cereals and grains (β= 0.156, p < 0.001, 95% CI = 0.122–0.288), as well as oils and fats (β= 0.091, p = 0.012, 95% CI = 0.022–0.183), contributed significantly to weight loss during the pandemic confinement |
|  | Moy et al., 2022  Depression and its associated factors among COVID-19 survivors in a middle income country | Community  MH  Adults aged ≥ 18 years old from Malaysia with COVID-19 history.  N=732 (58.7% Female, Pooled mean age: 40.2±10.9 years) | Cross-sectional, observational  Mental health investigation among COVID-19 survivors in the community and its associated factors. | Overweight or obese had higher odds (51.7%, p<0.05)) for mild to severe depression, but comorbidities were not found to be associated with mental health. |

**Supplementary Table 3**: Summaries of key findings of studies on obesity interventions in Malaysia from 2016 to 2024 (Purpose B)

| **Research Scope** | **Study Author(Year)/Title** | **Data Source** | **Design** | **Key Findings** |
| --- | --- | --- | --- | --- |
| B1. Development and evaluation of obesity prevention and intervention programmes.  n=16  Data Source:  Community: 14 Population: 1 Clinical: 1  Study Type:  Cross-sectional: 2 Prospective: 14  Study Design:  Observational: 2 Single-Arm Interventional: 4 RCT: 9 Quasi-Experimental: 1 | Lim KH et al, 2015  Technical report evaluation of effectiveness of implementation of “Komuniti Sihat Perkasa Negara” (KOSPEN) programme in Malaysia- Phase 1 | Community  Villages which had implemented KOSPEN in the state of Johor, Malacca and Negeri Sembilan.  Target populations: 1. Implementers: District Health Officers KEMAS District officers District i-NCD team members  KEMAS staff.  2. Health volunteers  3. Community at KOSPEN localities. | Cohort  Validated tools (self-administered questionnaires and face-to-face interviews) to collect data from the target populations. | 88.2% of District Health Officers, 94.1% of KEMAS District Officers, 86.0% of i-NCD team members and 75.3% of KEMAS staff were aware of their role in implementing the KOSPEN programme.  All District Health Officers and KEMAS District officers, 99.7% of i-NCD team members and 91.5% of KEMAS staff understood the objectives of the KOSPEN Programme.  More than 90% of implementers perceived KOSPEN as a good community intervention programme.  94.1% of District Health Officers, 70.6 % of KEMAS District Officers, 79.7% of i-NCD team members and 73.1% of KEMAS staff said that the KOSPEN programme added value to the existing programme activities in their workplace.  Majority of the volunteers (89.6%) had positive perception of KOSPEN.  Most of them were aware of their roles (96.5%) and functions (94.2%) in implementing KOSPEN programme.  Almost all the volunteers (99.7%) knew of their responsibilities in conducting health screening among the community and to refer those with risk of NCDs to the nearest health clinic.  65.5% of the community from the study area were aware of the existence of KOSPEN programme in which majority of them understood that health screening, physical activity and non-smoking were the core activities of KOSPEN.  All of them had positive perceptions about the programme and its basic elements and they agreed with the practice of drinking plain water and consuming more fruits and vegetables.  Only 45.2% of them participated in KOSPEN activities and 41.1% reported difficulties in participating due to time constraints.  Among those who participated, 88.7% had then practiced drinking plain water and 86.9% consumed more fruits and vegetables |
|  | Lim KK et al, 2021  Non-Communicable Diseases (NCDs) intervention activities before and after the implementation of KOSPEN Plus programme at workplace | Community  All health facilities, government and private agencies in all 16 states in Malaysia that had implemented the KOSPEN Plus programme.  N=362 | Cohort  Self-administered questionnaire consisting of three modules as follows:  i) Information about the respondent and organization.  ii) The eight KOSPEN Plus programme scopes iii) The KOSPEN Plus programme perception. | Majority of the scopes implemented by the facilities/agencies were health screening (95.6%, n = 346), followed by mental health screening (92.0%, n = 333), active life (89.2%, n = 89.2), smoke-free (88.4%, n = 320), healthy eating (82.6%, n = 299) and weight management (82.0%, n = 297).  Generally positive perception toward KOSPEN Plus after the implementation of the programme among the participating facilities/agencies across categories.  Among health facilities (n = 275) which had implemented KOSPEN Plus, the majority of facilities implemented health screening (96.7%, n = 266) followed by mental health (94.5%, n = 260), active living (90.2%, n = 248), smoke free (90.2%, n = 248), healthy eating (83.3%, n = 229) and weight management (82.5%, n = 227)  Among government agencies (n = 74) that implemented the KOSPEN Plus programme, mostly had carried out the health screening scope (90.5%, n = 67), followed by active living (86.5%, n = 64), smoke free (85.1%, n = 63), mental health (83.8%, n = 62), healthy eating (83.8%, n = 62) and weight management (81.1%, n = 60).  Among private agencies (n = 30) that implemented the KOSPEN Plus programme, 100% (n = 30), conducted the health screening scope, followed by active living (84.6%, n = 11), mental health (84.6%, n = 11) and weight management (76.9%, n = 10). |
|  | Supramanian RK et al, 2022  Prevalence of Non-Communicable Disease (Ncd) Risk Factors Among Employees in the Kospen Plus Programme in Malaysia | Population  Employees from workplaces that implemented the KOSPEN Plus programme. | Observational  Retrospective descriptive study of cumulative reporting of NCD risk factors among employees from KOSPEN Plus agencies.  Secondary data over 5 years (2016 till 2020) obtained from the National KOSPEN Plus Programme database were included. | Employees that underwent health screening showed a rising trend since 2016 from 53.5% to 88.2%.  Prevalence of raised random blood glucose levels and raised blood pressure showed a 9.8% increment over 5 years.  Prevalence of overweight individuals ranged from 53.4% to 57.4%.  Prevalence of employees with raised blood pressure was found to be between 12.0% to 17.1%. |
|  | Samsury SF, 2023  The Effectiveness of The Trim and Fit Program in Bodyweight Management Among Healthcare Providers in Terengganu. | Community  HCPs with BMI ≥25kg/m2  N=201 | Randomised controlled trial  Intervention group: Trim and Fit Program emphasizing nutritional modifications, physical activities, recreational engagement, and motivation for six months  n = 100  Control group: Received educational booklets on workplace stress.  n=101 | The Trim and Fit Program significantly improves body weight, body fat percentage, and waist circumference.  Factors associated with successful weight reduction include income, total program attendance, and frequency of calorie intake recording.  Body image perception doesn't significantly contribute to weight reduction success. |
|  | Raman et al, 2024  Assessing the effectiveness of health belief model-based educational interventions on weight control intentions among Malaysians | Community  Malaysian adults aged 18 years and above from both genders, with BMI of 25 kg/m2 and greater or who have the intention to reduce body weight.  N= 134 | Quasi-experimental, prospective  Health Belief Model based educational intervention in the form of graphic visual representations of information in a video.  n = 67  No intervention  n = 67 | Intervention group:  Significant improvement in perceived self-efficacy in dieting (mean score 3.96 ± 0.85) compared to the control group (3.76 ± 0.86, p = 0.003). Increased perceived self-efficacy in exercise (4.12 ± 0.52) compared to the control group (3.51 ± 0.94, p < 0.001). Improved behavioural intention scores improved (4.00 ± 0.59), but the difference was not significant (p = 0.300). |
|  | Nor Hanipah Z et al, 2023  Perception and Barriers to Obesity Management Among People with Obesity and Healthcare Professionals in Malaysia - Awareness Care and Treatment in Obesity Management - Asia Pacific Subgroup Analysis. Malaysian Association for the Study of Obesity (MASO) Scientific Conference 2023; 4-5 September 2023; Putrajaya, Malaysia | Clinical  Online survey among adult patients with obesity (PwO) and healthcare professionals (HCPs)  Adult PwO N = 1001  Healthcare professionals (general practitioners, endocrinologists, obstetricians/gynaecologists, cardiologists, and other appropriate specialities)  N = 200 | Qualitative, cross-sectional | 57% of PwO misclassify their weight status as normal or overweight.  68% of PwO valued discussing weight management with HCPs but success rates remained low.  63% regain weight even after maintaining weight loss for six months or more, key barriers included insufficient exercise, motivation deficits, and poor hunger control.  88% of HCPs recognised obesity as a chronic disease affecting overall health, but patient disinterest, limited awareness of treatment options, and time constraints hindered effective intervention.  70% of PwO trust HCPs’ medication recommendations but only 10% received weight loss prescriptions. |
|  | Mazri FH et al, 2022.  Development and Evaluation of Integrated Chrono-Nutrition Weight Reduction Program among Overweight/Obese with Morning and Evening Chronotypes  (Also in C2) | Community  Overweight/obese non-shift workers from Putrajaya, Malaysia. (Age: 39.6 ± 6.3 years; 74.7% women; BMI: 31.2 ± 4.5 kg/m2)  N = 91 | Single-Arm interventional  Integrated chrono-nutrition weight reduction program (SLIMSHAPE™ Chrono) for 12 weeks | Pre- vs post-intervention  Both chronotypes had increased their % energy intake in the early eating window. (Morning type (MT): 64.8 vs. 67.2%, Evening type (ET): 62.7 vs. 65.6%, Mean difference (MD): 2.8, 95%CI: 0.3, 5.1, p = 0.028).  Both chronotypes reduced their intake in the late eating window, earlier midpoint of eating and had a reduced night eating syndrome score. Late eating window: MT: 35.2 vs. 32.8%, ET: 37.3 vs. 34.4%, MD: −2.8, 95%CI: −5.1, −0.3, p = 0.028 Earlier midpoint of eating: MT: 14:02 vs. 13:49; ET: 14:27 vs. 14:18, 95%CI: −0.4, −0.02, p = 0.029 Night eating syndrome score: MT: 10.0 vs. 8.9; ET: 10.7 vs. 8.9, MD: −1.5, 95%CI: −2.5, −0.5, p = 0.004  No significant change in the first and last mealtime, eating duration and the elapse time between sleep onset and last meal.  ET increased their sleep duration (MD: 0.8 h, 95% CI: 0.4, 1.2, p < 0.001) and reduced social jetlag (MD: 19 min, 95% CI: 1.7, 36.3, p = 0.031) post-intervention compared to MT. |
|  | Heng et al, 2020  The Feasibility of High Intensity Interval Training (HIIT) In the Workplace Obesity Management Program | Community  Employees in a research institute aged 25 to 59 years with BMI ≥ 23 kg/m^2^ in Kuala Lumpur  N=108 | Single-arm interventional  HIIT training 3 times a week, 30 minutes each | Change in anthropometry and body composition  Weight: -2.38% (p<0.001)  BMI: -2.95% (p<0.001)  WC: -0.01% (p=0.905)  Other findings:  Reduction in total cholesterol (-3.10%, p=0.039) |
|  | Cheah et al, 2024  A Multicomponent Workplace Environmental Intervention to Promote Physical Activity among the Staff of Universiti Malaysia Sarawak  (Also in C2) | Community  University employees aged between 20 and 60 years old  N=148 | Randomised controlled trial  Physical activity support and organizational support components  (n-75)  No interventions  (n-73) | Weight and anthropometry changes not measured  Other findings:  The intervention group showed significant improvements in total physical activity self-regulation (PASR) (η_p_^2^=0‧021), goal setting (η_p_^2^=0‧024), total MET-min/week (η_p_^2^=0‧031), housework-related physical activity (η_p_^2^=0‧101), and step/week (η_p_^2^=0‧827) |
|  | Fazliana et al, 2018  Effects of weight loss intervention on body composition and blood pressure among overweight and obese women: findings from the MyBFF@home study | Community  Housewives aged 18 to 59 years old with BMI 25‧0 to 39‧9 kg/m^2^ in Kuala Lumpur  N=328 | Randomised controlled trial  Individual diet counselling, group exercise and self-monitoring tools (food diary and physical activity diary) for 6 months, then followed-up for another 6 months without any intervention as part of maintenance period  (n=169)  A women’s health seminar package  (n=159) | Change in anthropometry and body composition  At 6 months:  Weight: -1‧49% (p<0‧001)  BMI: -1‧55% (p<0‧001)  WC: -3‧16% (p<0‧001)  %BF: -3‧15% (p<0‧001)  Visceral fat area: -3‧47% (p<0.001)  Body fat mass: -3‧46% (p<0.001)  Skeletal muscle mass: +0.22% (p=0‧646)  At 12 months:  Weight: -0‧66% (p<0‧001)  BMI: -0‧64% (p<0‧001)  WC: -4‧32% (p<0‧001)  %BF: -1‧55% (p=0‧152)  Visceral fat area: -2‧58% (p=0‧004)  Body fat mass: -2‧59% (p=0‧002)  Skeletal muscle mass: +0‧90% (p=0‧418)    Other findings:  At six months, both intervention and control groups also reported significant reductions systolic blood pressure (−6‧81, 95%CI −9‧72, −3‧90 mmHg versus −7‧95, 95%CI −11‧69, −4‧20 mmHg, both p<0‧001; between group difference p=0‧629), which were not different between two groups  Intervention group showed a consistent decreasing trend of pain score mainly for back pain. Higher BMI was significantly associated with pain score.  At six months, both intervention and control groups had significantly reduced total cholesterol (-0‧27, 95%CI -0‧42, -0‧11 mmol/L, p=0‧001 and -0‧21, 95%CI -0‧38, -0‧05, p=0‧012, between group difference -0‧06, 95%CI -0‧28,0‧17, p=0‧620) but only intervention group retained the reduction in maintenance phase (-0‧05, 95% CI-0‧19,0‧09 mmol/L, p=0‧488) while the level increased significantly in the control group (+0‧22, 95% CI: 0‧06,0‧38 mmol/L, p=0‧008)  Intervention group showed a trend of reduction in Fasting Plasma Glucose (-0‧11, 95%CI -0‧32,0‧10 mmol/L, p=0‧287) at 6 months and significantly decreased at 12 months (−0‧19 95%CI: – 0‧32,− 0‧06 mmol/L, p=0‧005).  Significant improvement in health-related quality of life (HRQOL) (59‧82±26‧60 to 66‧13±22‧82, p<0‧001) at 6 months. No significant association between weight reduction and HRQOL improvement. |
|  | Kassim et al, 2017  Effects of Lifestyle Intervention towards Obesity and Blood Pressure among Housewives in Klang Valley: A Quasi-Experimental Study (MyBFF@Home) | As above | As above |  |
|  | Mohd Zaki et al, 2018  Impact of community lifestyle intervention on anthropometric parameters and body composition among overweight and obese women: findings from the MyBFF@home study | As above | As above |  |
|  | Mohd Sallehuddin et al, 2018  Changes in body pain among overweight and obese housewives living in Klang Valley, Malaysia: findings from the MyBFF@home study | As above | As above |  |
|  | Ahmad Zamri et a, 2018  Effectiveness of a community-based intervention for weight loss on cardiometabolic risk factors among overweight and obese women in a low socio-economic urban community: findings of the MyBFF@home | As above | As above |  |
|  | Ahmad Zamri et al. 2020  Weight Change and Its Association with Cardiometabolic Risk Markers in Overweight and Obese Women  (Also in A2) | As above | As above |  |
|  | Ambak et al, 2018  The effect of weight loss intervention programme on health-related quality of life among low income overweight and obese housewives in the MyBFF@home study | As above | As above |  |
|  | Abdul Aziz et al, 2018  Influence of co-morbidity on body composition changes after weight loss intervention among overweight housewives: a follow-up study of the MyBFF@home | Community  Control group in the My Body is Fit and Fabulous at Home – without comorbidities (MyBFF@home) Phase II (G1).  (n=84)  Housewives with obesity and co-morbidities (G2).  (n=42)  N=126 | Randomised controlled trial  Both groups received dietary counselling, physical activity (PA) and self-monitoring tools. | Obesity without co-morbidities group (G1) showed a larger decrease in mean visceral fat area compared to the obesity with co-morbidities group (G2) (between group difference: −11‧49, 95%CI − 20‧07, − 2‧91 cm^2^ p=0‧009). |
|  | Saparuddin et al. 2024  Metabolite, Biochemical, and Dietary Intake Alterations Associated with Lifestyle Interventions in Obese and Overweight Malaysian Women | Community  Women with metabolically healthy overweight/obesity (MHO)  (n=36)    Women with metabolically unhealthy overweight/obesity (MUO).  (n=34)  Aged 18 to 59 years old, BMI ≥ 25‧0 to ≤39‧9 kg/m^2^ in Klang Valley  N=70 | Randomised controlled trial  Both groups received intervention consisting of diet control, physical activity and self-monitoring tools (pedometer, physical activity, and diet diaries) | Change in anthropometry and body composition  MUO Group:  Weight: -1‧30%, p=0‧015  BMI: -1‧17% (p=0‧023)  WC: -2‧48%, (p<0‧001)  Other findings:  There were no significant improvements in MHO group.  There was a significant decreasing trend in serum trimethylamine-N-oxide, arginine, ribose, aspartate, carnitine, choline, and tyrosine in the MHO group |
| B2. Development and evaluation of obesity management programmes.  n=23  Data Source:  Community: 10 Clinical: 13  Study Type:  Cross-sectional: 2 Prospective: 20 Retrospective: 1  Study Design:  Observational: 2 Cohort: 5 Single-Arm Interventional: 4 RCT: 10  Quasi-experimental: 1 Mixed/Qualitative: 1 | Al-Tahami BAM et al, 2017  Metabolic and Inflammatory Changes with Orlistat and Sibutramine Treatment in Obese Malaysian Subjects | Clinical  Patients recruited at the Obesity Clinic in Universiti Sains Malaysia’s Hospital  BMI ≥ 27 kg/m2  N = 76 | Randomised controlled trial  Three daily 120 mg dosages of orlistat for nine months  n = 39  once daily 10 or 15 mg dosage of sibutramine for nine months.  n = 37 | For both groups, weight, BMI, WC, BF, VF, HOMA-IR and hs-CRP were significantly lower at the end of the nine month intervention.  For both groups, adiponectin levels and HOMA%S were also significantly increased at the end of the nine month intervention.  No significant differences between the two groups for these parameters with nine months treatment.  Significant decrease in FPG in orlistat group; while fasting insulin and HOMA%B reduced in sibutramine group. |
|  | Zainal Abidin ZA et al, 2017  A randomised controlled trial comparing the use of omega-3 polyunsaturated fatty acid supplements versus very low calorie dietary restriction in obese Malaysian patients awaiting bariatric surgery. | Clinical  Patients aged 18–60 years old with BMI >30 kg/m2 recruited from the obesity clinic of UKM Medical Centre.  N = 41 | Randomised controlled trial  Omega-3-polyunsaturated (PUFA) group  n = 20  Very low calorie diet (VLCD) group  n = 21 | No statistical difference in hepatic volume reduction, ALT levels and significant weight loss between both groups |
|  | Taher MM et al, 2023  Bariatric surgeries: Outcome throughout an annum at a specialist centre in Malaysia. | Clinical  Patients who underwent a single weight reduction surgery (sleeve/gastric bypass) by a single surgeon at the Cengild Medical Centre  N = 1000 | Observational | Mean fasting blood sugar three months post-surgery was 5.84mmol/L vs 10.42mmol/L pre-surgery.  Mean BMI was reduced from 39.69 kg/m^2^ to 27.99 kg/m^2^ after one year post surgery.  Mean difference between 1 month pre-surgery and 3 months post-surgery was 17.32 mmHg for systolic BP and 5.76 mmHg for the diastolic BP. |
|  | Mohd Nasir AAB et al, 2021  A single tertiary centre outlook on the short-term outcome of bariatric surgery in improving pulmonary parameters and sleep apnoea. | Clinical  Patients ⩾18 years attending a bariatric work-up clinic or respiratory clinic or admitted for bariatric surgery work-up with BMI >35 kg/m2 and OSA (AHI >5 events/hour) at Pusat Perubatan Universiti Kebangsaan Malaysia.  N = 12 | Cohort  Patients were assessed at baseline and at 12±2 weeks post bariatric surgery using the following methods: a partial sleep study, lung function test, six-minute walk test (6MWT) and Epworth Sleepiness Scale (ESS) score. | Post surgery (12±2 weeks), the mean BMI decreased from 45.5 kg/m2 to 39.7 kg/m2.  Clinically significant improvement at 3 months post-surgery in:  Apnoeic hypoxic index 24.75 ± 9.51 vs 11.67 ± 5.92 (p < 0.05) Nadir SpO2 (%) 83.6 ± 3.8 vs 85.0 ± 3.3 (p = 0.08) Epworth sleepiness scale score 16 ± 4 vs 7 ± 3 (p = 0.01) FEV1 (L) 2.66 ± 0.35 vs 2.89 ± 0.4 (p = 0.03)  FVC (L) 3.23 ± 0.45 vs 3.42 ± 0.47 (p < 0.05) TLC (L) 4.97 ± 1.19 vs 5.21 ± 1.12 (p < 0.05) ERV (L) 0.51 ± 0.46 vs 0.63 ± 0.43 (p < 0.05) RV (L) 1.46 ± 0.91 vs 1.64 ± 0.92 (p < 0.05) Adjusted DLCO (ml/mmHg/min) 22.71 ± 5.22 vs 22.84 ± 5.19 (p = 0.04) Adjusted DLVA (ml/mmHg/min/L) 5.61 ± 0.90 vs 3.23 ± 1.16 (p = 0.002) 6MWT (m) 293 ± 49 vs 337 ± 48 (p<0.05) |
|  | Roslan MH et al, 2023  Cardiac and Metabolic Effects of Bariatric Surgery Among Obese Patients in a Malaysian Tertiary Hospital: A 6-month Prospective Cohort Study. | Clinical  Patients about to undergo bariatric surgery  N = 28 | Cohort  Patients underwent echocardiography at baseline and 6 months after surgery with a focus on diastolic function measurements and global longitudinal strain (GLS). They also had fasting serum lipid and glucose measurements pre- and post-surgery. | Mean weight loss after surgery was 24.1 kg (p < 0.001).  Mean reduction in BMI 8.59 kg/m^2^ (p < 0.001)  54% (n = 15) had diastolic dysfunction before surgery, which reduced to 7.1% (n=2) after surgery.  Mean indexed left atrial volume reduced to 27.1 ml/m^2^ from 32 ml/m^2^ pre-surgery.  Mean E/e' reduced to 11.78 post-surgery from 13.43 pre-surgery.  Mean left ventricular global longitudinal strain was (-)25.7% post-surgery from (-)21.2% pre-surgery.  Mean reduction in LDL: 0.85 mmol/L (p < 0.001)  Mean reduction in total cholesterol: 0.83 mmol/L (p < 0.001)  Mean reduction in triglycerides 0.17 mmol/L (p < 0.001)  Mean reduction in SBP 19.8 mmHg (p < 0.001)  Mean reduction in DBP 12.2 mmHg (p < 0.001) |
|  | Pang WS et al, 2024  Comparative evaluation of early diabetic outcomes in southeast Asian patients with type 2 diabetes mellitus undergoing Roux-en-Y gastric bypass (RYGB) versus sleeve gastrectomy (LSG). | Clinical  Obese patients above 18 years of age with T2DM who underwent either laparoscopic Roux-en-Y gastric bypass (LRYGB) or laparoscopic sleeve gastrectomy (LSG) at Pusat Perubatan Universiti Kebangsaan Malaysia  N = 172 | Cohort  LRYGB (n = 72)  LSG (n = 100) | T2DM remission for patients who underwent either surgical technique for mild, moderate or severe disease was 92.9%, 56.2% and 14.7% respectively.  Both surgical techniques improved T2DM control for patients in the study.  Comparing baseline with results 1 year postoperatively,  Median HbA1c reduced from 7.40% (IQR 2.60) to 5.80% (IQR 0.80) (p < 0.001)  Mean total antidiabetic medications use reduced from 1.48 (SD 0.99) to 0.60 (SD 0.86) [p < 0.001]  Insulin usage reduced from 27.9 to 10.5% (p < 0.001)  T2DM control improved from 27.9 to 82% (p < 0.001).  Median excess BMI loss: 69.4% (IQR 34%) and 53.2% (IQR 36.0%) for RYGB and SG respectively (p = 0.016).  At one year following surgery, there is no difference between LRYGB and LSG in terms of diabetic remission. |
|  | Fazliana M et al, 2023  Molecular, Metabolic, and Nutritional Changes after Metabolic Surgery in Obese Diabetic Patients (MoMen): A Protocol for a Multicentre Prospective Cohort Study. | Clinical | Cohort |  |
|  | Ab Majid NL et al, 2024  Post bariatric surgery complications, nutritional and psychological status. | Clinical  Post-bariatric surgery patients who had undergone surgery for a maximum of five years at Hospital Universiti Sains Malaysia.  All the types of bariatric surgery cases were included, no restrictions on gender  N = 63 | Observational  Cross-sectional study where patients were interviewed via several platforms such as WebEx Meeting, Zoom Meeting, and Google Meet | Most common complications experienced by patients after bariatric surgery were hair loss (50.8%), gastroesophageal reflux disease (GERD) (49.2%), and vomiting (41.3%).  There were significant differences in mean weight before (129.5 (33.0) kg/m2) and after (85.0 (32.0) kg/m2) bariatric surgery (p<0.001).  Prevalence of clinically severe obesity declined by 55%.  Patients had insufficient intake of protein, fat, calcium, and iron.  Majority of the patients experienced a normal level of stress, anxiety, and depression.  Some patients had mild (3.2%), moderate (4.8%), and severe anxiety (1.6%) |
|  | Subramaniam K et al, 2018  Eating Behaviour Predicts Weight Loss Six Months after Bariatric Surgery: A Longitudinal Study. | Clinical  Patients with obesity who underwent bariatric surgery for weight loss in two tertiary referral hospitals in Kuala Lumpur  N = 57 | Cohort  Laparoscopic Roux-en-Y gastric bypass (n = 30)  Laparoscopic sleeve gastrectomy (n = 23)  Laparoscopic one anastomosis gastric bypass–mini gastric bypass (n = 4) | Mean excess BMI loss (EBMIL) and total weight loss (TWL) at the sixth month was 63.31% and 23.83%, respectively.  Anxiety, depression, and external eating scores reduced over time.  Advancing age, high BMI, and higher scores for emotional and external eating were significant negative predictors for TWL%. |
|  | Yue TP et al 2022  Food tolerance, nutritional status and health-related quality of life of patients with morbid obesity after bariatric surgery. | Clinical  Patients with morbid obesity aged 18-60 years old undergoing elective bariatric surgery at Hospital Kuala Lumpur  N = 90 | Cohort  Prospective observational study | Excess weight loss one month and three months after surgery was 14.1% and 31.6% respectively.  Significant decrease in the total food tolerance score from 26.4 (SD ¼ 0.8) at baseline to 17.7 (SD ¼ 4.7) one month after bariatric surgery, but a gradual rise in the score to 21.7 (SD ¼ 4.3) three months after surgery.  A similar pattern is seen in the dietary intake assessment where a significant reduction in the total energy from 1842 kcal (SD ¼ 445) at baseline to 570 kcal (SD ¼ 180) one month and an increase to 731 kcal (SD ¼ 185) three months after bariatric surgery.  HRQOL of the study patients showed a statistically significantly increase in the domain of physical health (t ¼ 7.253, p ¼ < 0.001), psychological (t ¼ 7.692, p < 0.001), social relationship (t ¼ 5.767, p < 0.001) and environment (t ¼ 4.208, p < 0.013) three months after bariatric surgery. |
|  | Gullaam Rasul SF et al, 2022  Lived Experience after Bariatric Surgery among Patients with Morbid Obesity in East Coast Peninsular Malaysia: A Qualitative Study. | Clinical  Patients aged ≥18 who had bariatric surgery at least six months ago at Hospital Universiti Sains Malaysia.  N = 21 | Cohort, Qualitative  Phenomenological approach by exploring post-bariatric patients’ stories, empowering them to share their very personal life experiences and challenges before and after the surgery | Before surgery, patients experienced social restraint due to physical limitations, difficulty in performing daily and religious activities, lack of suitable public facilities, and limited clothing options. They also faced bias and stigmatisation, including body shaming from family, discrimination at work, and negative public reactions.  After surgery, patients reported improvements in physical health, regained the ability to perform religious rituals and enjoyed enhanced sexual intimacy, leisure activities, and new hobbies. They also had improved self-esteem.  Some patients reported side effects such as gastroesophageal reflux disease and hair loss. Some patients regained weight. |
|  | Kosai NR et al, 2024  One-Year Outcomes in a Supervised Program of Swallowable Intragastric Balloon-Analysis of 486 Patients in a High-Volume Bariatric Centre in Malaysia. | Clinical  Patients aged between18 and 65 years old, BMI ≥ 27kg/m^2^ who underwent swallowable intragastric balloon insertion at a teaching hospital in Malaysia  N = 486 | Cohort  Prospective data collection of consecutive patients | Mean starting BMI of 35.3 ± 7.2 kg/m2 decreased to 31.5 ± 5.7 kg/m2 (p < 0.0001) at the end of 4 months and further decreased to 30.3 ± 5.4 kg/m2 (p < 0.0001) at the end of 12 months.  Combined mean %TBWL was 10.5% at 4 months and 13.7% at 12 months.  The %TBWL was 15.6% for males, 13.3% for females at 12 months.  Patients with a higher starting BMI demonstrated a significantly higher %TBWL compared to the lowest BMI category of < 29.9 kg/m^2^ at both 4 and 12 months. |
|  | Saad et al, 2021  The Effects of a 7000-Step Goal and Weekly Group Walking Program for Overweight and Obese Elderly People in Sarawak, Malaysia: A Quasi-experimental Study | Community  Older adults aged 60 years to 75 years old with BMI > 24‧9 kg/m^2^  N=109 | Quasi-experimental  Combination of gradual walking program that used pedometer feedback as a  motivation tool and weekly group activities.  (n=48) | Change in anthropometry and body composition  Weight: -3‧25% (p<0‧001)  BMI: -3‧27% (p<0‧001)  WC: -3‧13% (p<0.001)  Visceral Fat %: -9‧58% (p<0‧050)  Skeletal Muscle %: +6‧68% (p<0‧001) |
|  | Kok et al, 2018  A Pedometer-based Intervention with Daily Walking Steps and its Relationship with Nutritional Status among Overweight/Obese University Students in Kuala Terengganu | Community  University students in Terengganu  BMI > 25 kg/m^2^  N=23 | Single-arm Experimental  Participants needed to reach the targeted steps according to the plan of the intervention phases where each phase lasted for 2 weeks. The intervention for was modified to participants’ baseline step count and increased by 10% every 2 weeks.  (n=8) | Change in anthropometry and body composition  Weight: not reported  BMI: -1‧55% (p=0‧017)  WC: 3‧85% (p=0‧080) |
|  | Nik Yahya et al, 2019  A feasibility of simulation-based exercise programme for overweight adult in higher learning institutions  (Also in C2) | Community  Students in two higher learning institutions in Kuantan, Pahang, Aged > 18  BMI ≥23 kg/m^2^  N=52 | Single-arm Experimental  A booklet and CD to do aerobic and resistance exercise for a minimum of 25 minutes per day, 3  times a week (n=27) | Change in anthropometry and body composition  Weight: not reported  BMI: -2‧68 % (p<0‧001)  WC: -1‧38% (p=0‧001)  %BF: -2‧96% (p=0‧001) |
|  | Chen et al, 2021  Effects of Brisk Walking on Plasma Lipoprotein(a), Total Antioxidant Status, Aerobic Fitness, Percent Body Fat, Waist Circumference and Resting Blood Pressure in Overweight and Obese Females  (Also in C2) | Community  Female participants from Universiti Sains Malaysia  Aged 20 to 35 years old with BMI 23‧0 to 40‧0 kg/m^2^  N=38 | Randomised controlled trial  Brisk walking exercise conducted 3 times per week for 6 weeks (n=19)  Refrain from any other exercise programme and continued their normal diet  (n=19) | Change in anthropometry and body composition  Weight: not reported  BMI: not reported  WC: -3‧48% (p<0‧001)  %BF: -3‧71% (p<0‧001)  Other findings:  Both groups had significantly reduced plasma lipoprotein(a) level (p<0‧001). Intervention group also had significant reductions in resting blood pressure (p<0‧001). Both groups had no significant differences in total antioxidant status and aerobic fitness after the brisk walking programme. |
|  | Abdullah et al, 2024  The Effect of a Combined Intermittent Fasting Healthy Plate Intervention on Anthropometric Outcomes and Body Composition Among Adults with Overweight and Obesity: Nonrandomized Controlled Trial  (Also in C3) | Community  Aged 19 to 59 years old with BMI ≥ 23 kg/m^2^  N=177 | Randomised controlled trial  Combined intermittent fasting and healthy plate (IFHP) group: observe dry fasting from dawn to dusk for 2 days a week and practice HP for the rest of the week  (n=91)  Healthy plate (HP) group: division of plate portions into a quarter for protein, a quarter for complex carbohydrates, and a half for fruits and vegetables for at least 1 main meal per day  (n=86) | Change in anthropometry and body composition  Weight: -1‧81% (p=0‧003)  BMI: -1‧76% (p=0‧005)  WC: -1‧75^% (p=0‧001)  %BF: -4‧01% (p<0‧001)  Visceral fat area: -4‧78% (p<0‧001)  Skeletal muscle mass: +0‧01% (p>0‧999)  Other findings:  HP group showed significant reduction in HC (−1‧034cm; p=0‧030) but no significant reduction in weight, BMI, %BF, body fat mass, and visceral fat area, and no significant increase in skeletal muscle mass. |
|  | Tan ST et al, 2022  Effectiveness of an integrated-weight management programme (i-WMP) in reducing body weight among noncommunicable disease patients in Malaysian government primary care clinics: a randomised controlled trial  (Also in C2) | Clinical  Patients attending primary health clinics with NCD aged 18 and above and BMI ≥ 25 kg/m^2^ in Selangor  N=244 | Randomised controlled trial  Group-based intervention developed based on behaviour change wheel and operationalization of behaviour change techniques delivered over 3 sessions (n=122)  Standard of care (n=122) | Absolute changes in anthropometry and body composition not reported.  Other findings:  Based on Generalized Linear Mix Model (GLMM) analysis, the intervention was significantly effective in reducing weight F (2,499) = 16.020; p < 0.001, BMI, F (2, 499) = 16.711, p<0.001; WC (F (2, 499) = 16.767, p < 0.001, and improving various diet and physical activity measures. Only %BF and MET-minutes/week were not significantly improved. |
|  | Rusali et al, 2018  Comparison of the Effectiveness of Online and Face-to-Face Weight-loss Interventions in the Workplace: Evidence from Malaysia | Community  Adults aged 20 to 59 years old with BMI ≥ 24‧9 kg/m^2^ in Klang Valley  N=108 | Randomised controlled trial  Face-to-face intervention (FT): Structured weight reduction program called Slim Shape Module^TM^ consisting of 16 two-hour sessions (32 hours) of talks, demonstrations, interactive activities, hands-on activities and exercise sessions  (n=38)  Online intervention (OG): Diet, behavioural and physical activity delivered via an online weight education program  (n=31)  Printed booklets with educational information about diet, physical activity, and behaviour modification for weight reduction given during the first meeting (n=39) | Change in anthropometry and body composition  Weight: -1‧39% (OG) (p=0‧027), -7.08% (FT) (p=0‧001)  BMI: -1‧24% (OG), -7‧9% (FT) (p=0‧002)  WC: -4‧36% (OG), -8‧03% (FT) (p=0‧194)  %BF: -1‧30% (OG), -6‧47% (FT) p=<0‧001  Other findings:  FT group had significantly greater reduction in triglycerides (-35%), total cholesterol to HDL-C ratio (-7‧1%), energy intake (-703kcal/day), carbohydrates (-38‧7 %) and fat intake (-41‧2%) and significant increase in HDL-C (+7‧7%) as compared with OG and control group. (All p<0‧050) |
|  | Jamal et al, 2016  Effectiveness of a Group Support Lifestyle Modification (GSLiM) Programme among Obese Adults in Workplace: A Randomised Controlled Trial | Community  Employees from a local university with BMI > 27‧5 kg/m^2^ in Kuala Lumpur  N=194 | Randomised controlled trial  Group Support Lifestyle Modification (GSLiM) activities including self-monitoring, cognitive-behaviour sessions, exercise as well as dietary change advocacy, which were conducted through seminars and group sessions  (n=97)  Dietary counselling (n=97) | Change in anthropometry and body composition  Weight: -2‧94% (p<0‧001)  BMI: -2‧96% (p<0‧001)  WC: -1‧58% (p<0‧001)  Other findings:  19‧6 % of the participants in the GSLiM group achieved 6 % weight loss compared to 4‧1 % in the comparison group (risk ratio 4‧750; 95% CI: 1‧680 - 13‧450). |
|  | Mohamed et al, 2018  Benefits of Community Gardening Activity in Obesity Intervention: Findings from F.E.A.T. Programme | Community  Aged between 25 to 59 years old with BMI > 23 kg/m^2^  N=61 | Randomised controlled trial  Fit, Eat, Active and Training (F.E.A.T) programme: focusing on nutrition education, interactive and informative education sessions and participation in physical activity and exercise training, featuring community gardening as a medium  (n=31)  No intervention  (n=30) | Change in anthropometry and body composition  Weight: -3‧50% (p<0‧050)  BMI: -4‧10% (p<0‧050)  WC: not reported  Other findings:  Intervention group had significantly greater vegetable intake (1‧8 ± 0‧7 serving size) as compared to control group (0‧7 ± 0‧5 serving size) (p<0‧050) |
|  | Roslan et al, 2019  Effects of an 8-week community exercise program on health-related physical fitness in overweight and obese working adults  (Also in C1) | Working adults aged 18 to 50 years old with BMI 23 to 34‧9 kg/m^2^  N=185 | 8-week exercise program for 60 minutes/day, 3 days/week  (n=90)  Briefing regarding physical fitness, health-related benefits and advised to exercise regularly for 8 weeks  (n=103) | Significant differences in the mean improvement between intervention and control group in cardiorespiratory fitness, muscle strength, muscle endurance, flexibility, and the reduction in %BF (all p<0‧001).  No significant increase in lean mass between groups (p=0‧218). |
|  | Thamby et al, 2017  Effectiveness of an Interventional Educational Tool on Certain Overweight and Obesity Parameters Among the General Public in Sungai Petani, Kedah Darul Aman, Malaysia | Community  Malaysian citizens residing in Sungai Petani for the last three generations, aged between 18 to 30 years old with BMI of > 25‧0kg/m^2^  N=551 | Randomised controlled trial  Pre-validated educational handouts given over 3 phases, 6 months apart | Change in anthropometry and body composition  Weight: -10% (p<0‧001),  BMI: -9% (p<0‧001)  WC: not measured  Other findings:  35% increase in knowledge score (p<0‧001), 43.3% increase dietary habits assessment score (p<0‧001) and 17‧85% increase in physical activities score (p<0‧001) |
| B3. The impact of policies and environment (food and physical activity) on obesity  n=15  Data Source:  Population: 5 Community: 7 Others: 3  Study Type:  Cross-sectional: 9 Prospective: 2 Retrospective: 1 Other: 3  Study Design:  Observational: 6 Cohort: 1 RCT: 1 Mixed/Qualitative: 4 Review: 2 Modelling: 1 | Wong KYY et al, 2024.  Identifying obesogenic environment through spatial clustering of body mass index among adults  (Also in A7) | Population  Malaysians aged 18 to 59 from population-based censuses National Health and Morbidity Surveys 2014 and 2015  N=17168 (Mean age: 38 ± 11.9 years, Female 51.9%) | Observational, Cross-sectional  Nationwide cross-sectional ecological study integrating spatial analysis with national survey and administrative data to examine geographic patterns of overweight and obesity | Identified geographic clustering of overweight and obesity in both Peninsula and East Malaysia (Global Moran’s Index 0.12 and 0.15, respectively; +1: strong positive spatial correlation, 0: no correlation, -1: strong negative correlation).  Hotspots with high-BMI clusters were in suburban districts mainly involving females, hotspots in urban areas were greater among males. |
|  | Goh EV et al, 2020.  The nutrition transition in Malaysia; key drivers and recommendations for improved health outcomes. | Population  Population data from Food Balance Sheets, Malaysian Adult Nutrition Survey and National Health and Morbidity Surveys from 1980 to 2014.  (Sample size not specified) | Modelling study, Retrospective  Analysis of interactions between changes in food supplies and dietary trends and progression of metabolic diseases and risk factors, parallel with demographic and socioeconomic transitions. | Energy supply for the population remained consistently in excess of average caloric needs by at least 30%.  There is shifting food trends with increased supply of wheat (+56.5%), sugar and sweeteners (+23.9%), meat (+49.3%), fish and seafood (+38.7%), eggs (+55.7%) and decrease in supply of rice (-23.7%). There is increasing preference towards animal protein over plant protein. |
|  | Eng CW, 2022.  Dietary practices, food purchasing, and perceptions about healthy food availability and affordability: a cross-sectional study of low-income Malaysian adults. | Community  Low-income adults from bottom 40% of the economic spectrum in Kuala Lumpur.  N=2983 (Median age: 36; Interquartile range: 26-49, Female: 50.2%) | Observational, Cross-sectional  Face-to-face interviews using a structured questionnaire on dietary practices, perceptions of healthy food availability and affordability and factors affecting food-purchasing decisions. | Majority (89.5%) of B40 adults did not consume adequate daily amounts of fruits and vegetables, 68.1% consumed sugar-sweetened beverages at least once a week.  Majority reported healthy foods were moderately available and priced.  Factors that affect food purchase decisions include price (79.4%), availability (75%), and taste (73%) |
|  | Nik Rosmawati et al, 2017  Impact of food nutrition intervention on food handlers’ knowledge and competitive food serving: a randomized controlled trial | Community  Food handlers from 16 primary school canteens located in Kota Bharu,  Kelantan  N=79 | Randomised controlled trial, Prospective  Food Nutrition Intervention targeting knowledge on food nutrition, healthy food choices, portion sizes and meal planning, encouraging vegetable and milk/milk product servings and reducing unhealthy options.  (n=33)  Usual food handling and serving practices (n=46) | Anthropometry not measured  Other findings:  Intervention group had significantly greater knowledge about healthy food choice (p<0‧001) and composite knowledge at 12 weeks post intervention than at baseline (p=0.001).  Intervention group had significantly increased number of vegetable menu served by six weeks (p=0‧040) and significantly increased number of milk and milk products served by 12 weeks (p=0‧015) as compared to the control group. |
|  | Sundram TKM et al, 2022.  Effects of Ambient Particulate Matter (PM2.5) Exposure on Calorie Intake and Appetite of Outdoor Workers | Community  Working adults in Kuala Lumpur aged 18 ≥years from 2016 to 2017 | Prospective cohort  Cohort study on changes in dietary habits among outdoor workers versus indoor workers in relation to changes in exposure to air pollutants. | Long hours of outdoor work corresponding to high PM_2.5_ exposure during haze periods correlated with increasing appetite and caloric intake |
|  | Shyam S et al, 2019  Developments in the implementation of sugar-sweetened beverage tax in Malaysia-A narrative review | Others | Review, Cross-sectional | The review collates the preparatory considerations preceding the implementation of the Sweetened Beverages Excise Duty, the proposed objectives of the tax, its format as reported in the media.  The early sentiments expressed by the stakeholders in the duration leading to the implementation and immediately after (10 days’ post-implementation) the enforcement of the duty are also presented. |
|  | Ng S et al, 2021  Tracking Progress from Policy Development to Implementation: A Case Study on Adoption of Mandatory Regulation for Nutrition Labelling in Malaysia | Community  Case participants held senior positions in the Federal government (n = 6), food industry (n = 3) and civil society representations (n = 3) | Mixed/ Qualitative, Cross-sectional  Semi-structured interviews, together with the review of documents cited by interviewees. | International directions stimulated policy processes in Malaysia, but policy inertia caused implementation gaps.  Barriers hindering policy processes included lack of resources, governance complexity, lack of monitoring, technical challenges, policy characteristics linked to costing, lack of sustained efforts in policy advocacy, implementer characteristics and/or industry resistance, including corporate political activities (e.g., lobbying, policy substitution).  Facilitators to the policy processes were resource maximization, leadership, stakeholder partnerships or support, policy windows and industry engagement or support.  Progressing policy implementation required stronger leadership, resources, inter-ministerial coordination, advocacy partnerships and an accountability monitoring system |
|  | Pondor I et al, 2017.  Higher Dietary Cost Is Associated with Higher Diet Quality: A Cross-Sectional Study among Selected Malaysian Adults. | Community  Adults aged 18 to 64 in Selangor  N=450  (Mean age: 37.55 ± 11.0; Females: 64.2%) | Cross-sectional, observational  Analysis of cost of daily dietary cost (DDC) and diet quality assessed using Malaysian Healthy Eating Index (M-HEI) (0=low diet quality, 100=high diet quality). | Mean M-HEI score: 61.31±10.88, with energy-adjusted DDC was RM10.71/2000 kcal (USD 2.49). People with high levels of DDC had higher M-HEI scores (Quintile 1: 57.14 ± 10.07 versus Quintile 5: 63.26 ± 11.54, *p* = 0.001).  Higher dietary cost is associated with healthy eating. |
|  | Ng S et al, 2018  Extent of implementation of food environment policies by the Malaysian Government: gaps and priority recommendations | Community  Local experts  N=26  Academia/professionals n=11  Non-government/non-profit organisation n=15 | Mixed/ Qualitative, Cross-sectional  The Healthy Food-Environment Policy Index (Food-EPI) comprises 47 indicators of government policy practice. Local evidence of each indicator was compiled from government institutions and verified by related government stakeholders. The extent of implementation of the policies was rated by experts against international best practices. | Majority (62 %) of indicators was rated ‘low’ implementation.  No indicator rated as either ‘high’ or ‘very little in terms of implementation.  The top five recommendations were  (i) restrict unhealthy food marketing in children’s settings and (ii) on broadcast media (iii) mandatory nutrition labelling for added sugars (iv) designation of priority research areas related to obesity prevention and diet-related non-communicable diseases (v) introduce energy labelling on menu boards for fast-food outlets. |
|  | Ng S et al, 2021  Policy Inertia on Regulating Food Marketing to Children: A Case Study of Malaysia | Others  Participants representing the Federal government (n = 5), food industry (n = 2) and civil society (n = 2) were interviewed  N=9 | Mixed/ Qualitative, Others  Qualitative methods, including historical mapping, semi-structured interviews with key informants and a search of cited documents. | More barriers than facilitators in the policy process led to industry self-regulations.  Cited barriers were the lack of political will, industry resistance, complexity of legislation, technical challenges, and lack of resources, particularly professional skills.  The adoption of industry self-regulation created further barriers to subsequent policy advancement, including implementer indifference (industry), lack of monitoring, poor stakeholder relations, and policy characteristics linked to weak criteria and voluntary uptake.  These underlying barriers, together with a lack of sustained public health advocacy, exacerbated policy inertia.  Key recommendations include strengthening pro-public health stakeholder partnerships, applying sustained efforts in policy advocacy to overcome policy inertia, and conducting monitoring for policy compliance and accountability |
|  | Ng S et al, 2020  Benchmarking the transparency, comprehensiveness and specificity of population nutrition commitments of major food companies in Malaysia | Population  Major food and non-alcoholic beverage manufacturers (n = 22)  Quick service restaurants (n=5)  Retailers (n=6) | Mixed/ Qualitative, Others  Business Impact Assessment on population nutrition and obesity (BIA-Obesity) tool has good practice indicators for food industry commitments across six domains (n = 6). These were adapted to the Malaysian context.  Evidence of commitments, including from national and international entities, were compiled from publicly available information for each company.  Expert Panel (≥ 5 members for each domain) assessed commitments and disclosures collected against the BIA-Obesity scoring criteria.  Review Panel, comprising of the Expert Panel and additional government officials (n = 13), then formulated recommendations. | Median overall BIA-Obesity score was 11% across food industry sectors with only 8/33 companies achieving a score of > 25%.  Participating (p < 0.001) and global (p = 0.036) companies achieved significantly higher scores than non-participating, and national or regional companies, respectively.  Highest scoring domain: corporate strategy related to population nutrition. (Median score of 28%)  Lowest scoring domain: product formulation, accessibility, and promotion. (Median scores < 10%)  Recommendations: The establishment of clear targets for product formulation Strong commitments to reduce the exposure of children to promotion of unhealthy foods. |
|  | Lee YY et al, 2020  The Role of Public Health Dietary Messages and Guidelines in Tackling Overweight and Obesity Issues | Others  Global Burden of Disease Study 2014  National Health and Morbidity Survey 2015 | Review, Others  Public health messages related to nutrition and obesity in Malaysia, public health spending in Malaysia, evolution and controversy in dietary guidelines, and the controversial role of the food industry in public health dietary messages were reviewed | Public health dietary communication in Malaysia has progressed and improved substantially over the years.  Most messages have been designed for the general audience, with little consideration of differences in physical, social, cultural, and environmental backgrounds, and varying levels of comprehension.  Recommend three broad strategic actions:  i. building capacity and receptivity for complex ideas  ii. mobilising a diversity of messengers iii. implementing key policy interventions that target the food environment |
|  | Abdullah Z et al, 2022  Contrariwise obesity through organic food consumption in Malaysia: a signalling theory perspective  (Also in A5) | Community  Respondents from selected healthy Facebook communities such as ‘Diet Suku Suku Separuh’, ‘Hiking, and Camping around Malaysia’, and ‘Healthy Malaysia’  N=241 | Modelling study, Cross-sectional  Data from survey using 42 assessed questions, analysed using PLS (SEM) approach | Health consciousness does not significantly predict the purchase intention of healthy food.  All antecedents were significant contributors to the prediction of foods’ purchase intentions.  No positive relationship exists between brand image identifications and brand credibility identifications, and healthy foods’ purchase intentions identifications.  No positive relationship exists between health consciousness identifications and healthy foods’ purchase intentions identifications |
|  | Mohamad Hasnan AN et al, 2023  Ever-never heard of ‘Malaysian Healthy Plate’: How many people know and practice this healthy eating concept? | Population  National Health and Morbidity Survey 2019 | Observational, cross-sectional  Questionnaires pertained to ever heard, knowledge, and practised of Malaysian Healthy Plate were asked along with another questionnaire | Estimated 16.9 million (79.6%) adults in Malaysia were never heard of the Malaysian Healthy Plate Concept.  20.4% or an estimated 4.3 million adults who ever heard of Malaysian Healthy Plate.  Among those who ever heard, 80.8% (3.5 million) correctly interpreted or understood the concept and only 70.5% (2.5 million) have been practising it daily or occasionally |
|  | Tan LK et al, 2023  Does Awareness of Malaysian Healthy Plate Associate with Adequate Fruit and Vegetable Intake among Malaysian Adults with Non-Communicable Diseases? Nutrients | Population  National Health and Morbidity Survey 2019 | Observational, cross-sectional  Awareness of the Malaysian Healthy Plate (MHP) concept was assessed by the question “Have you ever heard of the ‘Malaysian Healthy Plate’/‘Quarter Quarter Half?’”, which had a binary response option (“Yes” or “No”)  Fruit and vegetable (FV) intake of the respondents was assessed using the following four questions: Q1: “In a typical week, how many days did you consume fruits?”; Q2: “Usually on the day that you eat fruits (e.g., apple, orange, banana and so on), how many servings did you take?”; Q3: “In a typical week, how many days did you eat cooked and/or raw vegetables?”; and Q4: “Usually on the day that you eat cooked and/or raw vegetables, how many servings did you take?”. | MHP awareness is associated with adequate FV intake among Malaysian adults with abdominal obesity (adjusted odds ratio (aOR): 1.86, 95% confidence interval (CI): 1.05–3.29), diabetes mellitus (aOR: 4.88, 95% CI: 2.13–22.18), hypertension (aOR: 4.39, 95% CI: 1.96–9.83), and hypercholesterolemia (aOR: 4.16, 95% CI: 1.48–11.72). |

**Supplementary Table 4**: Summaries of key findings of studies developing new modalities for investigating obesity in Malaysia from 2016 to 2024 (Purpose C)

| **Research Scope** | **Study Author(Year)/Title** | **Data Source** | **Design** | **Key Findings** |
| --- | --- | --- | --- | --- |
| C1. Identification of new methods to define obesity  n=3  Data Source:  Community: 2 Population: 1  Study Type  Cross-sectional: 1 Others: 3  Study Design:  Modelling Studies: 2 Observational: 1 | Safaei M et al, 2022  A Hybrid MCDM Approach Based on Fuzzy-Logic and DEMATEL to Evaluate Adult Obesity | Population  Physicians and healthcare professionals at Malaysian public and private universities  N=26 | Modelling study  Collected data were analysed with the DEMATEL and Fuzzy Rule-Based methods | Lack of physical activity, insufficient sleep, unhealthy diet, genetics, and perceived stress were the most significant risk factors for obesity |
|  | Wong JE et al, 2022  Predicting Overweight and Obesity Status Among Malaysian Working Adults with Machine Learning or Logistic Regression: Retrospective Comparison Study | Community  Participants in the Malaysia’s Healthiest Workplace by AIA Vitality 2019 survey  Mean age 34.2, SD 9.0 years; 41% male (n=6904); 41.8% (n=7048) with overweight or obesity  N=16860 | Modelling study  Predictor variables were modelled using the extreme gradient boosting (XGBoost), random forest (RF), and support vector machine (SVM) algorithms, as well as LR, to predict overweight or obesity status based on a BMI cutoff of 25 kg/m^2^ | Area under the receiver operating characteristic curve was 0.81 (95% CI 0.79-0.82), 0.80 (95% CI 0.79-0.81), 0.80 (95% CI 0.78-0.81), and 0.78 (95% CI 0.77-0.80) for the XGBoost, RF, SVM, and LR models, respectively.  Weight satisfaction was the top predictor, and ethnicity, age, and gender were also consistent predictor variables of overweight or obesity status in all models |
|  | Moy and Loh, 2015  Cardiometabolic risks profile of normal weight obese and multi-ethnic women in a developing country  (Also in A2) | Community  Female teachers from 70% of public secondary schools from six states in Peninsular Malaysia.  N = 6854 | Cross-sectional, observational  Complex sample analyses to determine the associations between lifestyle behaviours and cardiometabolic risks according to %BF tertiles of the normal weight participants. Multivariate logistic regression to estimate the odds ratio of cardiometabolic risks across %BF tertiles, adjusted for age and ethnicity | 1095 of 6854 women with normal BMI. Prevalence of normal weight obese was 19.8% (95% CI: 17.3–22.5).  NWO was more prevalent among the Indians and older women.  NWO women had higher odds for abdominal obesity (OR: 2.64, 95% CI: 1.73–4.04), hypertriglyceridemia (OR: 2.51, CI: 1.47–4.29) and hypertension (OR: 1.63, CI: 1.15–2.31) compared to women with lower % body fat after adjusted for age and ethnicity. The prevalence of metabolic syndrome among NWO women was 5.4% (95% CI: 3.0–9.8).  None of the lifestyle behaviours were significantly associated with NWO. |
| C2. Identification of novel strategies to prevent and manage obesity.  N=15  Data Source:  Community: 11 Clinical: 4  Study Type:  Cross-sectional: 5 Prospective: 10  Study Design:  Observational: 3 Single-Arm Interventional: 3 RCT: 7 Mixed/Qualitative: 2 | Mazri FH et al, 2022.  Development and Evaluation of Integrated Chrono-Nutrition Weight Reduction Program among Overweight/Obese with Morning and Evening Chronotypes  (Also in B1) | Community  Overweight/obese non-shift workers from Putrajaya, Malaysia.  Age: 39.6 ± 6.3 years; 74.7% women; BMI: 31.2 ± 4.5 kg/m2  N = 91 | Single-arm interventional  Integrated chrono-nutrition weight reduction program (SLIMSHAPE™ Chrono) for 12 weeks | Post- vs pre-intervention  Both chronotypes had increased their % energy intake in the early eating window. (Morning type (MT): 64.8 vs. 67.2%, Evening type (ET): 62.7 vs. 65.6%, Mean difference (MD): 2.8, 95%CI: 0.3, 5.1, p = 0.028).  Both chronotypes reduced their intake in the late eating window, earlier midpoint of eating and had a reduced night eating syndrome score. Late eating window: MT: 35.2 vs. 32.8%, ET: 37.3 vs. 34.4%, MD: −2.8, 95%CI: −5.1, −0.3, p = 0.028 Earlier midpoint of eating: MT: 14:02 vs. 13:49; ET: 14:27 vs. 14:18, 95%CI: −0.4, −0.02, p = 0.029 Night eating syndrome score: MT: 10.0 vs. 8.9; ET: 10.7 vs. 8.9, MD: −1.5, 95%CI: −2.5, −0.5, p = 0.004  No significant change in the first and last mealtime, eating duration and the elapse time between sleep onset and last meal.  ET increased their sleep duration (MD: 0.8 h, 95% CI: 0.4, 1.2, p < 0.001) and reduced social jetlag (MD: 19 min, 95% CI: 1.7, 36.3, p = 0.031) post-intervention compared to MT. |
|  | Mazri FH et al, 2024  Early-Day Protein Intake Influences the Transition of Metabolic Health Phenotypes among Adults with Obesity  (Also in A4) | Community  Overweight/obese non-shift workers from Putrajaya, Malaysia.  Age: 39.6±6.3 years; 74.7% women; body mass index (BMI): 31.2±4.5 kg/m2  N= 91  MHO: BMI≥25kg/m2 with fewer than three metabolic abnormalities  n= 50  MUO: BMI≥25kg/m2 with three or more metabolic abnormalities  n = 41 | Single-arm interventional  Integrated chrono-nutrition weight reduction program (SLIMSHAPE™ Chrono) for 12 weeks | Post- vs pre-intervention  24% of the participants transitioned from MUO to MHO or from MUO/MHO to metabolically healthy normal weight (MHN).  MUO proportion decreased to 31%.  Participants who demonstrated positive changes were characterized with highest increase in energy intake from protein sources (+5.2% vs +1.1%, p=0.004), particularly during the earlier part of the day (+4.3% vs +1.1%, p=0.029), greater body weight loss (-7.0% vs -3.2%, p=0.022), body fat loss (-3.4% vs -1.3%, p=0.013) and visceral fat loss (-1.3 vs -0.5, p=0.013) as well as a higher proportion of morning chronotype (72.7% vs 35.7%, p=0.033) |
|  | Yaacob et al, 2016  Dumbbells and ankle-wrist weight training leads to changes in body composition and anthropometric parameters with potential cardiovascular disease risk reduction | Community  Aged 18 to 60 years old with BMI ≥ 23 kg/m^2^  N=89 | Single-arm Interventional  Participants in the dumbbell (DB) group performed structured group exercises 3 times per week using a pair of 1kg dumbbells (n=40)  Participants in the ankle-wrist weight (AW) group were given one pair of 500g ankle weights and one pair of 500g wrist weights to be worn during the activities of daily living three days per week for at least 20 minutes (n=49) | Change in anthropometry and body composition  Weight: not reported  BMI: -0‧20% (AW), -0‧60% (DB) (p=0.662)  WC: -3‧82% (AW), -4‧44 % (DB) (p<0.001)  %BF: -2‧95% (AW) -3‧23% (DB) (p<0.001)  Skeletal Muscle %: +3‧12% (AW) 3‧30% (DB) (p<0‧001)  Other findings:  Intervention group had significant reduction in BMI only at week six (-0‧23kg/m^2^; p=0‧007). |
|  | Roslan et al, 2019  Effects of an 8-week community exercise program on health-related physical fitness in overweight and obese working adults  (Also in B2) | Community  Working adults aged 18 to 50 years old with BMI 23 to 34‧9 kg/m^2^  N=185 | Randomised controlled trial  8-week exercise program for 60 minutes/day, 3 days/week  (n=90)  Briefing regarding physical fitness, health-related benefits and advised to exercise regularly for 8 weeks  (n=103) | Significant differences in the mean improvement between intervention and control group in cardiorespiratory fitness, muscle strength, muscle endurance, flexibility, and the reduction in %BF (all p<0‧001).  No significant increase in lean mass between groups (p=0‧218). |
|  | Abdullah et al, 2024  The Effect of a Combined Intermittent Fasting Healthy Plate Intervention on Anthropometric Outcomes and Body Composition Among Adults with Overweight and Obesity: Nonrandomized Controlled Trial  (Also in B2) | Community  Aged 19 to 59 years old with BMI ≥ 23 kg/m^2^  N=177 | Randomised controlled trial  Combined intermittent fasting and healthy plate (IFHP) group: observe dry fasting from dawn to dusk for 2 days a week and practice HP for the rest of the week  (n=91)  Healthy plate (HP) group: division of plate portions into a quarter for protein, a quarter for complex carbohydrates, and a half for fruits and vegetables for at least 1 main meal per day  (n=86) | Change in anthropometry and body composition  Weight: -1‧81% (p=0‧003)  BMI: -1‧76% (p=0‧005)  WC: -1‧75^% (p=0‧001)  %BF: -4‧01% (p<0‧001)  Visceral fat area: -4‧78% (p<0‧001)  Skeletal muscle mass: +0‧01% (p>0‧999)  Other findings:  HP group showed significant reduction in HC (−1‧034cm; p=0‧030) but no significant reduction in weight, BMI, %BF, body fat mass, and visceral fat area, and no significant increase in skeletal muscle mass. |
|  | Mitra et al, 2019  Effect of an individualised high-protein, energy-restricted diet on anthropometric and cardio-metabolic parameters in overweight and obese Malaysian adults: a 6-month randomised controlled study | Community  Aged >18 years old with BMI ≥ 23 kg/m^2^  N=128 | Randomised controlled trial  Hipcref (high-protein, energy-restricted, high-vitamin E and high-fibre) diet charts based on their personal preferences (n=65)  Generalised dietary advice based on Malaysian Dietary Guidelines 2010 (n=63) | Change in anthropometry and body composition  Weight: -4‧58% (p<0‧001)  BMI: -4‧73% (p<0‧001)  WC: -6‧65% (p<0‧001)  %BF: -5‧60% (p<0‧001)  Other findings: Control group had significant increase in weight (+1‧76%, p=0‧012) and BMI (+2‧04%, p=0‧007) from baseline to month six  Intervention group showed 33‧3% (p<0‧001) reduction in HOMA-IR and 23‧0% (p=0‧020) reduction in hsCRP |
|  | Verma RK et al, 2018  Perceptions Of the Malaysian General Public on Community Pharmacy-Based Weight Management Services | Community  Members of the public aged ≥ 18 years old who had visited community pharmacies previously for health-related matters  N = 730 | Observational  Self-administered questionnaire comprising of sections that focused on public preferences and options on weight management approaches, perceived availability of extended services and resources provided by community pharmacists in relation to weight management, utilization of these services and resources, and factors influencing acceptability of weight management services provided by community pharmacists. | Majority (37.7%) ranked dietitians as their preferred first line of consultation, followed by fitness instructors (29.9%) and doctors (13%).  Majority perceived that community pharmacies they had visited offered services for measuring health indicators such as blood pressure (n=591, 81%),blood glucose (n=581,79.6%)and blood cholesterol (n=532, 72.9%).  n=337, 46.2% perceived the availability of more direct weight management services such as measurement of BMI ,waist/hip circumference (n=261, 35.8%) and body fat percentage (n=250,34.2%). Slightly less than half of the respondents felt that community pharmacies provided advice on physical activity (n=353,48.4%)and healthy eating (n=337, 46.2%), or referrals to other healthcare professionals for weight management (n=189,25.9%).  More than half of the respondents reported that community pharmacies had supplements (n = 553, 75.8%) and oral medications (n=388, 53.2%) for reducing weight.  Majority of the respondents (n=470, 64.4%) perceived that community pharmacists should provide weight management services, and 40.7% (n=297) perceived that community pharmacists were trained to manage overweight and obese patients. |
|  | Verma RK et al, 2019  Attitudes, Practices, and Barriers of Malaysian Community Pharmacists Toward Provision of Weight Management Services | Community  Community pharmacists (CPs) who worked across six states of Malaysia (Selangor, Federal Territory of Kuala Lumpur, Pulau Pinang, Johor, Sabah, and Melaka)  N = 550 | Observational  Self-administered questionnaire consisting of 49 statements/questions and responses were given through a five-point Likert scale. It comprised of four sections:   i. The characteristics and sociodemographic data of CPs ii. CPs’ attitudes regarding obesity and overweight, including on causes of overweight and obesity, effectiveness of overweight and obesity management and their confidence toward overweight and obesity management iii. The weight management practices of CPs, including anthropometric/physiological services, advises and sale of products iv. The barriers CPs perceived in providing WMS in community pharmacies | n = 312, 56.7% strongly agreed that overeating and n = 297, 54.0% strongly agreed that sedentary lifestyles contribute to obesity and overweight.  n = 285, 51.9% strongly agreed that exercise training is an effective weight reduction strategy.  n = 231, 42% disagreed/strongly disagreed that surgery is an effective weight reduction strategy.  n = 308, 56.0% agreed/strongly agreed that a lack of staff was a barrier to providing weight management services.  n = 285, 51.9% agreed/strongly agreed that ethical and legal issues associated with sales of products/drugs for obesity management was a barrier to providing weight management services.  CPs who were aged < 30 years expressed significantly stronger agreement that medication adherence is beneficial for weight loss compared to those CPs between 41–50 years.  CPs who were pharmacy owners provided significantly more frequent BMI measurement and patient information materials as part of their weight management practices compared to CPs who worked as a part timer/locum |
|  | Blebil AQ et al, 2022  Using COM-B Model in Identifying Facilitators, Barriers and Needs of Community Pharmacists in Implementing Weight Management Services in Malaysia: A Qualitative Study | Community  Community pharmacists with a minimum experience of 1 year, in Kuala Lumpur and Selangor  N = 25 | Qualitative  In-depth, semi-structured interviews with CPs. The thematic analysis approach was used to analyse the data, and the themes generated were mapped onto COM-B model components | Themes were (1) motivation of pharmacists and (2) knowledge and skills, which were identified as both barriers and facilitators, and (3) the barriers to implementation in aspects of social norms and resources.  Factors were subsequently categorised into the subcomponents of the model: physical capability (e.g., training), psychological capability (e.g., lack of knowledge), physical opportunity (e.g., product range), social opportunity (e.g., stigma), automatic motivation (e.g., remuneration) and reflective motivation (e.g., CPs extended roles) |
|  | Verma RK et al, 2021  Impact of an Educational Training Program on the Knowledge, Attitude, and Perceived Barriers of Community Pharmacists Towards Obesity and Overweight Management in Malaysia | Community  CPs who were currently practicing in Klang Valley, Malaysia  N = 30 | Single-arm Interventional  One-day educational training program delivered by several experts in weight management, consisting of pharmacists, a nutritionist, and a dietitian. | Mean knowledge score increased both immediately after (14.93 ± 1.62) and 30 days following the intervention (17.04 ± 2.51).  The increment was statistically significant 30 days following the intervention (p = 0.001) compared to both pre-intervention and immediate-post intervention stages.  The participants had a more positive attitude towards the provision of weight management service (WMS) in community pharmacies.  They had significantly stronger perceptions about the importance of their role to manage overweight and obesity and their professional competence to treat obese patients.  The barrier of not having space in pharmacy to perform proper counselling for weight management and the barrier of not having training sessions in the area of obesity management were perceived to be significantly less important post-intervention. |
|  | Pitil et al, 2023  The impact of acceptance and commitment therapy (ACT) on the psychological defense mechanism and weight loss program: A randomized controlled trial among university students during COVID-19 movement control order | Community  University students with BMI ≥ 23‧0 kg/m^2^ who reported high immature and neurotic defense styles  N=152 | Randomised controlled trial  ACT program designed to promote psychological flexibility that encourages openness, awareness, and engagement  ACT Group: Six sessions of at least 1.5 hours per session, once a week (n=52)  ACT-Exercise (ACT-Ex) group: In addition to the ACT sessions, an exercise program was conducted three times per week via a one-hour virtual session (n=50)  No interventions (n=50) | Change in anthropometry and body composition  Weight: -4‧55% (ACT), -7‧79% (ACT-Ex), p=0‧001  BMI: -4‧14% (ACT), -7‧73% (ACT-Ex), p=0‧001  WC: not reported  Other findings:  Both interventions were also found to have improved psychological defense mechanism, including experiential avoidance. |
|  | Nik Yahya et al, 2019  A feasibility of simulation-based exercise programme for overweight adult in higher learning institutions  (Also in B2) | Community  Students in two higher learning institutions in Kuantan, Pahang, Aged > 18  BMI ≥23 kg/m^2^  N=52 | Single-arm Interventional  A booklet and CD to do aerobic and resistance exercise for a minimum of 25 minutes per day, 3  times a week (n=27) | Change in anthropometry and body composition  Weight: not reported  BMI: -2‧68 % (p<0‧001)  WC: -1‧38% (p=0‧001)  %BF: -2‧96% (p=0‧001) |
|  | Yunus NA et al, 2023  The perceptions of healthcare practitioners on obesity management in Peninsular Malaysia: a cross-sectional survey | Clinical  Healthcare practitioners commonly involved in obesity management in Peninsular Malaysia from both the public and private healthcare sectors.  N=209 | Observational  Online survey exploring practitioners’ perceptions, barriers and needs in managing obesity, and evaluated weight stigma using the Universal Measures of Bias – Fat (UMB Fat) questionnaire | Majority (n = 196, 94.3%) agreed that obesity is a chronic disease, perceived a responsibility to provide care (n = 176, 84.2%) and were motivated to help patients to lose weight (n = 160, 76.6%).  22% (n = 46) thought their patients were motivated to lose weight.  Most frequently reported barriers to obesity discussions were short consultation time, patients’ lack of motivation, and having other, more important, concerns to discuss.  Mean (SD) of the Universal Measures of Bias – Fat summary score was 2.99 (0.87), with the mean (SD) domain scores ranging between 2.21 and 4.36 (1.06 to 1.45).  No demographic and clinical-related factors were significantly associated with negative judgment. |
|  | Yunus NA et al, 2023  Patients' Experience of Accessing Healthcare for Obesity in Peninsular Malaysia: A Qualitative Descriptive Study | Clinical  Patients aged 18 years and over diagnosed with obesity (BMI ≥27.5 kg/m^2^) and received healthcare for obesity at least once in Peninsular Malaysia  N=22 | Qualitative  Semi structured interview guide informed by the literature and Levesque’s framework. The interviews started with general questions before moving on to the details about participants’ experiences, from perceiving the care to benefiting from it | Five themes were identified: (1) moving from perceiving the need to seeking obesity care is a non-linear process for patients, (2) providers’ words can inspire patients to change, (3) patients’ needs and preferences are not adequately addressed in current obesity care, (4) over-focusing on weight by patients and healthcare providers can lead to self-blame and loss of hope for patients and (5) obesity healthcare can have consequences beyond weight loss. |
|  | Chen et al, 2021  Effects of Brisk Walking on Plasma Lipoprotein(a), Total Antioxidant Status, Aerobic Fitness, Percent Body Fat, Waist Circumference and Resting Blood Pressure in Overweight and Obese Females  (Also in B2) | Community  Female participants from Universiti Sains Malaysia  Aged 20 to 35 years old with BMI 23‧0 to 40‧0 kg/m^2^  N=38 | Randomised controlled trial  Brisk walking exercise conducted 3 times per week for 6 weeks (n=19)  Refrain from any other exercise programme and continued their normal diet  (n=19) | Change in anthropometry and body composition  Weight: not reported  BMI: not reported  WC: -3‧48% (p<0‧001)  %BF: -3‧71% (p<0‧001)  Other findings:  Both groups had significantly reduced plasma lipoprotein(a) level (p<0‧001). Intervention group also had significant reductions in resting blood pressure (p<0‧001). Both groups had no significant differences in total antioxidant status and aerobic fitness after the brisk walking programme. |
|  | Cheah et al, 2024  A Multicomponent Workplace Environmental Intervention to Promote Physical Activity among the Staff of Universiti Malaysia Sarawak  (Also in B1) | Community  University employees aged between 20 and 60 years old  N=148 | Randomised controlled trial  Physical activity support and organizational support components  (n-75)  No interventions  (n-73) | Weight and anthropometry changes not measured  Other findings:  The intervention group showed significant improvements in total physical activity self-regulation (PASR) (η_p_^2^=0‧021), goal setting (η_p_^2^=0‧024), total MET-min/week (η_p_^2^=0‧031), housework-related physical activity (η_p_^2^=0‧101), and step/week (η_p_^2^=0‧827) |
|  | Tan ST et al, 2022  Effectiveness of an integrated-weight management programme (i-WMP) in reducing body weight among noncommunicable disease patients in Malaysian government primary care clinics: a randomised controlled trial  (Also in B2) | Clinical  Patients attending primary health clinics with NCD aged 18 and above and BMI ≥ 25 kg/m^2^ in Selangor  N=244 | Randomised controlled trial  Group-based intervention developed based on behaviour change wheel and operationalization of behaviour change techniques delivered over 3 sessions (n=122)  Standard of care (n=122) | Absolute changes in anthropometry and body composition not reported.  Other findings:  Based on Generalized Linear Mix Model (GLMM) analysis, the intervention was significantly effective in reducing weight F (2,499) = 16.020; p < 0.001, BMI, F (2, 499) = 16.711, p<0.001; WC (F (2, 499) = 16.767, p < 0.001, and improving various diet and physical activity measures. Only %BF and MET-minutes/week were not significantly improved. |
|  | Kong et al, 2017  Worksite weight management program: A three-months intervention study in a primary health care setting | Clinical  Employees from Bintulu government polyclinic aged 18 to 59 years with BMI ≥ 18‧5 kg/m^2^ in Sarawak  N=88 | Randomised controlled trial  Modification of dietary intake by community registered dietitians and increasing high-intensity interval training (HIIT) with motivational interviewing (n=43)  Traditional counselling and weekly aerobic exercise from medical officer and physiotherapist. (n=45) | Change in anthropometry and body composition  Weight: -6‧80% (p=0‧010)  BMI: not reported  WC: -8‧37% (p=0‧010)  HC: -7‧05% (p=0‧010)  Other findings:  Intervention group demonstrated a significant improvement in all cardiometabolic risk factors [total cholesterol (-22‧7 %, p=0‧010), LDL (-30‧5 %, p=0‧01), HDL (+20.8%, p=0‧010), fasting blood sugar (-4‧9%, p=0‧040), 2-hour oral glucose tolerance test glucose (-8‧3%, p=0‧010), HbA1c (-4.8%, p=0‧010)]. |
